# Genetic and Phenotypic Investigations of Viral Subpopulations Detected in Different Tissues of Laying Hens Following Infectious Bronchitis Virus Infection

**DOI:** 10.3390/v17040527

**Published:** 2025-04-04

**Authors:** Ahmed Ali, Ryan Rahimi, Motamed Elsayed Mahmoud, Adel A. Shalaby, Rodrigo A. Gallardo, Mohamed Faizal Abdul-Careem

**Affiliations:** 1 Faculty of Veterinary Medicine, University of Calgary, Calgary, AB T2N 4N1, Canada; ahmed.ali@ucalgary.ca (A.A.); ryan.rahimi@ucalgary.ca (R.R.); motamed.ali@ucalgary.ca (M.E.M.); 2 Department of Pathology, Faculty of Veterinary Medicine, Beni-Suef University, Beni Suef 62511, Egypt; adel.shalabi@vet.bsu.edu.eg; 3 Department of Animal Husbandry, Faculty of Veterinary Medicine, Sohag University, Sohag 84524, Egypt; 4 Department of Population Health and Reproduction, School of Veterinary Medicine, University of California, 1089 Veterinary Medicine Dr. VM3B, Davis, CA 95616, USA; ragallardo@ucdavis.edu

**Keywords:** infectious bronchitis virus, spike 1, single nucleotide variants, next-generation sequencing, subpopulations

## Abstract

Infectious bronchitis virus (IBV) commonly produces a range of genetic sequences during replication, particularly in the spike 1 (S1)-coding portion of the S gene, leading to distinct subpopulations within the broader viral population. It has been shown that certain microenvironments exert selective pressure on the S1-coding sequences and their encoded proteins, influencing the selection of viral subpopulations in these environments. In this study, high-throughput next-generation sequencing (NGS) was used to analyze the S1-coding sequences from tissues of the respiratory, digestive, renal, and reproductive systems of specific pathogen-free (SPF) laying hens. These tissues were collected nine days after infection with the California 1737/04 (CA1737/04) IBV strain, which is known to cause varying degrees of pathology in these tissues. Using a specific bioinformatics pipeline, 27 single nucleotide variants (SNVs) were detected in the S1-coding sequences derived from different tissues. These SNVs shaped multiple subpopulations (SP1–SP15), with SP1 being the core subpopulation present in all tissues, while others were tissue-specific. The IBV RNA loads in the tissues were negatively correlated with the number of SNVs or the Shannon entropy values, and phylogenetic analysis revealed a genetic divergence in the S1-coding sequences from certain tissues with lower viral RNA loads, particularly those from the trachea and ovary. Furthermore, the SNVs were associated with nonsynonymous mutations, primarily located in hypervariable region 2 (HVR 2) within the N-terminal domain of S1 (S1-NTD), except for those in SP7, which was exclusive to the trachea and contained changes in HVR 3 in the C-terminal domain of S1 (S1-CTD). Overall, this study adds to the existing knowledge about IBV evolution by highlighting the role of tissue-specific environments in shaping viral genetic diversity.

## 1. Introduction

Avian infectious bronchitis virus (IBV) is the causative agent of a highly contagious disease in chickens, leading to significant economic losses in the modern poultry industry [1]. IBV is an enveloped, single-stranded, positive-sense ribonucleic acid (RNA) virus with a genome length of about 27.6 kb, serving as the prototype of the *Gammacoronavirus* genus within the Coronaviridae family [2]. IBV predominantly targets the respiratory tract in chickens, causing severe respiratory disease and spreading via respiratory and fecal–oral routes. Additionally, IBV can impact the urogenital, digestive, and reproductive systems, leading to conditions such as nephritis, salpingitis, proventriculitis, and egg drop, depending on the specific IBV strain [2,3].

The genome of IBV contains at least 10 open reading frames (ORFs) flanked by two untranslated regions (UTRs), arranged in the following order: 5′ UTR-1a-1ab-Spike (S)-3a-3b-Envelope (E)-Membrane (M)-5a-5b-Nucleocapsid (N)-3′ UTR [4]. Ribosomal frameshifting from the 1a reading frame into the 1ab ORF at the 5′ end produces two polyproteins, 1a and 1ab. These polyproteins are subsequently cleaved by two types of virus-encoded proteases, resulting in the formation of 15 non-structural proteins (nsps), specifically nsps 2–16, which are essential for the virus’s replication and pathogenicity [4,5]. The remaining 3′ portion of the genome encodes structural proteins, such as S, M, E, and N proteins, along with small non-structural accessory proteins (3a, 3b, 4b, 4c, 5a, 5b, and 6b) whose functions are not well understood [6]. The S glycoprotein, encoded by the S gene, undergoes post-translational cleavage by proteolysis at the furin consensus site (RRFRR/HRRR), producing S1 and S2 subunits, which mediate viral receptor binding and entry into the host cell [7].

The IBV genome, like those of other RNA viruses, is characterized by a high rate of substitutions [8] and recombination [9,10], leading to the continuous emergence of new genetic and antigenic variants. The estimated substitution rate of the IBV genome is 10^−4^ to 10^−5^ substitutions per site per year [11]. Similar to other RNA viruses, IBV is prone to errors during replication due to the limited proofreading capability of the RNA-dependent RNA polymerase (RdRp) and the absence of RNA repair mechanisms [12,13]. This leads to a complex distribution of mutants (i.e., viral subpopulations) within the broader viral isolate [14,15].

The S1 glycoprotein contains the receptor-binding site and is critical for tissue tropism and the elicitation of protective immunity [7]. It contains two main domains: the N-terminal domain (NTD), spanning amino acids 21–237, and the C-terminal domain (CTD), spanning amino acids 269–414; additionally, it includes two subdomains, SD1 and SD2 [16]. Both S1-NTD and S1-CTD may serve as receptor-binding domains (RBDs), playing a crucial role in the binding of the virus to host cells [16]. Furthermore, the S1 subunit contains three hypervariable regions (HVRs): HVR 1 (amino acids 38–67), HVR 2 (amino acids 91–141), and HVR 3 (amino acids 274–387) [17,18]. The HVRs are crucial for viral infectivity, containing at least five neutralization sites [19] and serotype-specific epitopes [20].

The microenvironment of each organ can significantly impact the selection of viral mutants. Previous studies have shown that in chickens vaccinated with certain live attenuated vaccines, some viral subpopulations with advantageous mutations in the S protein were rapidly selected, suggesting adaptation to the chicken host [21,22,23]. These modifications in the S protein are likely responsible for the varying affinity of IBV for receptors in different tissues within the organs, thereby influencing both tropism and pathogenicity [7,23,24]. Therefore, besides the influence of the host, specific tissues among organs may apply selective pressure, favoring viral subpopulations that replicate more effectively within their particular environments [25].

As a result, we examined whether the microenvironments of different tissues representing the respiratory, renal, enteric, and reproductive systems of specific pathogen-free (SPF) laying hens influenced the subpopulation characteristics of the S1-coding sequences following infection with the IBV strain California 1737/04 (CA1737/04). This IBV strain was associated with respiratory, renal, enteric, and reproductive pathology [26]. We employed high-throughput next-generation sequencing (NGS) to sequence the S1-coding sequences across these different tissues after the infection with CA1737/04, which served as the parent isolate.

## 2. Materials and Methods

### 2.1. Ethical Approval

The utilized SPF White Leghorn hens were received from the Canadian Food Inspection Agency (CFIA) in Ottawa, Ontario. The experimental procedures and the use of laying hens in this study were conducted with ethical approval from the Veterinary Science Animal Care Committee (VSACC) at the University of Calgary under protocol number AC19-0011, approved on 19 March 2019.

### 2.2. Sample Collection

The tissue samples used in this study were obtained from a previously published study [26]. Briefly, 30-week-old SPF White Leghorn laying hens (*n* = 6) were infected with the CA1737/04 IBV strain. The infectious inoculum was derived from the allantoic fluid of the third passage of this virus, which was propagated in SPF embryonated chicken eggs. Each hen received a total of 150 µL containing 1 × 10^6^ EID_50_ of the viral strain via a combination of intratracheal, intranasal, and ocular routes. Nine days post-infection (9 dpi), tissue samples from the trachea, kidney, cecal tonsils, duodenum, colorectum, ovary, magnum, isthmus, and uterus were collected and preserved in RNA Save^®^ (Biological Industries, Beit Haemek, Israel) for RNA extraction.

### 2.3. Extraction of RNA, cDNA Synthesis, and Viral RNA Load Quantification

Total RNA from the aforementioned tissues was extracted using the Trizol^®^ reagent (Invitrogen Canada Inc., Burlington, ON, Canada), while RNA from the allantoic fluid containing the virus isolate was isolated using the Quick-RNA Viral Kit (ZymoResearch, Irvine, CA, USA), following the manufacturer’s instructions. The concentration of the obtained RNA was analyzed with a Nanodrop 1000 spectrophotometer (ThermoScientific, Wilmington, DE, USA). Complementary deoxyribonucleic acid (cDNA) was synthesized from 1 µg of the extracted RNA using random primers, following the manufacturer’s guidelines provided with the High-Capacity Reverse Transcription Kit™ (Applied Biosystems, Invitrogen Canada Inc., Burlington, ON, Canada). The viral RNA loads were quantified using real-time quantitative polymerase chain reaction (RT-qPCR) with SYBR^®^ Green Master Mix (Quantabio^®^, Beverly, MA, USA). The total reaction volume was 20 µL, consisting of 10 µL of SYBR Green master mix, 100 ng of cDNA, and 10 nmol of each of the forward and reverse primers. The assay utilized primers specific to a portion of the 5′ UTR of the IBV genome: the forward primer with the sequence 5′-GCT TTT GAG CCT AGC GTT-3′ and the reverse primer with the sequence 5′-GCC ATG TTG TCA CTG TCT ATT G-3′ [27]. The thermal cycling protocol was as follows: an initial denaturation step at 95 °C for 20 s, followed by 40 cycles of denaturation at 95 °C for 3 s and annealing and extension at 60 °C for 30 s. A melting curve analysis was performed from 65 °C to 95 °C with a 0.5 °C increment every 5 s. The viral loads were expressed as log_10_ copies and calculated using a standard curve generated from seven 10-fold serial dilutions (10^−1^ to 10^−7^) of in-house prepared plasmids containing the IBV-5′ UTR [28].

### 2.4. Amplification of Full S1, Library Preparation, and Next-Generation Sequencing

The complete S1-coding sequences were amplified using five different sets of primers to generate five individual amplicons, with overlaps ranging from 42 bp to 112 bp between the amplicons. The primers were designed based on the full S1-coding sequence of the CA1737/04 reference (accession number: OQ095389), including an additional 40 bp upstream and downstream of the complete S1, utilizing the PrimerQuest™ Tool (Integrated DNA Technologies, Coralville, IA, USA). The designed primers are presented in Table 1. Illumina overhang adapter sequences were appended to the 5′ end of each primer sequence for compatibility with the Illumina index. Each forward primer contained a forward overhang adapter, while each reverse primer included a reverse overhang adapter. The S1 amplicons were generated via reverse transcriptase PCR (RT-PCR) using recombinant Taq DNA polymerase (Invitrogen, Burlington, ON, Canada). The reaction was adjusted to a total volume of 25 µL, consisting of 2.5 µL of 10x PCR buffer, 0.75 µL of 50 mM MgCl_2_, 0.5 µL of 10 mM dNTP mix, 1 µL of each primer (10 nmol/µL), 0.1 µL of Taq DNA polymerase (5 U/µL), 4 µL of previously synthesized cDNA template (200 ng), and molecular grade water to complete the volume to 25 µL. The thermal cycling conditions were as follows: The initial denaturation was performed at 94 °C for 3 min. This was followed by 40 cycles, each consisting of denaturation at 94 °C for 45 s, annealing at 55 °C for 30 s, and extension at 72 °C for 1 min. A final extension step was carried out at 72 °C for 10 min. The expected sizes of the amplicons were confirmed using a 1.5% agarose gel. Subsequently, each of the generated amplicons was purified using the E.Z.N.A.^®^ Cycle Pure Kit (Omega Bio-tek, Norcross, GA, USA), following the manufacturer’s instructions. The purified amplicons were pooled into a single mixture and submitted to the Centre for Health Genomics and Informatics at the Cumming School of Medicine, University of Calgary, for sequencing. The libraries were prepared according to the Nextera XT (Illumina, San Diego, CA, USA) library preparation protocol. The sequencing was performed on a MiSeq Nano system with a 500-cycle kit (2 × 250 bp paired-end reads).

### 2.5. Bioinformatic Analysis of Sequencing Data

The raw sequencing data were converted to FASTQ files using bcl2fastq (Illumina). The obtained FASTQ reads were imported into CLC Genomics Workbench version 24.0.1 (CLC Bio, Qiagen, Aarhus, Denmark) and processed using a workflow plugin within the software. Initially, the reads were subjected to trimming tools to remove the adapters, primers, and short reads. Subsequently, the trimmed reads were mapped to the S1 portion of the CA1737/04 reference sequence (accession number: OQ095389) using CLC Map Reads to Reference tool. Prior to the variant calling analysis, the mapped reads were subjected to the CLC Refine Read Mapping tool to remove the mapped reads with unaligned ends, ensuring higher-quality variant detection. The variant calling was then performed on the refined mapped reads using the CLC Basic Variant Detection tool, which is based on user-defined frequency thresholds and does not apply error models or ploidy assumptions about the data. The tool identifies variants at positions where the number and frequency of the nucleotide bases differ from the reference base. The default minimum frequency filter for this tool is 35%. The variant calling output provided detailed information on the type, position, and frequency of each mutation in the tested sequences relative to the reference sequence. The type of mutation could be a single nucleotide variant (SNV), a deletion, or an insertion. SNVs are defined as alternative nucleotides that coexist with the reference nucleotide at the same genomic position within a sample, contributing to the shaping of the S1 subpopulations. Hence, these subpopulations can be described as distinct sequences of the S1, each characterized by a variable number of SNVs compared to the S1 reference sequence. The core subpopulation is defined as the S1-coding sequence containing SNVs that are predominantly detected in all individual samples of all tissue types or in at least two individual samples per tissue type. Other subpopulations share the SNVs found in the core subpopulation but include one or more additional SNVs, which are observed in at least one individual tissue sample. Additionally, the workflow output included information on coding region changes and subsequent amino acid changes. The S1-coding sequences containing the respective SNVs for each organ and individual hen were submitted to GenBank. The accession numbers for these sequences are provided in Supplementary Table S1. The S1-coding sequences from all samples, along with the parent S1 sequence, which contains the SNVs detected in the study, are included in Supplementary File S1.

### 2.6. Analysis of Genetic Variation

The genetic divergence and diversity of the S1-coding sequences, which included multiple mutations relative to the reference S1 sequence of CA1737/04, were evaluated using phylogenetic analysis and Shannon entropy, respectively.

To incorporate the SNVs into each sequence of the tissue samples, the readxl and Biostrings packages were utilized in R software (version 4.4.0). The output was a FASTA file containing the respective SNVs in each sequence, using the reference sequence as a template. The FASTA files of the S1-coding sequences containing the SNVs were aligned against the S1-coding sequence of the reference using fast Fourier transformation (MAFFT) [29] as a tool built into Geneious Prime^®^ 2023.2.1 (https://www.geneious.com/) (accessed on 29 May 2024). The best-fit evolutionary model for the phylogenetic analysis was determined using the modelTest function in the phangorn package, which evaluated multiple nucleotide substitution models based on the Akaike information criterion (AIC). The general time reversible model with invariant sites (GTR + I) was selected as the most appropriate. The phylogenetic tree was constructed using the phangorn and ape packages in R, employing the maximum likelihood (ML) method with the GTR+I model. A bootstrap analysis was performed with 1000 replicates to assess the robustness of the tree. Afterwards, the Newick format of the tree was viewed and edited using iTOL v6 [30].

The Shannon entropy was calculated to assess the genetic diversity based on the frequency distribution of the SNVs. The frequency of each SNV detected in a sample was used to compute the Shannon entropy (H) using the following formula [31]: H = − ∑p*i* log2(p*i*), where p*i* represents the proportion of each SNV within the sample. The dplyr package was used for data manipulation, and the Shannon entropy was calculated using a custom R function. The analysis was performed on an SNV frequency dataset, ensuring that the entropy values captured the diversity of the nucleotide variations across the different tissue samples.

### 2.7. Structural Modelling and Mapping of Amino Acid Mutations

Since the 3D structure of the S1 glycoprotein of CA1737/04 is not available, protein homology modeling was predicted using the I-TASSER server [32]. The boundaries of the three HVRs were determined based on previous studies [17,18], where HVR 1 is located at amino acids 38–67, HVR 2 at 91–141, and HVR 3 at 274–387. The S1-NTD and S1-CTD boundaries were defined according to the S1 protein of the M41 strain [16], with S1-NTD at amino acids 21–237 and S1-CTD at 269–414. The predicted 3D structure was visualized and edited using PyMOL software (version 2.5.7).

### 2.8. Statistical Analysis and Data Visualization

The differences in the log_10_ copies of IBV RNA loads, the number of SNVs, the Shannon entropy indices, and the total number of synonymous, nonsynonymous, and nonsense mutations among the different tissues were statistically analyzed using the Kruskal–Wallis test followed by Dunn’s multiple comparison test in GraphPad Prism 10.1.2 (GraphPad Prism Software, San Diego, CA, USA). The graphs resulting from these tests were generated using this software. To assess the correlation between the viral RNA loads (log_10_ copies) and both the Shannon entropy and the number of SNVs, we used R programming. The Pearson correlation coefficient (r) was calculated using the cor.test function, while scatter plots with regression lines were generated using the ggplot2 package. The geom_smooth (method = “lm”) function was applied to visualize the correlation, with the axis labels and formatting adjusted for clarity. The frequency of the SNVs was visualized using the pheatmap package within the R program.

## 3. Results

### 3.1. Amplification of S1

The S1-coding sequences of IBV were successfully amplified from all collected tissue samples in the cecal tonsils (6/6), colorectum (6/6), magnum (6/6), isthmus (6/6), and uterus (6/6), as well as from the allantoic fluid containing the CA1737/04 parent isolate. However, the amplification was not consistently successful in other tissues, such as the trachea (5/6), kidney (5/6), duodenum (4/6), and ovary (5/6).

### 3.2. Quality Control of Sequencing Data

Using NGS, a total of 2,493,134 reads in all tissue samples were obtained with an average of 49,863 reads per sample. Following the trimming, 2,281,373 reads remained out of the total number of raw reads, representing 91.5% (Supplementary Table S2). As shown in Table S2, the trimming process resulted in most of the reads retaining a similar length and quality. The average read length before trimming was 244.6 bp, which closely aligns with the average read length after trimming (244.1 bp). The median values (248.5 bp before trimming and 248.2 bp after trimming) and the maximum read length (249.8 bp) further indicate consistency in read length, demonstrating that trimming did not substantially alter the majority of the reads. After mapping these reads to the CA1737/04 S1 reference sequence (accession number: OQ095389), the average number of mapped reads per sample was 40,961 (Supplementary Table S3).

### 3.3. Viral RNA Loads, SNVs Occurrence, and Their Correlation

**Figure 1:**
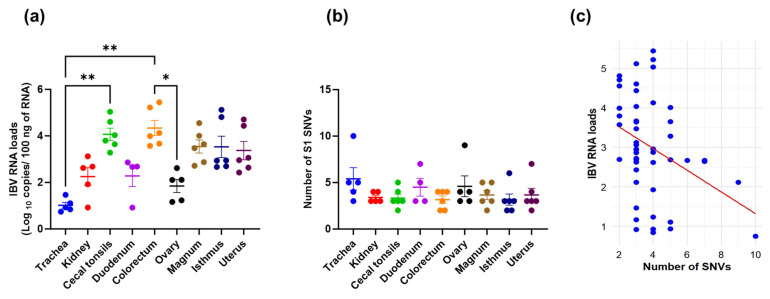
(**a**) IBV RNA loads (log_10_ copies); (**b**) Number of SNVs in various tissues. Error bars represent the standard error of the mean (SEM). Asterisks indicate significant differences (* *p* < 0.05 and ** *p* < 0.01). (**c**) Scatter plot illustrates the correlation between IBV RNA loads and the number of S1 SNVs.

The mean IBV RNA loads, measured as log_10_ copies in various tissues at 9 dpi, are presented in Figure 1a. The IBV RNA loads in the trachea were significantly lower compared to those detected in the cecal tonsils and colorectum. (*p* < 0.05). Additionally, the IBV RNA loads in the ovary were significantly lower than those observed in the colorectum (*p* < 0.05). After infection with the parent CA1737/04 strain, a varying number of SNVs with a frequency >35% were detected in the S1-coding sequences across different tissues (Figure 1b). Despite the highest number of SNVs being observed in the trachea, no statistically significant differences were observed among the examined tissues (*p* > 0.05). Conversely, the CA1737/04 parent virus, which was isolated from the allantoic fluid of the third passage and used for bird infection, showed no SNVs with a frequency above 35% in its S1-coding sequence compared to the reference S1 sequence. Generally, viral replication has been linked to the diversification of viral populations within a host. Therefore, in this study, we compared the viral RNA loads with the number of SNVs observed. The analysis revealed a significant negative correlation between the IBV RNA loads in the log_10_ copies and the number of SNVs in the S1-coding sequences (*p*-value = 0.01, Pearson correlation coefficient = −0.36; Figure 1c). The values of the log_10_ copies and the number of SNVs are presented in Supplementary Table S4.

### 3.4. S1 Subpopulations in Different Tissues

**Figure 2:**
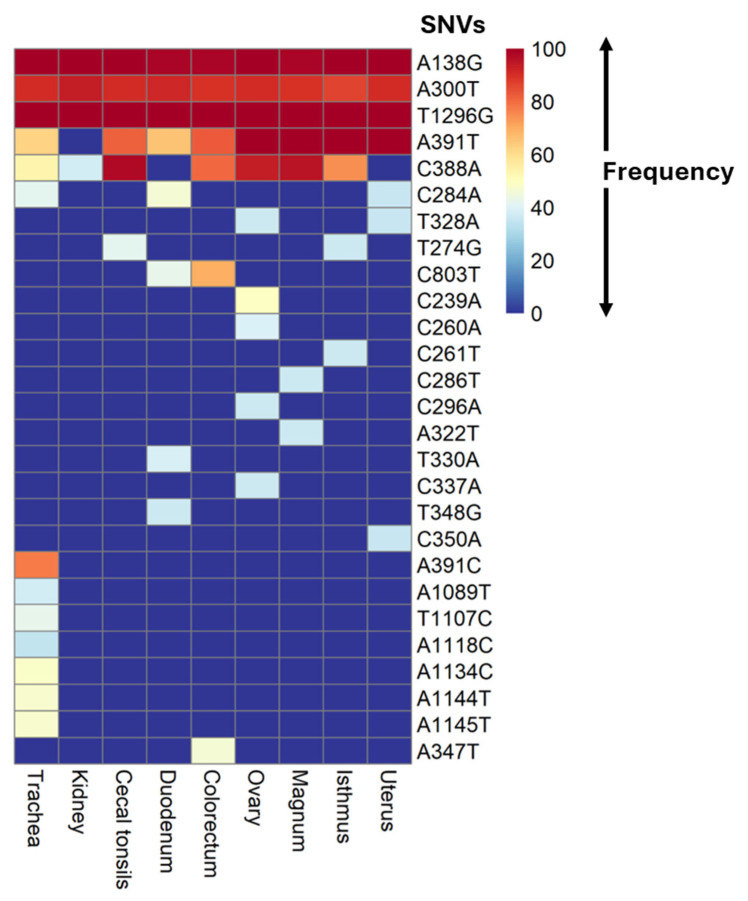
Heatmap showing the frequency of SNVs. The frequency was calculated as the number of reads supporting the nucleotide variant divided by the total number of reads covering that genomic position, expressed as a percentage. Each SNV frequency is represented as the average percentage frequency of the same SNV, calculated from all tissue samples. The SNVs were denoted as follows: the reference base, position in gene, alternate base.

A total of 27 SNVs with a frequency of >35% in individual samples from different hens and tissues were identified in the S1-coding sequences compared to the reference sequence (accession number OQ095389) (Table 2 and Table S5). These SNVs were detected across varying numbers of infected hens and tissues (Table 2 and Table S5). The frequency of the SNVs within the sequence reads of individual samples ranged from 35% to 100% (Figure 2, Supplementary Table S5).

The S1 subpopulations shaped by these SNVs were designated as SP1 to SP15 (Table 3). The distribution of the subpopulations varied across the tissues, with some found in all or most tissue types, others present in two or three tissue types, and some exclusive to a single tissue type. SP1 comprised three SNVs (A138G, A300T, and T1296G), which were prevalent across all tissue types and constituted the core subpopulation. The other subpopulations also contained the three predominant SNVs of SP1, along with additional SNVs. These additional SNVs were detected in at least one individual sample within a given tissue type. SP2 contained A138G, A300T, T1296G, A391T, and C388A, and was present in all tissues except the kidney, duodenum, and uterus. SP3 included A138G, A300T, T1296G, and C803T, and was observed in gut tissues, including the cecal tonsils, duodenum, and colorectum. Other subpopulations included SP4, found in the uterus and ovary, with SNVs A138G, A300T, T1296G, and T328A; SP5, found in the isthmus and cecal tonsils, with SNVs A138G, A300T, T1296G, and T274G; and SP6, found in the trachea, duodenum, and uterus, with SNVs A138G, A300T, T1296G, and C284A.

Other subpopulations were identified in one tissue type and were regarded as unique. SP7 and SP8, exclusive to the trachea, comprised the shared SNVs (A138G, A300T, and T1296G) and were further distinguished by the presence of A1089T, T1107C, A1118C, A1134C, A1144T, and A1145T in SP7, and A391C in SP8. SP9, restricted to the duodenum, included A138G, A300T, T1296G, and the distinct variants T330A and T348G. In the colorectum, SP10 was characterized by A138G, A300T, T1296G, and A347T. SP11, specific to the ovary, contained A138G, A300T, and T1296G, alongside C239A, C260A, C296A, and C337A. SP12 and SP13, both observed in the magnum, contained A138G, A300T, and T1296G, with C286T and A322T distinguishing them, respectively. SP14, detected in the isthmus, included A138G, A300T, T1296G, and C261T. Lastly, SP15, confined to the uterus, was distinguished by A138G, A300T, T1296G, and C350A.

### 3.5. Genetic Variation Analysis

**Figure 3:**
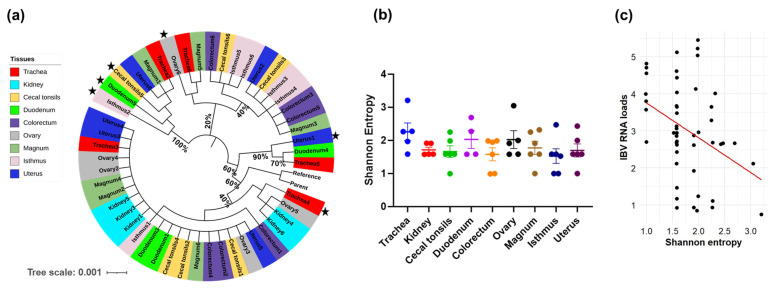
Genetic variability parameters. (**a**) Phylogenetic analysis based on nucleotide sequences of S1 involving mutants in various tissues. The tree was generated using the maximum-likelihood method and the GTR+1 model, with 1000 bootstrap replicates. The tree was viewed and edited in iTOL v6. The tip labels represent the name of the tissue and the corresponding hen number. The samples with black stars reveal phylogenetic separation. (**b**) Shannon entropy was calculated based on the frequency distribution of SNVs within S1 sequences. The entropy values across different tissues were statistically analyzed using the Kruskal–Wallis test followed by Dunn’s multiple comparison test. Error bars indicate SEM. (**c**) The scatter plot shows the correlation between the IBV RNA loads and the Shannon entropy.

The genetic divergence of the S1-coding sequences relative to the reference S1 sequence of CA1737/04 was evaluated through phylogenetic analysis, while the Shannon entropy was used to assess the genetic diversity within the sequences. The phylogenetic analysis did not reveal any clear clustering based on either the hen identity or the tissue type. The majority of the S1-coding sequences from the different tissues were closely related to each other and to the parent and reference sequences. However, a few S1-coding sequences derived from tissues of different hens displayed a genetic divergence, including trachea4, ovary6, duodenum5, cecal tonsils5, isthmus2, and uterus1 (Figure 3a, indicated with black stars at the tip labels). Notably, none of these genetically distinct sequences belonged to the same hen, except for duodenum5 and cecal tonsils5, which originated from the same hen. In terms of the Shannon entropy, the mean values in the S1-coding sequences of the trachea were the highest compared to the other tissues. (Figure 3b). However, no significant difference was detected (*p* > 0.05). Notably, the S1-coding sequences with higher entropy values corresponded to lower IBV RNA loads. Consequently, we compared the Shannon entropy with the IBV RNA loads expressed by log_10_ copies. It was noteworthy that the genetic diversity, expressed by the Shannon entropy, had a significant negative correlation with the IBV RNA loads (*p*-value = 0.009, Pearson correlation coefficient = −0.37; Figure 3c). Thus, lower viral genome loads were associated with remarkable genetic diversity.

### 3.6. Amino Acid Changes in S1 Glycoprotein

**Figure 4:**
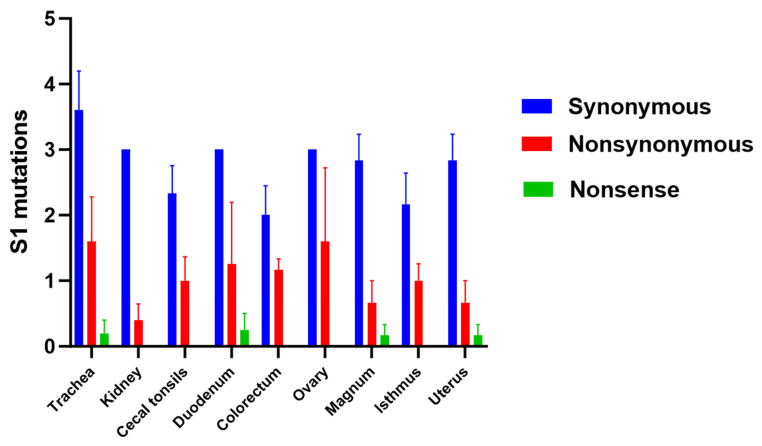
Number of synonymous, nonsynonymous, and nonsense mutations. The difference between the tissues was analyzed using the Kruskal–Wallis test followed by Dunn’s multiple comparison test. Error bars represent the SEM.

The SNVs shaping the distinct subpopulations were categorized as synonymous, nonsynonymous (resulting in different amino acids), or nonsense (resulting in stop codons) (Table 4). Although no significant differences were observed among the examined tissues regarding these types of mutations (*p* > 0.05), the highest number of synonymous and nonsynonymous mutations was observed in the trachea compared to the other tissues (Figure 4).

Compared to the S1 glycoprotein of reference CA1737/04, variable numbers of amino acid changes were observed among the subpopulations. No amino acid substitutions were observed in the core subpopulation (SP1). However, SP2, which was prevalent in all tissues except the duodenum, kidney, and uterus, contained glutamine (Q) to lysine (K) at position 130 (Q130K) and asparagine (N) to tyrosine (Y) at position 131 (N131Y). SP3, identified in different portions of the intestine, involved the threonine (T) to isoleucine (I) substitution at position 268 (T268I). In SP4, detected in both the ovary and the uterus, phenylalanine (F) was substituted with I at position 110 (F110I). The subpopulation exclusive to the cecal tonsils and isthmus, SP5, had tryptophan (W) to glycine (G) at position 92 (W92G). Replacing nucleotide C with A at position 284 introduced stop codons in the encoded S1 glycoprotein for SP6, which was detected in the trachea, uterus, and duodenum. Similarly, replacing nucleotide C with T at position 286 introduced stop codons in SP12, which was identified in the magnum. Regarding the unique subpopulations, SP7 and SP8, exclusive to the trachea, had substitutions including Y to S at position 373 (Y373S), arginine (R) to S at position 378 (R378S), K to leucine (L) at position 382 (K382L), and N to histidine (H) at position 131 (N131H). In SP9, specific to the duodenum, two substitutions were identified: F to L at position 110 (F110L) and N to K at position 116 (N116K). SP10, exclusive to the colorectum, had a single substitution: N to isoleucine (I) at position 116 (N116I). SP11, specific to the ovary, had the highest number of amino acid substitutions among the subpopulations, with four substitutions: alanine (A) to aspartic acid (D) at positions 80 (A80D) and 87 (A87D), T to N at position 99 (T99N), and histidine (H) to N at position 113 (H113N). SP12 and SP15 were associated with a single substitution each: T to S at position 108 (T108S) and A to glutamic acid (E) at position 117 (A117E), exclusively observed in the magnum and uterus, respectively. SP14, specific to the isthmus, had no amino acid substitutions.

**Figure 5:**
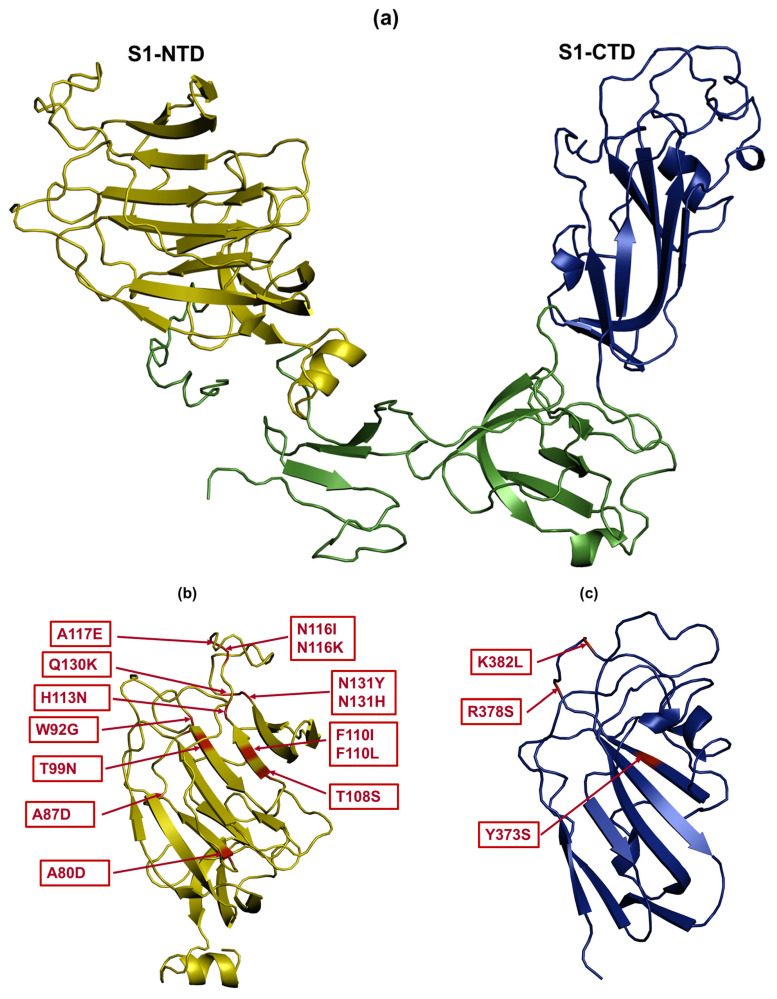
Mapping of amino acid changes in the S1 glycoprotein. (**a**) The S1 glycoprotein contains two domains: S1-NTD (indicated with yellow color) and S1-CTD (indicated with dark blue color) Amino acid substitutions are shown by red color in the S1-NTD (**b**) and the S1-CTD (**c**). PyMOL software was used to visualize the PDB files of the S1 glycoprotein.

Some substitutions may impact protein structure and stability. Charge-altering substitutions such as R378S and K382L (SP7), A80D and A87D (SP11), and A117E (SP15) could influence protein folding and interactions. Specifically, A80D and A87D (SP11) and A117E (SP15) lead to an increase in charge, while R378S and K382L (SP7) result in a decrease in charge. To evaluate the potential impact of amino acid changes in the S1 protein on viral binding to host cells, we mapped these mutations to positions within the S1-NTD and S1-CTD that have the potential to function as RBDs [16]. The S1-NTD encompasses amino acids 21–237 [16] (Figure 5a), while the S1-CTD spans amino acids 269–414 (Figure 5a) [16]. Overall, the majority of the amino acid substitutions among the subpopulations in the tissues were confined to S1-NTD (Figure 5b). Exceptions to this were observed in the trachea, where some amino acid changes (Y373S, R378S, and K382L) specific to SP7 were located in the S1-CTD (Figure 5c).

**Figure 6:**
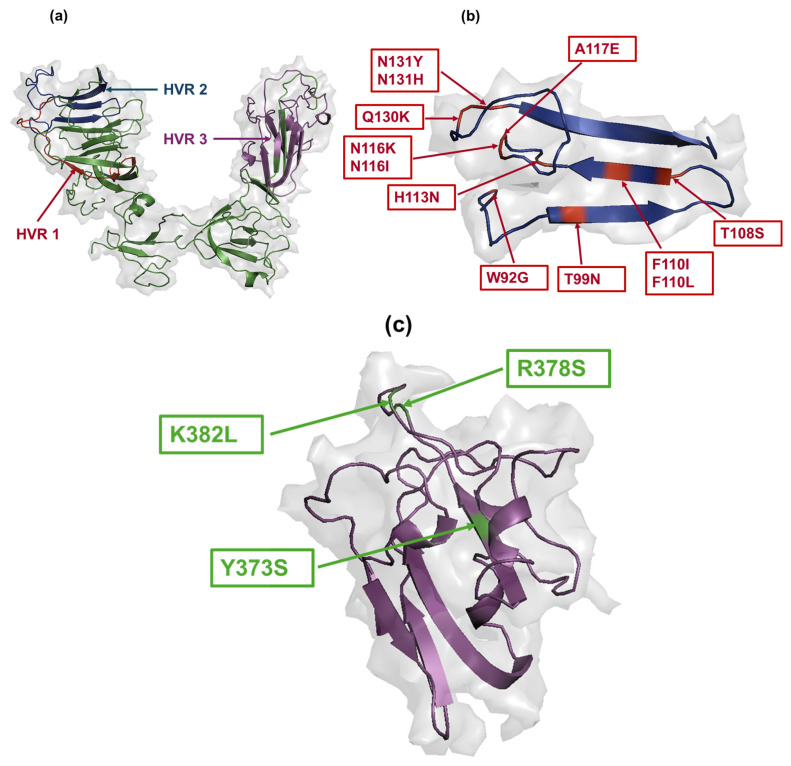
Mapping of amino acid changes in the three HVRs of the S1 glycoprotein. (**a**) HVR 1 is located at amino acid residues 38–67 (indicated with red color), HVR 2 is positioned at amino acid residues 91–141 (indicated with dark blue color), and HVR 3 is situated at amino acid residues 274–387 (indicated with magenta color). (**b**,**c**) Amino acid substitutions distributed in HVR 2 and HVR 3, respectively. The amino acid substitutions are indicated in red color in HVR 2, while they are shown in green color in the case of HVR 3. The S1 glycoprotein PDB files were visualized using PyMOL software.

In order to assess the potential of the amino acid changes included in the distinct subpopulations as immune escape variants, we mapped these substitutions within the three HVRs of the S1 protein (Figure 6a). The HVRs play crucial roles in viral infectivity, containing some neutralization sites [19] and serotype-specific epitopes [20]. The majority of the amino acid substitutions in the S1 protein across the distinct subpopulations were located in and around HVR 2 (Figure 6b). Notably, exceptions were observed in SP7, specific to the trachea, where substitutions such as Y373S, R378S, and K382L were positioned within HVR 3 (Figure 6c). No amino acid changes in any subpopulations were localized within HVR 1.

## 4. Discussion

Coronaviruses, including IBV, tend to accumulate mutations during replication, leading to the presence of a variety of subpopulations within the broader viral population [33,34]. Previous studies have demonstrated that specific tissues can exert selective pressure, influencing the gene and encoded protein of the S1 subunit, thereby affecting the selection of viral subpopulations in these particular environments [23,25]. In this study, we analyzed the S1-coding sequences derived from different tissues of laying hens infected with C1737/04 IBV to identify nucleotide (genetic) and amino acid (phenotypic) variations among these sequences. We focused on the S1-coding sequence because it is well-established that S1 contains serotype-specific epitopes [20] and epitopes that induce virus-neutralizing antibodies [19]. Furthermore, studies have shown that genetic changes frequently occur in the S1-coding sequence [35,36]. Our results have three main aspects. Firstly, nine days following infection with the CA1737/04 IBV isolate, the S1-coding sequences included variable numbers of SNVs across different tissues. These SNVs gave rise to multiple S1 subpopulations, designated as SP1 to SP15. Among these, SP1 emerged as the core subpopulation, present in all examined tissues. However, certain subpopulations were specific to two or three distinct types of tissues, while others were exclusive to a single tissue type. Secondly, the IBV RNA loads in the tissues were negatively correlated with either the number of SNVs or the genetic diversity parameter (Shannon entropy) observed in the S1-coding sequences of the respective tissues. Phylogenetic analysis further revealed a genetic divergence in the S1-coding sequences from tissue samples with lower genome loads, such as those from the trachea and ovary, compared to the parent S1 sequence. Thirdly, the SNVs within the S1 subpopulations were associated with nonsynonymous mutations. All of these amino acid changes were located in HVR 2 within the S1-NTD, except for those in SP7 (a subpopulation exclusive to the trachea), which had amino acid changes in HVR 3 within the S1-CTD. 

It has been demonstrated that the IBV genetic alterations occurring during the process of host adaptation are predominantly in the S1-coding sequences [37,38,39]. In this study, a total of 15 S1 subpopulations (SP1–SP15) were identified across different tissues of laying hens infected with the CA1737/04 IBV isolate. These subpopulations comprised 27 SNVs, which were either commonly present across multiple tissue types or specific to one, two, or three tissue types. Among these, SP1 emerged as the dominant subpopulation, characterized by the SNVs A138G, A300T, and T1296G, each detected in at least two samples per tissue type (Table 2) with a high frequency ranging from 90% to 100% (Figure 2). This suggests that SP1 is the most adaptive and fit subpopulation across all of the tissue types, potentially reflecting the similar microenvironment encountered by the virus within the different tissues. This observation aligns with the epitheliotropic nature of IBV, which targets the epithelial cells lining various organs, including the respiratory tract, alimentary tract, and urogenital tract [40]. However, these epithelial cells in various tissues differ fundamentally in certain factors, such as the nature of their secretions and their rate of turnover. Furthermore, an important and distinct factor among the tissues is the immune response, which exerts selective pressure on the invading virus. Consequently, other S1 subpopulations were selected and became specific to certain tissue types. For instance, SP3 was predominant in the gut tissues, including the duodenum, colorectum, and cecal tonsils, harboring the same three SNVs as SP1, along with an additional SNV (C803T). Other subpopulations were unique to a single tissue type; all tissues with exclusive subpopulations contained one subpopulation, except for the trachea and magnum, which each had two exclusive subpopulations.

In terms of the impact of SNVs on defining the subpopulations on the encoded S1 glycoprotein, the SNVs characterizing SP1 were synonymous, meaning they did not alter the amino acid sequence of the S1 glycoprotein. However, their dominance across all tissues suggests they may not be neutral but could have functional significance. These synonymous SNVs may influence viral fitness through mechanisms such as optimizing codon usage for efficient translation, which aligns with the findings in other coronaviruses [41]. Nevertheless, we acknowledge that the fitness of SP1 is likely influenced by genetic changes outside the S1-coding region, which were not assessed in this study. Additionally, while SP1 was dominant across the tissues, we cannot conclusively determine whether it represents a single subpopulation, as variations in other genomic regions may contribute to its fitness and prevalence. On the other hand, all tissue-specific subpopulations were associated with nonsynonymous mutations (i.e., amino acid changes), except for the isthmus. Notably, the two subpopulations specific to the trachea collectively exhibited a total of four amino acid substitutions, and the subpopulation unique to the ovary also had four substitutions. The exclusive subpopulation of the duodenum showed two substitutions, whereas the subpopulations specific to the colorectum and uterus each had a single amino acid substitution. Remarkably, the amino acid substitutions A80D and A87D in the ovary subpopulation, as well as A117E in the uterus subpopulation, resulted in an overall increase in the negative charge. In contrast, R378S and K382L in the trachea subpopulation converted residues from positively charged to uncharged (neutral), leading to a reduction in the overall charge. Changes in charge, whether an increase or a decrease, may impact the IBV spike protein’s structure, stability, and binding affinity to host receptors. Such changes warrant further investigation in IBV to elucidate their potential impact on spike protein function and viral adaptation. However, in other coronaviruses, changes in the charge of the RBD of the spike protein have been extensively studied, with several studies demonstrating that such alterations can enhance the virus’s binding affinity to the angiotensin-converting enzyme 2 (ACE2) receptor [42,43,44].

In terms of the SNVs associated with stop codons in SP6 (trachea, duodenum, and uterus) and SP12 (magnum) (Table 3 and Table 4), these mutations resulted in a truncated S1 protein in these tissues. While such truncations would typically render the virus non-infectious due to the absence of a functional S protein, they give rise to defective viral genomes (DVGs), which have been reported in coronaviruses, including IBV [45]. However, these DVGs retain viral fitness and can complement each other, enabling their persistence and contributing to viral pathogenesis, as observed in other RNA viruses [46,47]. Furthermore, the presence of stop codons in only a subset of the viral genomes within the tissues, with frequencies ranging from 35% to 46% (Table S5), supports the notion that viable IBV populations with intact S1 proteins remain dominant. These findings highlight the need for further investigation into the role of IBV defective genomes in viral evolution and adaptation, with a special emphasis on S1-coding sequence defects and their impact on viral fitness, receptor binding, and host tropism.

On the other hand, the parental CA1737/04 strain, obtained from the allantoic fluid of the third passage in embryonated chicken eggs, exhibited no SNVs in its S1-coding sequence when compared to that of the reference sequence prior to its inoculation in hens. However, the adaptation of IBV to Vero cells or its attenuation through serial passages in embryonated eggs often leads to a higher accumulation of changes in the S1-coding sequences [48,49]. One possible explanation for this discrepancy could be the use of the current bioinformatics pipeline with a high-frequency cutoff for calling variants.

Based on the aforementioned findings, it can be suggested that the detected SNVs in the tissues arose within the host following the challenge rather than pre-existing in the inoculum. However, it remains possible that some SNVs were present at extremely low frequencies in the inoculum and subsequently expanded due to the selection pressures in specific tissues. This observation is consistent with the findings of McKinley et al. [21] who reported that certain viral subpopulations that were not predominant in the original IBV vaccine underwent selection and became more prevalent in the chickens after vaccination. In addition, the core subpopulation (SP1), encompassing the three SNVs (A138G, A300T, T1296G), was distributed across all tissue types and most hens (Table 2), suggesting that this subpopulation was positively selected and became dominant throughout the infection process. This may play a crucial role in the replication of this IBV strain, regardless of tissue type; however, further investigations are needed to confirm this. On the other hand, other subpopulations were restricted to specific tissues, potentially reflecting tissue-specific selective pressures. This aligns with a previous study showing that certain IBV populations were significantly more prevalent in specific tissues than others [25]. However, the vast majority of SNVs shaping the tissue-specific subpopulations were detected in a single tissue sample and, consequently, in only one hen. This suggests that the selection may be hen-specific. Nevertheless, if a larger number of hens had been included, it is possible that these exclusive subpopulations would have been observed in more than one individual tissue sample.

With regard to the correlation between IBV RNA loads and SNVs or the Shannon entropy, a negative correlation was observed. Specifically, the tissues with viral RNA loads, such as the trachea and ovary, included samples with higher numbers of SNVs and elevated Shannon entropy values. For example, trachea4 exhibited 10 SNVs and a Shannon entropy of approximately 3.2, while ovary6 displayed 9 SNVs with a Shannon entropy of around 3.05 (Table S4). Additionally, phylogenetic analysis revealed that the S1-coding sequences of these two samples had a divergence from the S1-coding sequence of the parent or reference. Nevertheless, in tissue types that generally exhibited higher genome loads, such as the uterus, cecal tonsils, and isthmus, some individual samples, notably uterus1, cecal tonsils5, and isthmus2, harbored higher numbers of SNVs and elevated Shannon entropy values. Remarkably, these individual samples had lower IBV RNA loads compared to other counterparts, thus maintaining the overall negative correlation between viral RNA loads and the Shannon entropy or SNVs throughout all of the tissue types. However, this pattern did not hold universally; for example, duodenum5 had a higher number of SNVs and a greater Shannon entropy value but was not the lowest in terms of IBV RNA load among other duodenum samples. The explanation for the negative relationship between the IBV RNA loads and the genetic variability of the S1-coding sequences is not well-established in the existing literature, particularly for coronaviruses and other RNA viruses. Therefore, further studies are warranted to explore the nature of this correlation and to determine whether it is consistently observed across different viral infections and host environments.

The S1 glycoprotein of IBV performs crucial functions, including mediating the virus’s binding to host cell receptors [7]. As a result of this function, some epitopes within the S1 protein can be neutralizing [19] or serotype-specific [20]. The S1 glycoprotein contains three HVRs: HVR 1 and HVR 2 in the NTD, and HVR 3 in the CTD [50,51]. It has been demonstrated that both S1-NTD and S1-CTD can function as RBDs for IBV [16]. The HVRs, which contain immune-related epitopes, including neutralization sites and serotype-specific epitopes [19,20], are key regions for hypothesizing whether the S1 subpopulations with amino acid substitutions in these regions may contribute to immune evasion. In this study, the vast majority of the amino acid substitutions detected in the S1 protein sequences of subpopulations from all tissues were located in the S1-NTD, particularly in HVR 2. However, the trachea involved an additional unique feature, with subpopulations containing substitutions not only in HVR 2 but also in S1-CTD, covering the area of HVR 3. Overall, the amino acid changes identified in this study may influence IBV binding to host cell receptors or potentially give rise to new serotypes or immune-evading subpopulations. However, previous research suggests that mutations within HVRs alone may not be sufficient to drive serotypic changes [52,53], as other neutralizing epitopes in the S1 or S2 subunit could provide compensatory effects [54,55].

While this study contributes valuable insights into IBV evolution, it is not without limitations. One notable limitation lies in the bioinformatic approach used, which was designed to identify SNVs with a frequency threshold of 35%, thereby excluding variants below this cutoff. However, in deep sequencing studies, low-frequency SNVs, including those present in fewer than 1% of the reads, may hold significant importance [56]. For example, low-frequency variants within a viral population in a single host can play a critical role in the development of drug resistance and the likelihood of treatment failure [57,58]. The rationale for employing a high-frequency cutoff was to minimize the impact of the sequence errors introduced during the PCR amplification using the current polymerase enzyme and the sequencing process. An additional limitation of this study is that the current pipeline identified SNVs in paired-end reads (2 × 250 bp) derived from five different PCR amplicons without confirming whether they belong to the same full-length S1 contig. This limitation could have been addressed by incorporating de novo assembly or using a long-read sequencing approach. However, de novo assembly of short reads remains challenging [59]. Nevertheless, the analytical pipeline employed in this study remains well-suited to our aim, as it focuses on studying SNVs at the broader tissue level rather than at the individual sample level. Additionally, it is a robust approach, incorporating stringent quality control measures such as the CLC Refine Read Mapping tool, which minimizes the risk of false-positive variant calls by removing mapped reads with unaligned ends. Another limitation of this study was the inability to investigate the dynamics of the S1 subpopulations at stages of IBV infection earlier or later than the predetermined time point (9 dpi). Three to five days following IBV infection would have provided valuable information, particularly in tissues of entry such as the trachea, where IBV RNA loads are known to peak during this period [60]. An extended period after IBV infection could have offered important observations into organs where IBV persists for weeks, such as the cecal tonsils [61]. However, the current euthanasia time point (9 dpi) or a time point in close proximity, such as 10 dpi, is optimal for studying other tissues, particularly the oviduct portions (magnum, isthmus, and uterus), where IBV establishes infection with significantly higher viral RNA loads and histopathological lesions at these stages [62].

## 5. Conclusions

This study provides insights into the evolutionary dynamics of IBV by examining the subpopulations in its S1-coding sequence. The current findings revealed that, at nine days following infection with the CA1737/04 IBV isolate, the S1-coding sequences exhibited variable SNVs across different tissues. These SNVs gave rise to distinct subpopulations, with some being tissue-specific. The IBV RNA loads were negatively correlated with the number of SNVs or the Shannon entropy, and phylogenetic analysis indicated that the S1-coding sequences from tissues with lower IBV RNA loads, such as the trachea and ovary, had a genetic divergence from the parent S1 sequence. Additionally, the SNVs were linked to nonsynonymous mutations, primarily in HVR 2 of the S1-NTD, with some exceptions, such as changes in HVR 3 of the S1-NTD in trachea-specific sequences.

## Figures and Tables

**Table 1:** Primers used to amplify the full S1-coding sequences.

Primer Name	Primer Sequence (5′-3′) ^a^	Amplicon Size
Forward primer 1 Reverse primer 1	**TCGTCGGCAGCGTCAGATGTGTATAAGAGACAG**GTGTGGTAAGTTGCTGGTAAGA **GTCTCGTGGGCTCGGAGATGTGTATAAGAGACAG**TGCGAATATGATTCTGTGGTACA	426 bp
Forward primer 2 Reverse primer 2	**TCGTCGGCAGCGTCAGATGTGTATAAGAGACAG**CCTGCCCTACAACAGGTTT **GTCTCGTGGGCTC GGAGATGTGTATAAGAGACAG**CTCCCGTAAACAATGAACTTCTG	425 bp
Forward primer 3 Reverse primer 3	**TCGTCGGCAGCGTCAGATGTGTATAAGAGACAG**ACACCTAGAGGTTTATTAGCATGTC **GTCTCGTGGGCTCGGAGATGTGTATAAGAGACAG**CTGCTTACACCCACCTTGAA	411 bp
Forward primer 4 Reverse primer 4	**TCGTCGGCAGCGTCAGATGTGTATAAGAGACAG**TGGCTTGTGGTTTAATTCACTTTC **GTCTCGTGGGCTCGGAGATGTGTATAAGAGACAG**TGTCTATAGCACCTGACGTATCT	407 bp
Forward primer 5 Reverse primer 5	**TCGTCGGCAGCGTCAGATGTGTATAAGAGACAG**TATGGCAGAGTTGGACAAGG **GTCTCGTGGGCTCGGAGATGTGTATAAGAGACAG**AACTAACATATGGACAATTAGTAACATT	343 bp

^a^ Sequences in bold characters refer to the Illumina overhang adapters.

**Table 2:** Distribution of SNVs across various tissues collected from infected hens at 9 dpi.

Tissue									
SNVs	Trachea	Kidney	Cecal Tonsils	Duodenum	Colorectum	Ovary	Magnum	Isthmus	Uterus
A138G	5/5 ^a^	5/5	5/6	4/4	3/6	5/5	5/6	2/6	5/6
A300T	5/5	5/5	4/6	4/4	3/6	5/5	5/6	2/6	5/6
T1296G	5/5	5/5	6/6	4/4	6/6	5/5	6/6	6/6	6/6
A391T	2/5	0/5	3/6	2/4	3/6	1/5	3/6	5/6	2/6
C388A	2/5	2/5	1/6	0/4	1/6	2/5	1/6	2/6	0/6
C803T	0/5	0/5	1/6	1/4	2/6	0/5	0/6	0/6	0/6
T328A	0/5	0/5	0/6	0/4	0/6	1/5	0/6	0/6	1/6
T274G	0/5	0/5	1/6	0/4	0/6	0/5	0/6	1/6	0/6
C284A	1/5	0/5	0/6	1/4	0/6	0/5	0/6	0/6	1/6
A1089T	1/5	0/5	0/6	0/4	0/6	0/5	0/6	0/6	0/6
T1107C	1/5	0/5	0/6	0/4	0/6	0/5	0/6	0/6	0/6
A1118C	1/5	0/5	0/6	0/4	0/6	0/5	0/6	0/6	0/6
A1134C	1/5	0/5	0/6	0/4	0/6	0/5	0/6	0/6	0/6
A1144T	1/5	0/5	0/6	0/4	0/6	0/5	0/6	0/6	0/6
A1145T	1/5	0/5	0/6	0/4	0/6	0/5	0/6	0/6	0/6
A391C	1/5	0/5	0/6	0/4	0/6	0/5	0/6	0/6	0/6
T330A	0/5	0/5	0/5	1/4	0/6	0/5	0/6	0/6	0/6
T348G	0/5	0/5	0/5	1/4	0/6	0/5	0/6	0/6	0/6
A347T	0/5	0/5	0/5	0/4	1/6	0/5	0/6	0/6	0/6
C260A	0/5	0/5	0/5	0/4	0/6	1/5	0/6	0/6	0/6
C239A	0/5	0/5	0/5	0/4	0/6	1/5	0/6	0/6	0/6
C296A	0/5	0/5	0/5	0/4	0/6	1/5	0/6	0/6	0/6
C337A	0/5	0/5	0/5	0/4	0/6	1/5	0/6	0/6	0/6
C286T	0/5	0/5	0/5	0/4	0/6	0/5	1/6	0/6	0/6
A322T	0/5	0/5	0/5	0/4	0/6	0/5	1/6	0/6	0/6
C261T	0/5	0/5	0/5	0/4	0/6	0/5	0/6	1/6	0/6
C350A	0/5	0/5	0/5	0/4	0/6	0/5	0/6	0/6	1/6

^a^ The number of tissue samples of the same type harboring SNVs out of the total number of tissue samples collected from infected hens for which S1-coding sequences were determined (*n* = 4–6).

**Table 3:** The S1 subpopulations detected in different tissues.

Designation	SNVs	Tissue
SP1	A138G, A300T, T1296G	All tissues
SP2	A138G, A300T, T1296G, ^a^ **A391T**, **C388A**	All tissues, except kidney, duodenum and uterus
SP3	A138G, A300T, T1296G, **C803T**	Cecal tonsils, Duodenum, colorectum
SP4	A138G, A300T, T1296G, **T328A**	Uterus, ovary
SP5	A138G, A300T, T1296G, **T274G**	Isthmus, cecal tonsils
SP6	A138G, A300T, T1296G, **C284A**	Trachea, uterus, duodenum
SP7	A138G, A300T, T1296G, **A1089T**, **T1107C**, **A1118C**, **A1134C**, **A1144T**, **A1145T**	Trachea
SP8	A138G, A300T, T1296G, **A391C**	Trachea
SP9	A138G, A300T, T1296G, **T330A**, **T348G**	Duodenum
SP10	A138G, A300T, T1296G, **A347T**	Colorectum
SP11	A138G, A300T, T1296G, **C239A**, **C260A**, **C296A**, **C337A**	Ovary
SP12	A138G, A300T, T1296G, **C286T**	Magnum
SP13	A138G, A300T, T1296G, **A322T**	Magnum
SP14	A138G, A300T, T1296G, **C261T**	Isthmus
SP15	A138G, A300T, T1296G, **C350A**	Uterus

^a^ The additional SNVs beyond the core SNVs are denoted in bold and underlined.

**Table 4:** Classification of SNVs detected in each subpopulation based on their effect on the S1 glycoprotein.

Subpopulation	Synonymous	Nonsense	Nonsynonymous
SP1	A138G, A300T, T1296G (Core SNVs)	-	- ^a^
SP2	Core SNVs	-	C388A (Q130K) ^b^ A391T (N131Y)
SP3	Core SNVs	-	C803T (T268I)
SP4	Core SNVs	-	T328A (F110I)
SP5	Core SNVs	-	T274G (W92G)
SP6	Core SNVs	C284A (S95 *)	-
SP7	Core SNVs, A1089T, T1107C	-	A1118C (Y373S) A1134C (R378S) A1144T, A1145T (K382L)
SP8	Core SNVs	-	A391C (N131H)
SP9	Core SNVs	-	T330A (F110L) T348G (N116K)
SP10	Core SNVs	-	A347T (N116I)
SP11	Core SNVs	-	C239A (A80D) C260A (A87D) C296A (T99N) C337A (H113N)
SP12	Core SNVs	C286T (Q96 *)	-
SP13	Core SNVs	-	A322T (T108S)
SP14	Core SNVs, C261T	-	-
SP15	Core SNVs	-	C350A (A117E)

^a^ No SNVs detected in this category. ^b^ The amino acid substitution corresponding to the identified SNV is presented as follows: the amino acid in the reference S1 glycoprotein, its position, and the substituted amino acid in the S1 glycoprotein of the respective subpopulation. * Refers to stop codons.

## Data Availability

The datasets used and/or analyzed within the frame of the study can be provided by the corresponding author upon reasonable request.

## Supplementary Materials

The following supporting information can be downloaded at: https://www.mdpi.com/article/10.3390/v17040527/s1, Table S1: shows the accession numbers of complete S1 gene sequences derived from the examined tissues and allantoic fluid; Table S2: reveals the raw reads before and after trimming; Table S3: indicates the mapped reads against the reference sequence; Table S4: shows the values of log10 copies, the number of SNVs, and the Shannon entropy values; Table S5: reveals the occurrence and frequency of SNVs in each individual tissue sample; File S1: shows the S1-coding sequences derived from all of the examined samples.
