# Supplementary material for: Genetic and Phenotypic Investigations of Viral Subpopulations Detected in Different Tissues of Laying Hens Following Infectious Bronchitis Virus Infection
Source: Viruses. 2025 Apr 4;17(4):527. doi: 10.3390/v17040527 (PMC12030972; doi:10.3390/v17040527)
Supplement: Supplementary file 1 [file viruses-17-00527-s001.zip › viruses-3403368-File S1.pdf]

>Parent

ATGTCGGTAACACCTCTTTTATTAGTGACTCTTTTGTTTGCACTATGTAGTGCTGCTTTGTATGACAAA
GGTTCTTATGTTTACTACTACCAAAGTGCCTTTAGACCACCAGATGGTTGGCATTTACAAGGAGGT
GCATATGCAGTAGTTAATTCTACTAATTACTCTAATAATGCAGGCGACGCAGCACTATGTACTGGTG
GTTTGCTTACAGATGTTTACAACAACACAGCTGCTGCTATATCTATGGTAGCACCGGCCTCAGGTA
TGAGTTGGTCTACGTCACAGTTTTGTACTGCACATTGTAGATTCTCAGACCTTACTGTGTTTGTTACG
CATTGTTATAATGCGTCTACGGGTGCCTGCCCTACAACAGGTTTTGTACCACAGAATCATATTCGC
ATTTCTGCTATGAGAAATGGTTCTTTTCTTTATAACTCAACATTTAGTGTGGTTAAACATCCTAAGTTTT
ATTCTTTTCAATGTGTTAACAACCAAACATCTGTGTATCTTAATGGTGATCTTGTTTACACTTCCAACA
TCACCACTGATGTTACGTCAGCAGGTGTGCATTTTAAAGCAGGTGGACCTGTAAATTATAATGTTAT
GAAAGAATTTCAGGTACTTGCTTATTTTGTTAATGGAACTGTACAAGACGTTATCTTGTGCGATGAAA
CACCTAGAGGTTTATTAGCATGTCAATATAATACTGGCAATTTTTCAGATGGATTTTACCCTTTTACTA
ATAATACTTTAGTAAAACAGAAGTTCATTGTTTATCGGGAGAATAGTGTTAATACTACTTTGGTTTTGC
ATAATGTTACTTTTAGTAATGAGACTAATGCACAACCTAATATAGGTGGTGTTGATAATATTAATTTATA
CCAAACATATACAGCTCAGAGTGGTTATTATAATTTTAATTTTTCCTTTCTGAGTGGTTTTGTCTATAAG
GAGTCTGATTTTATGTATGGATCTTATCACCCAAGTTGTAAGTTTAGACCAGAAACTATTAATAATGG
CTTGTGGTTTAATTCACTTTCAATTTCACTTGCATATGGCCCCCTTCAAGGTGGGTGTAAGCAGTC
AGTTTTTAGTCGTAGGGCTACTTGTTGTTATGCCTACTCTTATAGAGGACCACATAAGTGTAAAGGA
GTTTATAGTGGTGAGTTATTAAAAGATTTTGAATGTGGGCTGTTGGTTTATGTTACTAAGAGTGATGG
CTCTCGCATACAAACAGCCACAGAATCACCAGTTATAACTCAACACAATTATAATAATATTACTTTA
AATACTTGTGTTGAGTATAATATATATGGCAGAGTTGGACAAGGTTTTATTACTAATGTAACTGACTCA
GCATCTATGGGGAATTATTTAGCAGATGCAGGACTAGCTATTTTAGATACGTCAGGTGCTATAGACA
CCTTTGTTGTACAAGGTGGATATGGTCTCAATTATTATAAGGTTAACCCGTGTGAAGATGTTAACCA
GCAGTTTGTAGTGTCAGGCGGTAAGTTAGTAGGCATTCTGACTTCTCGTAATGAAACTGATTCTTAT
CCTCTTGAAAATCAGTTTTATATTAAGTTAACTAATGGAAGCCGTCGTTCTAGACGT

>Trachea2

ATGTCGGTAACACCTCTTTTATTAGTGACTCTTTTGTTTGCACTATGTAGTGCTGCTTTGTATGACAAA
GGTTCTTATGTTTACTACTACCAAAGTGCCTTTAGACCACCAGATGGTTGGCATTTACAAGGAGGT
GCGTATGCAGTAGTTAATTCTACTAATTACTCTAATAATGCAGGCGACGCAGCACTATGTACTGGTG
GTTTGCTTACAGATGTTTACAACAACACAGCTGCTGCTATATCTATGGTAGCACCGGCCTCAGGTA
TGAGTTGGTCTACGTCACAGTTTTGTACTGCTCATTGTAGATTCTCAGACCTTACTGTGTTTGTTACG
CATTGTTATAATGCGTCTACGGGTGCCTGCCCTACAACAGGTTTTGTACCACAGTATCATATTCGC
ATTTCTGCTATGAGAAATGGTTCTTTTCTTTATAACTCAACATTTAGTGTGGTTAAACATCCTAAGTTTT
ATTCTTTTCAATGTGTTAACAACCAAACATCTGTGTATCTTAATGGTGATCTTGTTTACACTTCCAACA
TCACCACTGATGTTACGTCAGCAGGTGTGCATTTTAAAGCAGGTGGACCTGTAAATTATAATGTTAT

GAAAGAATTTCAGGTACTTGCTTATTTTGTTAATGGAACTGTACAAGACGTTATCTTGTGCGATGAAA
CACCTAGAGGTTTATTAGCATGTCAATATAATACTGGCAATTTTTCAGATGGATTTTACCCTTTTACTA
ATAATACTTTAGTAAAACAGAAGTTCATTGTTTATCGGGAGAATAGTGTTAATACTACTTTGGTTTTGC
ATAATGTTACTTTTAGTAATGAGACTAATGCACAACCTAATATAGGTGGTGTTGATAATATTAATTTATA
CCAAACATATACAGCTCAGAGTGGTTATTATAATTTTAATTTTTCCTTTCTGAGTGGTTTTGTCTATAAG
GAGTCTGATTTTATGTATGGATCTTATCACCCAAGTTGTAAGTTTAGACCAGAAACTATTAATAATGG
CTTGTGGTTTAATTCACTTTCAATTTCACTTGCATATGGCCCCCTTCAAGGTGGGTGTAAGCAGTC
AGTTTTTAGTCGTAGGGCTACTTGTTGTTATGCCTACTCTTATAGAGGACCACATAAGTGTAAAGGA
GTTTATAGTGGTGAGTTATTAAAAGATTTTGAATGTGGGCTGTTGGTTTATGTTACTAAGAGTGATGG
CTCTCGCATACAAACAGCCACAGAATCACCAGTTATAACTCAACACAATTATAATAATATTACTTTA
AATACGTGTGTTGAGTATAATATATATGGCAGAGTTGGACAAGGTTTTATTACTAATGTAACTGACTCA
GCATCTATGGGGAATTATTTAGCAGATGCAGGACTAGCTATTTTAGATACGTCAGGTGCTATAGACA
CCTTTGTTGTACAAGGTGGATATGGTCTCAATTATTATAAGGTTAACCCGTGTGAAGATGTTAACCA
GCAGTTTGTAGTGTCAGGCGGTAAGTTAGTAGGCATTCTGACTTCTCGTAATGAAACTGATTCTTAT
CCTCTTGAAAATCAGTTTTATATTAAGTTAACTAATGGAAGCCGTCGTTCTAGACGT

>Trachea3

ATGTCGGTAACACCTCTTTTATTAGTGACTCTTTTGTTTGCACTATGTAGTGCTGCTTTGTATGACAAA
GGTTCTTATGTTTACTACTACCAAAGTGCCTTTAGACCACCAGATGGTTGGCATTTACAAGGAGGT
GCGTATGCAGTAGTTAATTCTACTAATTACTCTAATAATGCAGGCGACGCAGCACTATGTACTGGTG
GTTTGCTTACAGATGTTTACAACAACACAGCTGCTGCTATATCTATGGTAGCACCGGCCTCAGGTA
TGAGTTGGTCTACGTCACAGTTTTGTACTGCTCATTGTAGATTCTCAGACCTTACTGTGTTTGTTACG
CATTGTTATAATGCGTCTACGGGTGCCTGCCCTACAACAGGTTTTGTACCACAGAATCATATTCGC
ATTTCTGCTATGAGAAATGGTTCTTTTCTTTATAACTCAACATTTAGTGTGGTTAAACATCCTAAGTTTT
ATTCTTTTCAATGTGTTAACAACCAAACATCTGTGTATCTTAATGGTGATCTTGTTTACACTTCCAACA
TCACCACTGATGTTACGTCAGCAGGTGTGCATTTTAAAGCAGGTGGACCTGTAAATTATAATGTTAT
GAAAGAATTTCAGGTACTTGCTTATTTTGTTAATGGAACTGTACAAGACGTTATCTTGTGCGATGAAA
CACCTAGAGGTTTATTAGCATGTCAATATAATACTGGCAATTTTTCAGATGGATTTTACCCTTTTACTA
ATAATACTTTAGTAAAACAGAAGTTCATTGTTTATCGGGAGAATAGTGTTAATACTACTTTGGTTTTGC
ATAATGTTACTTTTAGTAATGAGACTAATGCACAACCTAATATAGGTGGTGTTGATAATATTAATTTATA
CCAAACATATACAGCTCAGAGTGGTTATTATAATTTTAATTTTTCCTTTCTGAGTGGTTTTGTCTATAAG
GAGTCTGATTTTATGTATGGATCTTATCACCCAAGTTGTAAGTTTAGACCAGAAACTATTAATAATGG
CTTGTGGTTTAATTCACTTTCAATTTCACTTGCATATGGCCCCCTTCAAGGTGGGTGTAAGCAGTC
AGTTTTTAGTCGTAGGGCTACTTGTTGTTATGCCTACTCTTATAGAGGACCACATAAGTGTAAAGGA
GTTTATAGTGGTGAGTTATTAAAAGATTTTGAATGTGGGCTGTTGGTTTATGTTACTAAGAGTGATGG
CTCTCGCATACAAACAGCCACAGAATCACCAGTTATAACTCAACACAATTATAATAATATTACTTTA

AATACGTGTGTTGAGTATAATATATATGGCAGAGTTGGACAAGGTTTTATTACTAATGTAACTGACTCA
GCATCTATGGGGAATTATTTAGCAGATGCAGGACTAGCTATTTTAGATACGTCAGGTGCTATAGACA
CCTTTGTTGTACAAGGTGGATATGGTCTCAATTATTATAAGGTTAACCCGTGTGAAGATGTTAACCA
GCAGTTTGTAGTGTCAGGCGGTAAGTTAGTAGGCATTCTGACTTCTCGTAATGAAACTGATTCTTAT
CCTCTTGAAAATCAGTTTTATATTAAGTTAACTAATGGAAGCCGTCGTTCTAGACGT

>Trachea4

ATGTCGGTAACACCTCTTTTATTAGTGACTCTTTTGTTTGCACTATGTAGTGCTGCTTTGTATGACAAA
GGTTCTTATGTTTACTACTACCAAAGTGCCTTTAGACCACCAGATGGTTGGCATTTACAAGGAGGT
GCGTATGCAGTAGTTAATTCTACTAATTACTCTAATAATGCAGGCGACGCAGCACTATGTACTGGTG
GTTTGCTTACAGATGTTTACAACAACACAGCTGCTGCTATATCTATGGTAGCACCGGCCTCAGGTA
TGAGTTGGTCTACGTCACAGTTTTGTACTGCTCATTGTAGATTCTCAGACCTTACTGTGTTTGTTACG
CATTGTTATAATGCGTCTACGGGTGCCTGCCCTACAACAGGTTTTGTACCAAAGAATCATATTCGC
ATTTCTGCTATGAGAAATGGTTCTTTTCTTTATAACTCAACATTTAGTGTGGTTAAACATCCTAAGTTTT
ATTCTTTTCAATGTGTTAACAACCAAACATCTGTGTATCTTAATGGTGATCTTGTTTACACTTCCAACA
TCACCACTGATGTTACGTCAGCAGGTGTGCATTTTAAAGCAGGTGGACCTGTAAATTATAATGTTAT
GAAAGAATTTCAGGTACTTGCTTATTTTGTTAATGGAACTGTACAAGACGTTATCTTGTGCGATGAAA
CACCTAGAGGTTTATTAGCATGTCAATATAATACTGGCAATTTTTCAGATGGATTTTACCCTTTTACTA
ATAATACTTTAGTAAAACAGAAGTTCATTGTTTATCGGGAGAATAGTGTTAATACTACTTTGGTTTTGC
ATAATGTTACTTTTAGTAATGAGACTAATGCACAACCTAATATAGGTGGTGTTGATAATATTAATTTATA
CCAAACATATACAGCTCAGAGTGGTTATTATAATTTTAATTTTTCCTTTCTGAGTGGTTTTGTCTATAAG
GAGTCTGATTTTATGTATGGATCTTATCACCCAAGTTGTAAGTTTAGACCAGAAACTATTAATAATGG
CTTGTGGTTTAATTCACTTTCAATTTCACTTGCATATGGCCCCCTTCAAGGTGGGTGTAAGCAGTCT
GTTTTTAGTCGTAGGGCCACTTGTTGTTCTGCCTACTCTTATAGCGGACCACATTTGTGTAAAGGA
GTTTATAGTGGTGAGTTATTAAAAGATTTTGAATGTGGGCTGTTGGTTTATGTTACTAAGAGTGATGG
CTCTCGCATACAAACAGCCACAGAATCACCAGTTATAACTCAACACAATTATAATAATATTACTTTA
AATACGTGTGTTGAGTATAATATATATGGCAGAGTTGGACAAGGTTTTATTACTAATGTAACTGACTCA
GCATCTATGGGGAATTATTTAGCAGATGCAGGACTAGCTATTTTAGATACGTCAGGTGCTATAGACA
CCTTTGTTGTACAAGGTGGATATGGTCTCAATTATTATAAGGTTAACCCGTGTGAAGATGTTAACCA
GCAGTTTGTAGTGTCAGGCGGTAAGTTAGTAGGCATTCTGACTTCTCGTAATGAAACTGATTCTTAT
CCTCTTGAAAATCAGTTTTATATTAAGTTAACTAATGGAAGCCGTCGTTCTAGACGT

>Trachea5

ATGTCGGTAACACCTCTTTTATTAGTGACTCTTTTGTTTGCACTATGTAGTGCTGCTTTGTATGACAAA
GGTTCTTATGTTTACTACTACCAAAGTGCCTTTAGACCACCAGATGGTTGGCATTTACAAGGAGGT

GCGTATGCAGTAGTTAATTCTACTAATTACTCTAATAATGCAGGCGACGCAGCACTATGTACTGGTG
GTTTGCTTACAGATGTTTACAACAACACAGCTGCTGCTATATCTATGGTAGCACCGGCCTCAGGTA
TGAGTTGGTCTACGTAACAGTTTTGTACTGCTCATTGTAGATTCTCAGACCTTACTGTGTTTGTTACG
CATTGTTATAATGCGTCTACGGGTGCCTGCCCTACAACAGGTTTTGTACCACAGCATCATATTCGC
ATTTCTGCTATGAGAAATGGTTCTTTTCTTTATAACTCAACATTTAGTGTGGTTAAACATCCTAAGTTTT
ATTCTTTTCAATGTGTTAACAACCAAACATCTGTGTATCTTAATGGTGATCTTGTTTACACTTCCAACA
TCACCACTGATGTTACGTCAGCAGGTGTGCATTTTAAAGCAGGTGGACCTGTAAATTATAATGTTAT
GAAAGAATTTCAGGTACTTGCTTATTTTGTTAATGGAACTGTACAAGACGTTATCTTGTGCGATGAAA
CACCTAGAGGTTTATTAGCATGTCAATATAATACTGGCAATTTTTCAGATGGATTTACCCTTTTACTA
ATAATACTTTAGTAAAACAGAAGTTCATTGTTTATCGGGAGAATAGTGTTAATACTACTTTGGTTTTGC
ATAATGTTACTTTTAGTAATGAGACTAATGCACAACCTAATATAGGTGGTGTTGATAATATTAATTTATA
CCAAACATATACAGCTCAGAGTGGTTATTATAATTTTAATTTTTCCTTTCTGAGTGGTTTTGTCTATAAG
GAGTCTGATTTTATGTATGGATCTTATCACCCAAGTTGTAAGTTTAGACCAGAAACTATTAATAATGG
CTTGTGGTTTAATTCACTTTCAATTTCACTTGCATATGGCCCCCTTCAAGGTGGGTGTAAGCAGTC
AGTTTTTAGTCGTAGGGCTACTTGTTGTTATGCCTACTCTTATAGAGGACCACATAAGTGTAAAGGA
GTTTATAGTGGTGAGTTATTAAAAGATTTTGAATGTGGGCTGTTGGTTTATGTTACTAAGAGTGATGG
CTCTCGCATACAAACAGCCACAGAATCACCAGTTATAACTCAACACAATTATAATAATATTACTTTA
AATACGTGTGTTGAGTATAATATATATGGCAGAGTTGGACAAGGTTTTATTACTAATGTAACTGACTCA
GCATCTATGGGGAATTATTTAGCAGATGCAGGACTAGCTATTTTAGATACGTCAGGTGCTATAGACA
CCTTTGTTGTACAAGGTGGATATGGTCTCAATTATTATAAGGTTAACCCGTGTGAAGATGTTAACCA
GCAGTTTGTAGTGTCAGGCGGTAAGTTAGTAGGCATTCTGACTTCTCGTAATGAAACTGATTCTTAT
CCTCTTGAAAATCAGTTTTATATTAAGTTAACTAATGGAAGCCGTCGTTCTAGACGT

>Trachea6

ATGTCGGTAACACCTCTTTTATTAGTGACTCTTTTGTTTGCACTATGTAGTGCTGCTTTGTATGACAAA
GGTTCTTATGTTTACTACTACCAAAGTGCCTTTAGACCACCAGATGGTTGGCATTTACAAGGAGGT
GCGTATGCAGTAGTTAATTCTACTAATTACTCTAATAATGCAGGCGACGCAGCACTATGTACTGGTG
GTTTGCTTACAGATGTTTACAACAACACAGCTGCTGCTATATCTATGGTAGCACCGGCCTCAGGTA
TGAGTTGGTCTACGTCACAGTTTTGTACTGCTCATTGTAGATTCTCAGACCTTACTGTGTTTGTTACG
CATTGTTATAATGCGTCTACGGGTGCCTGCCCTACAACAGGTTTTGTACCAAAGTATCATATTCGC
ATTTCTGCTATGAGAAATGGTTCTTTTCTTTATAACTCAACATTTAGTGTGGTTAAACATCCTAAGTTTT
ATTCTTTTCAATGTGTTAACAACCAAACATCTGTGTATCTTAATGGTGATCTTGTTTACACTTCCAACA
TCACCACTGATGTTACGTCAGCAGGTGTGCATTTTAAAGCAGGTGGACCTGTAAATTATAATGTTAT
GAAAGAATTTCAGGTACTTGCTTATTTTGTTAATGGAACTGTACAAGACGTTATCTTGTGCGATGAAA
CACCTAGAGGTTTATTAGCATGTCAATATAATACTGGCAATTTTTCAGATGGATTTACCCTTTTACTA
ATAATACTTTAGTAAAACAGAAGTTCATTGTTTATCGGGAGAATAGTGTTAATACTACTTTGGTTTTGC

ATAATGTTACTTTTAGTAATGAGACTAATGCACAACCTAATATAGGTGGTGTTGATAATATTAATTTATA
CCAAACATATACAGCTCAGAGTGGTTATTATAATTTTAATTTTTCCTTTCTGAGTGGTTTTGTCTATAAG
GAGTCTGATTTTATGTATGGATCTTATCACCCAAGTTGTAAGTTTAGACCAGAAACTATTAATAATGG
CTTGTGGTTTAATTCACTTTCAATTTCACTTGCATATGGCCCCCTTCAAGGTGGGTGTAAGCAGTC
AGTTTTTAGTCGTAGGGCTACTTGTTGTTATGCCTACTCTTATAGAGGACCACATAAGTGTAAAGGA
GTTTATAGTGGTGAGTTATTAAAAGATTTTGAATGTGGGCTGTTGGTTTATGTTACTAAGAGTGATGG
CTCTCGCATACAAACAGCCACAGAATCACCAGTTATAACTCAACACAATTATAATAATATTACTTTA
AATACGTGTGTTGAGTATAATATATATGGCAGAGTTGGACAAGGTTTTATTACTAATGTAACTGACTCA
GCATCTATGGGGAATTATTTAGCAGATGCAGGACTAGCTATTTTAGATACGTCAGGTGCTATAGACA
CCTTTGTTGTACAAGGTGGATATGGTCTCAATTATTATAAGGTTAACCCGTGTGAAGATGTTAACCA
GCAGTTTGTAGTGTCAGGCGGTAAGTTAGTAGGCATTCTGACTTCTCGTAATGAAACTGATTCTTAT
CCTCTTGAAAATCAGTTTTATATTAAGTTAACTAATGGAAGCCGTCGTTCTAGACGT

>Kidney1

ATGTCGGTAACACCTCTTTTATTAGTGACTCTTTTGTTTGCACTATGTAGTGCTGCTTTGTATGACAAA
GGTTCTTATGTTTACTACTACCAAAGTGCCTTTAGACCACCAGATGGTTGGCATTTACAAGGAGGT
GCGTATGCAGTAGTTAATTCTACTAATTACTCTAATAATGCAGGCGACGCAGCACTATGTACTGGTG
GTTTGCTTACAGATGTTTACAACAACACAGCTGCTGCTATATCTATGGTAGCACCGGCCTCAGGTA
TGAGTTGGTCTACGTCACAGTTTTGTACTGCTCATTGTAGATTCTCAGACCTTACTGTGTTTGTTACG
CATTGTTATAATGCGTCTACGGGTGCCTGCCCTACAACAGGTTTTGTACCACAGAATCATATTCGC
ATTTCTGCTATGAGAAATGGTTCTTTTCTTTATAACTCAACATTTAGTGTGGTTAAACATCCTAAGTTTT
ATTCTTTTCAATGTGTTAACAACCAAACATCTGTGTATCTTAATGGTGATCTTGTTTACACTTCCAACA
TCACCACTGATGTTACGTCAGCAGGTGTGCATTTTAAAGCAGGTGGACCTGTAAATTATAATGTTAT
GAAAGAATTTCAGGTACTTGCTTATTTTGTTAATGGAACTGTACAAGACGTTATCTTGTGCGATGAAA
CACCTAGAGGTTTATTAGCATGTCAATATAATACTGGCAATTTTTCAGATGGATTTTACCCTTTTACTA
ATAATACTTTAGTAAAACAGAAGTTCATTGTTTATCGGGAGAATAGTGTTAATACTACTTTGGTTTTGC
ATAATGTTACTTTTAGTAATGAGACTAATGCACAACCTAATATAGGTGGTGTTGATAATATTAATTTATA
CCAAACATATACAGCTCAGAGTGGTTATTATAATTTTAATTTTTCCTTTCTGAGTGGTTTTGTCTATAAG
GAGTCTGATTTTATGTATGGATCTTATCACCCAAGTTGTAAGTTTAGACCAGAAACTATTAATAATGG
CTTGTGGTTTAATTCACTTTCAATTTCACTTGCATATGGCCCCCTTCAAGGTGGGTGTAAGCAGTC
AGTTTTTAGTCGTAGGGCTACTTGTTGTTATGCCTACTCTTATAGAGGACCACATAAGTGTAAAGGA
GTTTATAGTGGTGAGTTATTAAAAGATTTTGAATGTGGGCTGTTGGTTTATGTTACTAAGAGTGATGG
CTCTCGCATACAAACAGCCACAGAATCACCAGTTATAACTCAACACAATTATAATAATATTACTTTA
AATACGTGTGTTGAGTATAATATATATGGCAGAGTTGGACAAGGTTTTATTACTAATGTAACTGACTCA
GCATCTATGGGGAATTATTTAGCAGATGCAGGACTAGCTATTTTAGATACGTCAGGTGCTATAGACA
CCTTTGTTGTACAAGGTGGATATGGTCTCAATTATTATAAGGTTAACCCGTGTGAAGATGTTAACCA

GCAGTTTGTAGTGTCAGGCGGTAAGTTAGTAGGCATTCTGACTTCTCGTAATGAAACTGATTCTTAT
CCTCTTGAAAATCAGTTTTATATTAAGTTAACTAATGGAAGCCGTCGTTCTAGACGT

>Kidney3

ATGTCGGTAACACCTCTTTTATTAGTGACTCTTTTGTTTGCACTATGTAGTGCTGCTTTGTATGACAAA
GGTTCTTATGTTTACTACTACCAAAGTGCCTTTAGACCACCAGATGGTTGGCATTTACAAGGAGGT
GCGTATGCAGTAGTTAATTCTACTAATTACTCTAATAATGCAGGCGACGCAGCACTATGTACTGGTG
GTTTGCTTACAGATGTTTACAACAACACAGCTGCTGCTATATCTATGGTAGCACCGGCCTCAGGTA
TGAGTTGGTCTACGTCACAGTTTTGTACTGCTCATTGTAGATTCTCAGACCTTACTGTGTTTGTTACG
CATTGTTATAATGCGTCTACGGGTGCCTGCCCTACAACAGGTTTTGTACCACAGAATCATATTCGC
ATTTCTGCTATGAGAAATGGTTCTTTTCTTTATAACTCAACATTTAGTGTGGTTAAACATCCTAAGTTTT
ATTCTTTTCAATGTGTTAACAACCAAACATCTGTGTATCTTAATGGTGATCTTGTTTACACTTCCAACA
TCACCACTGATGTTACGTCAGCAGGTGTGCATTTTAAAGCAGGTGGACCTGTAAATTATAATGTTAT
GAAAGAATTTCAGGTACTTGCTTATTTTGTTAATGGAACTGTACAAGACGTTATCTTGTGCGATGAAA
CACCTAGAGGTTTATTAGCATGTCAATATAATACTGGCAATTTTTCAGATGGATTTTACCCTTTTACTA
ATAATACTTTAGTAAAACAGAAGTTCATTGTTTATCGGGAGAATAGTGTTAATACTACTTTGGTTTTGC
ATAATGTTACTTTTAGTAATGAGACTAATGCACAACCTAATATAGGTGGTGTTGATAATATTAATTTATA
CCAAACATATACAGCTCAGAGTGGTTATTATAATTTTAATTTTTCCTTTCTGAGTGGTTTTGTCTATAAG
GAGTCTGATTTTATGTATGGATCTTATCACCCAAGTTGTAAGTTTAGACCAGAAACTATTAATAATGG
CTTGTGGTTTAATTCACTTTCAATTTCACTTGCATATGGCCCCCTTCAAGGTGGGTGTAAGCAGTC
AGTTTTTAGTCGTAGGGCTACTTGTTGTTATGCCTACTCTTATAGAGGACCACATAAGTGTAAAGGA
GTTTATAGTGGTGAGTTATTAAAAGATTTTGAATGTGGGCTGTTGGTTTATGTTACTAAGAGTGATGG
CTCTCGCATACAAACAGCCACAGAATCACCAGTTATAACTCAACACAATTATAATAATATTACTTTA
AATACGTGTGTTGAGTATAATATATATGGCAGAGTTGGACAAGGTTTTATTACTAATGTAACTGACTCA
GCATCTATGGGGAATTATTTAGCAGATGCAGGACTAGCTATTTTAGATACGTCAGGTGCTATAGACA
CCTTTGTTGTACAAGGTGGATATGGTCTCAATTATTATAAGGTTAACCCGTGTGAAGATGTTAACCA
GCAGTTTGTAGTGTCAGGCGGTAAGTTAGTAGGCATTCTGACTTCTCGTAATGAAACTGATTCTTAT
CCTCTTGAAAATCAGTTTTATATTAAGTTAACTAATGGAAGCCGTCGTTCTAGACGT

>Kidney4

ATGTCGGTAACACCTCTTTTATTAGTGACTCTTTTGTTTGCACTATGTAGTGCTGCTTTGTATGACAAA
GGTTCTTATGTTTACTACTACCAAAGTGCCTTTAGACCACCAGATGGTTGGCATTTACAAGGAGGT
GCGTATGCAGTAGTTAATTCTACTAATTACTCTAATAATGCAGGCGACGCAGCACTATGTACTGGTG
GTTTGCTTACAGATGTTTACAACAACACAGCTGCTGCTATATCTATGGTAGCACCGGCCTCAGGTA
TGAGTTGGTCTACGTCACAGTTTTGTACTGCTCATTGTAGATTCTCAGACCTTACTGTGTTTGTTACG

CATTGTTATAATGCGTCTACGGGTGCCTGCCCTACAACAGGTTTTGTACCAAAGAATCATATTCGC
ATTTCTGCTATGAGAAATGGTTCTTTTCTTTATAACTCAACATTTAGTGTGGTTAAACATCCTAAGTTTT
ATTCTTTTCAATGTGTTAACAACCAAACATCTGTGTATCTTAATGGTGATCTTGTTTACACTTCCAACA
TCACCACTGATGTTACGTCAGCAGGTGTGCATTTTAAAGCAGGTGGACCTGTAAATTATAATGTTAT
GAAAGAATTTCAGGTACTTGCTTATTTTGTTAATGGAACTGTACAAGACGTTATCTTGTGCGATGAAA
CACCTAGAGGTTTATTAGCATGTCAATATAATACTGGCAATTTTTCAGATGGATTTTACCCTTTTACTA
ATAATACTTTAGTAAAACAGAAGTTCATTGTTTATCGGGAGAATAGTGTTAATACTACTTTGGTTTTGC
ATAATGTTACTTTTAGTAATGAGACTAATGCACAACCTAATATAGGTGGTGTTGATAATATTAATTTATA
CCAAACATATACAGCTCAGAGTGGTTATTATAATTTTAATTTTTCCTTTCTGAGTGGTTTTGTCTATAAG
GAGTCTGATTTTATGTATGGATCTTATCACCCAAGTTGTAAGTTTAGACCAGAAACTATTAATAATGG
CTTGTGGTTTAATTCACTTTCAATTTCACTTGCATATGGCCCCCTTCAAGGTGGGTGTAAGCAGTC
AGTTTTTAGTCGTAGGGCTACTTGTTGTTATGCCTACTCTTATAGAGGACCACATAAGTGTAAAGGA
GTTTATAGTGGTGAGTTATTAAAAGATTTTGAATGTGGGCTGTTGGTTTATGTTACTAAGAGTGATGG
CTCTCGCATACAAACAGCCACAGAATCACCAGTTATAACTCAACACAATTATAATAATATTACTTTA
AATACGTGTGTTGAGTATAATATATATGGCAGAGTTGGACAAGGTTTTATTACTAATGTAACTGACTCA
GCATCTATGGGGAATTATTTAGCAGATGCAGGACTAGCTATTTTAGATACGTCAGGTGCTATAGACA
CCTTTGTTGTACAAGGTGGATATGGTCTCAATTATTATAAGGTTAACCCGTGTGAAGATGTTAACCA
GCAGTTTGTAGTGTCAGGCGGTAAGTTAGTAGGCATTCTGACTTCTCGTAATGAAACTGATTCTTAT
CCTCTTGAAAATCAGTTTTATATTAAGTTAACTAATGGAAGCCGTCGTTCTAGACGT

>Kidney5

ATGTCGGTAACACCTCTTTTATTAGTGACTCTTTTGTTTGCACTATGTAGTGCTGCTTTGTATGACAAA
GGTTCTTATGTTTACTACTACCAAAGTGCCTTTAGACCACCAGATGGTTGGCATTTACAAGGAGGT
GCGTATGCAGTAGTTAATTCTACTAATTACTCTAATAATGCAGGCGACGCAGCACTATGTACTGGTG
GTTTGCTTACAGATGTTTACAACAACACAGCTGCTGCTATATCTATGGTAGCACCGGCCTCAGGTA
TGAGTTGGTCTACGTCACAGTTTTGTACTGCTCATTGTAGATTCTCAGACCTTACTGTGTTTGTTACG
CATTGTTATAATGCGTCTACGGGTGCCTGCCCTACAACAGGTTTTGTACCACAGAATCATATTCGC
ATTTCTGCTATGAGAAATGGTTCTTTTCTTTATAACTCAACATTTAGTGTGGTTAAACATCCTAAGTTTT
ATTCTTTTCAATGTGTTAACAACCAAACATCTGTGTATCTTAATGGTGATCTTGTTTACACTTCCAACA
TCACCACTGATGTTACGTCAGCAGGTGTGCATTTTAAAGCAGGTGGACCTGTAAATTATAATGTTAT
GAAAGAATTTCAGGTACTTGCTTATTTTGTTAATGGAACTGTACAAGACGTTATCTTGTGCGATGAAA
CACCTAGAGGTTTATTAGCATGTCAATATAATACTGGCAATTTTTCAGATGGATTTTACCCTTTTACTA
ATAATACTTTAGTAAAACAGAAGTTCATTGTTTATCGGGAGAATAGTGTTAATACTACTTTGGTTTTGC
ATAATGTTACTTTTAGTAATGAGACTAATGCACAACCTAATATAGGTGGTGTTGATAATATTAATTTATA
CCAAACATATACAGCTCAGAGTGGTTATTATAATTTTAATTTTTCCTTTCTGAGTGGTTTTGTCTATAAG
GAGTCTGATTTTATGTATGGATCTTATCACCCAAGTTGTAAGTTTAGACCAGAAACTATTAATAATGG

CTTGTGGTTTAATTCACTTTCAATTTCACTTGCATATGGCCCCCTTCAAGGTGGGTGTAAGCAGTC
AGTTTTTAGTCGTAGGGCTACTTGTTGTTATGCCTACTCTTATAGAGGACCACATAAGTGTAAAGGA
GTTTATAGTGGTGAGTTATTAAAAGATTTTGAATGTGGGCTGTTGGTTTATGTTACTAAGAGTGATGG
CTCTCGCATACAAACAGCCACAGAATCACCAGTTATAACTCAACACAATTATAATAATATTACTTTA
AATACGTGTGTTGAGTATAATATATATGGCAGAGTTGGACAAGGTTTTATTACTAATGTAACTGACTCA
GCATCTATGGGGAATTATTTAGCAGATGCAGGACTAGCTATTTTAGATACGTCAGGTGCTATAGACA
CCTTTGTTGTACAAGGTGGATATGGTCTCAATTATTATAAGGTTAACCCGTGTGAAGATGTTAACCA
GCAGTTTGTAGTGTCAGGCGGTAAGTTAGTAGGCATTCTGACTTCTCGTAATGAAACTGATTCTTAT
CCTCTTGAAAATCAGTTTTATATTAAGTTAACTAATGGAAGCCGTCGTTCTAGACGT

>Kidney6

ATGTCGGTAACACCTCTTTTATTAGTGACTCTTTTGTTTGCACTATGTAGTGCTGCTTTGTATGACAAA
GGTTCTTATGTTTACTACTACCAAAGTGCCTTTAGACCACCAGATGGTTGGCATTTACAAGGAGGT
GCGTATGCAGTAGTTAATTCTACTAATTACTCTAATAATGCAGGCGACGCAGCACTATGTACTGGTG
GTTTGCTTACAGATGTTTACAACAACACAGCTGCTGCTATATCTATGGTAGCACCGGCCTCAGGTA
TGAGTTGGTCTACGTCACAGTTTTGTACTGCTCATTGTAGATTCTCAGACCTTACTGTGTTTGTTACG
CATTGTTATAATGCGTCTACGGGTGCCTGCCCTACAACAGGTTTTGTACCAAAGAATCATATTCGC
ATTTCTGCTATGAGAAATGGTTCTTTTCTTTATAACTCAACATTTAGTGTGGTTAAACATCCTAAGTTTT
ATTCTTTTCAATGTGTTAACAACCAAACATCTGTGTATCTTAATGGTGATCTTGTTTACACTTCCAACA
TCACCACTGATGTTACGTCAGCAGGTGTGCATTTTAAAGCAGGTGGACCTGTAAATTATAATGTTAT
GAAAGAATTTCAGGTACTTGCTTATTTTGTTAATGGAACTGTACAAGACGTTATCTTGTGCGATGAAA
CACCTAGAGGTTTATTAGCATGTCAATATAATACTGGCAATTTTTCAGATGGATTTTACCCTTTTACTA
ATAATACTTTAGTAAAACAGAAGTTCATTGTTTATCGGGAGAATAGTGTTAATACTACTTTGGTTTTGC
ATAATGTTACTTTTAGTAATGAGACTAATGCACAACCTAATATAGGTGGTGTTGATAATATTAATTTATA
CCAAACATATACAGCTCAGAGTGGTTATTATAATTTTAATTTTTCCTTTCTGAGTGGTTTTGTCTATAAG
GAGTCTGATTTTATGTATGGATCTTATCACCCAAGTTGTAAGTTTAGACCAGAAACTATTAATAATGG
CTTGTGGTTTAATTCACTTTCAATTTCACTTGCATATGGCCCCCTTCAAGGTGGGTGTAAGCAGTC
AGTTTTTAGTCGTAGGGCTACTTGTTGTTATGCCTACTCTTATAGAGGACCACATAAGTGTAAAGGA
GTTTATAGTGGTGAGTTATTAAAAGATTTTGAATGTGGGCTGTTGGTTTATGTTACTAAGAGTGATGG
CTCTCGCATACAAACAGCCACAGAATCACCAGTTATAACTCAACACAATTATAATAATATTACTTTA
AATACGTGTGTTGAGTATAATATATATGGCAGAGTTGGACAAGGTTTTATTACTAATGTAACTGACTCA
GCATCTATGGGGAATTATTTAGCAGATGCAGGACTAGCTATTTTAGATACGTCAGGTGCTATAGACA
CCTTTGTTGTACAAGGTGGATATGGTCTCAATTATTATAAGGTTAACCCGTGTGAAGATGTTAACCA
GCAGTTTGTAGTGTCAGGCGGTAAGTTAGTAGGCATTCTGACTTCTCGTAATGAAACTGATTCTTAT
CCTCTTGAAAATCAGTTTTATATTAAGTTAACTAATGGAAGCCGTCGTTCTAGACGT

>Cecal tonsils1

ATGTCGGTAACACCTCTTTTATTAGTGACTCTTTTGTTTGCACTATGTAGTGCTGCTTTGTATGACAAA
GGTTCTTATGTTTACTACTACCAAAGTGCCTTTAGACCACCAGATGGTTGGCATTTACAAGGAGGT
GCGTATGCAGTAGTTAATTCTACTAATTACTCTAATAATGCAGGCGACGCAGCACTATGTACTGGTG
GTTTGCTTACAGATGTTTACAACAACACAGCTGCTGCTATATCTATGGTAGCACCGGCCTCAGGTA
TGAGTTGGTCTACGTCACAGTTTTGTACTGCTCATTGTAGATTCTCAGACCTTACTGTGTTTGTTACG
CATTGTTATAATGCGTCTACGGGTGCCTGCCCTACAACAGGTTTTGTACCACAGAATCATATTCGC
ATTTCTGCTATGAGAAATGGTTCTTTTCTTTATAACTCAACATTTAGTGTGGTTAAACATCCTAAGTTTT
ATTCTTTTCAATGTGTTAACAACCAAACATCTGTGTATCTTAATGGTGATCTTGTTTACACTTCCAACA
TCACCACTGATGTTACGTCAGCAGGTGTGCATTTTAAAGCAGGTGGACCTGTAAATTATAATGTTAT
GAAAGAATTTCAGGTACTTGCTTATTTTGTTAATGGAACTGTACAAGACGTTATCTTGTGCGATGAAA
CACCTAGAGGTTTATTAGCATGTCAATATAATACTGGCAATTTTTCAGATGGATTTTACCCTTTTACTA
ATAATACTTTAGTAAAACAGAAGTTCATTGTTTATCGGGAGAATAGTGTTAATACTATTTTGGTTTTGCA
TAATGTTACTTTTAGTAATGAGACTAATGCACAACCTAATATAGGTGGTGTTGATAATATTAATTTATAC
CAAACATATACAGCTCAGAGTGGTTATTATAATTTTAATTTTTCCTTTCTGAGTGGTTTTGTCTATAAGG
AGTCTGATTTTATGTATGGATCTTATCACCCAAGTTGTAAGTTTAGACCAGAAACTATTAATAATGGCT
TGTGGTTTAATTCACTTTCAATTTCACTTGCATATGGCCCCCTTCAAGGTGGGTGTAAGCAGTCAG
TTTTTAGTCGTAGGGCTACTTGTTGTTATGCCTACTCTTATAGAGGACCACATAAGTGTAAAGGAGTT
TATAGTGGTGAGTTATTAAAAGATTTTGAATGTGGGCTGTTGGTTTATGTTACTAAGAGTGATGGCTCT
CGCATACAAACAGCCACAGAATCACCAGTTATAACTCAACACAATTATAATAATATTACTTTAAATAC
GTGTGTTGAGTATAATATATATGGCAGAGTTGGACAAGGTTTTATTACTAATGTAACTGACTCAGCAT
CTATGGGGAATTATTTAGCAGATGCAGGACTAGCTATTTTAGATACGTCAGGTGCTATAGACACCTT
TGTTGTACAAGGTGGATATGGTCTCAATTATTATAAGGTTAACCCGTGTGAAGATGTTAACCAGCAG
TTTGTAGTGTCAGGCGGTAAGTTAGTAGGCATTCTGACTTCTCGTAATGAAACTGATTCTTATCCTCT
TGAAAATCAGTTTTATATTAAGTTAACTAATGGAAGCCGTCGTTCTAGACGT

>Cecal tonsils2

ATGTCGGTAACACCTCTTTTATTAGTGACTCTTTTGTTTGCACTATGTAGTGCTGCTTTGTATGACAAA
GGTTCTTATGTTTACTACTACCAAAGTGCCTTTAGACCACCAGATGGTTGGCATTTACAAGGAGGT
GCGTATGCAGTAGTTAATTCTACTAATTACTCTAATAATGCAGGCGACGCAGCACTATGTACTGGTG
GTTTGCTTACAGATGTTTACAACAACACAGCTGCTGCTATATCTATGGTAGCACCGGCCTCAGGTA
TGAGTTGGTCTACGTCACAGTTTTGTACTGCTCATTGTAGATTCTCAGACCTTACTGTGTTTGTTACG
CATTGTTATAATGCGTCTACGGGTGCCTGCCCTACAACAGGTTTTGTACCACAGAATCATATTCGC
ATTTCTGCTATGAGAAATGGTTCTTTTCTTTATAACTCAACATTTAGTGTGGTTAAACATCCTAAGTTTT
ATTCTTTTCAATGTGTTAACAACCAAACATCTGTGTATCTTAATGGTGATCTTGTTTACACTTCCAACA
TCACCACTGATGTTACGTCAGCAGGTGTGCATTTTAAAGCAGGTGGACCTGTAAATTATAATGTTAT

GAAAGAATTTCAGGTACTTGCTTATTTTGTTAATGGAACTGTACAAGACGTTATCTTGTGCGATGAAA
CACCTAGAGGTTTATTAGCATGTCAATATAATACTGGCAATTTTTCAGATGGATTTTACCCTTTTACTA
ATAATACTTTAGTAAAACAGAAGTTCATTGTTTATCGGGAGAATAGTGTTAATACTACTTTGGTTTTGC
ATAATGTTACTTTTAGTAATGAGACTAATGCACAACCTAATATAGGTGGTGTTGATAATATTAATTTATA
CCAAACATATACAGCTCAGAGTGGTTATTATAATTTTAATTTTTCCTTTCTGAGTGGTTTTGTCTATAAG
GAGTCTGATTTTATGTATGGATCTTATCACCCAAGTTGTAAGTTTAGACCAGAAACTATTAATAATGG
CTTGTGGTTTAATTCACTTTCAATTTCACTTGCATATGGCCCCCTTCAAGGTGGGTGTAAGCAGTC
AGTTTTTAGTCGTAGGGCTACTTGTTGTTATGCCTACTCTTATAGAGGACCACATAAGTGTAAAGGA
GTTTATAGTGGTGAGTTATTAAAAGATTTTGAATGTGGGCTGTTGGTTTATGTTACTAAGAGTGATGG
CTCTCGCATACAAACAGCCACAGAATCACCAGTTATAACTCAACACAATTATAATAATATTACTTTA
AATACGTGTGTTGAGTATAATATATATGGCAGAGTTGGACAAGGTTTTATTACTAATGTAACTGACTCA
GCATCTATGGGGAATTATTTAGCAGATGCAGGACTAGCTATTTTAGATACGTCAGGTGCTATAGACA
CCTTTGTTGTACAAGGTGGATATGGTCTCAATTATTATAAGGTTAACCCGTGTGAAGATGTTAACCA
GCAGTTTGTAGTGTCAGGCGGTAAGTTAGTAGGCATTCTGACTTCTCGTAATGAAACTGATTCTTAT
CCTCTTGAAAATCAGTTTTATATTAAGTTAACTAATGGAAGCCGTCGTTCTAGACGT

>Cecal tonsils3

ATGTCGGTAACACCTCTTTTATTAGTGACTCTTTTGTTTGCACTATGTAGTGCTGCTTTGTATGACAAA
GGTTCTTATGTTTACTACTACCAAAGTGCCTTTAGACCACCAGATGGTTGGCATTTACAAGGAGGT
GCATATGCAGTAGTTAATTCTACTAATTACTCTAATAATGCAGGCGACGCAGCACTATGTACTGGTG
GTTTGCTTACAGATGTTTACAACAACACAGCTGCTGCTATATCTATGGTAGCACCGGCCTCAGGTA
TGAGTTGGTCTACGTCACAGTTTTGTACTGCACATTGTAGATTCTCAGACCTTACTGTGTTTGTTACG
CATTGTTATAATGCGTCTACGGGTGCCTGCCCTACAACAGGTTTTGTACCACAGTATCATATTCGC
ATTTCTGCTATGAGAAATGGTTCTTTTCTTTATAACTCAACATTTAGTGTGGTTAAACATCCTAAGTTTT
ATTCTTTTCAATGTGTTAACAACCAAACATCTGTGTATCTTAATGGTGATCTTGTTTACACTTCCAACA
TCACCACTGATGTTACGTCAGCAGGTGTGCATTTTAAAGCAGGTGGACCTGTAAATTATAATGTTAT
GAAAGAATTTCAGGTACTTGCTTATTTTGTTAATGGAACTGTACAAGACGTTATCTTGTGCGATGAAA
CACCTAGAGGTTTATTAGCATGTCAATATAATACTGGCAATTTTTCAGATGGATTTTACCCTTTTACTA
ATAATACTTTAGTAAAACAGAAGTTCATTGTTTATCGGGAGAATAGTGTTAATACTACTTTGGTTTTGC
ATAATGTTACTTTTAGTAATGAGACTAATGCACAACCTAATATAGGTGGTGTTGATAATATTAATTTATA
CCAAACATATACAGCTCAGAGTGGTTATTATAATTTTAATTTTTCCTTTCTGAGTGGTTTTGTCTATAAG
GAGTCTGATTTTATGTATGGATCTTATCACCCAAGTTGTAAGTTTAGACCAGAAACTATTAATAATGG
CTTGTGGTTTAATTCACTTTCAATTTCACTTGCATATGGCCCCCTTCAAGGTGGGTGTAAGCAGTC
AGTTTTTAGTCGTAGGGCTACTTGTTGTTATGCCTACTCTTATAGAGGACCACATAAGTGTAAAGGA
GTTTATAGTGGTGAGTTATTAAAAGATTTTGAATGTGGGCTGTTGGTTTATGTTACTAAGAGTGATGG
CTCTCGCATACAAACAGCCACAGAATCACCAGTTATAACTCAACACAATTATAATAATATTACTTTA

AATACGTGTGTTGAGTATAATATATATGGCAGAGTTGGACAAGGTTTTATTACTAATGTAACTGACTCA
GCATCTATGGGGAATTATTTAGCAGATGCAGGACTAGCTATTTTAGATACGTCAGGTGCTATAGACA
CCTTTGTTGTACAAGGTGGATATGGTCTCAATTATTATAAGGTTAACCCGTGTGAAGATGTTAACCA
GCAGTTTGTAGTGTCAGGCGGTAAGTTAGTAGGCATTCTGACTTCTCGTAATGAAACTGATTCTTAT
CCTCTTGAAAATCAGTTTTATATTAAGTTAACTAATGGAAGCCGTCGTTCTAGACGT

>Cecal tonsils4

ATGTCGGTAACACCTCTTTTATTAGTGACTCTTTTGTTTGCACTATGTAGTGCTGCTTTGTATGACAAA
GGTTCTTATGTTTACTACTACCAAAGTGCCTTTAGACCACCAGATGGTTGGCATTTACAAGGAGGT
GCGTATGCAGTAGTTAATTCTACTAATTACTCTAATAATGCAGGCGACGCAGCACTATGTACTGGTG
GTTTGCTTACAGATGTTTACAACAACACAGCTGCTGCTATATCTATGGTAGCACCGGCCTCAGGTA
TGAGTTGGTCTACGTCACAGTTTTGTACTGCTCATTGTAGATTCTCAGACCTTACTGTGTTTGTTACG
CATTGTTATAATGCGTCTACGGGTGCCTGCCCTACAACAGGTTTTGTACCACAGAATCATATTCGC
ATTTCTGCTATGAGAAATGGTTCTTTTCTTTATAACTCAACATTTAGTGTGGTTAAACATCCTAAGTTTT
ATTCTTTTCAATGTGTTAACAACCAAACATCTGTGTATCTTAATGGTGATCTTGTTTACACTTCCAACA
TCACCACTGATGTTACGTCAGCAGGTGTGCATTTTAAAGCAGGTGGACCTGTAAATTATAATGTTAT
GAAAGAATTTCAGGTACTTGCTTATTTTGTTAATGGAACTGTACAAGACGTTATCTTGTGCGATGAAA
CACCTAGAGGTTTATTAGCATGTCAATATAATACTGGCAATTTTTCAGATGGATTTTACCCTTTTACTA
ATAATACTTTAGTAAAACAGAAGTTCATTGTTTATCGGGAGAATAGTGTTAATACTACTTTGGTTTTGC
ATAATGTTACTTTTAGTAATGAGACTAATGCACAACCTAATATAGGTGGTGTTGATAATATTAATTTATA
CCAAACATATACAGCTCAGAGTGGTTATTATAATTTTAATTTTTCCTTTCTGAGTGGTTTTGTCTATAAG
GAGTCTGATTTTATGTATGGATCTTATCACCCAAGTTGTAAGTTTAGACCAGAAACTATTAATAATGG
CTTGTGGTTTAATTCACTTTCAATTTCACTTGCATATGGCCCCCTTCAAGGTGGGTGTAAGCAGTC
AGTTTTTAGTCGTAGGGCTACTTGTTGTTATGCCTACTCTTATAGAGGACCACATAAGTGTAAAGGA
GTTTATAGTGGTGAGTTATTAAAAGATTTTGAATGTGGGCTGTTGGTTTATGTTACTAAGAGTGATGG
CTCTCGCATACAAACAGCCACAGAATCACCAGTTATAACTCAACACAATTATAATAATATTACTTTA
AATACGTGTGTTGAGTATAATATATATGGCAGAGTTGGACAAGGTTTTATTACTAATGTAACTGACTCA
GCATCTATGGGGAATTATTTAGCAGATGCAGGACTAGCTATTTTAGATACGTCAGGTGCTATAGACA
CCTTTGTTGTACAAGGTGGATATGGTCTCAATTATTATAAGGTTAACCCGTGTGAAGATGTTAACCA
GCAGTTTGTAGTGTCAGGCGGTAAGTTAGTAGGCATTCTGACTTCTCGTAATGAAACTGATTCTTAT
CCTCTTGAAAATCAGTTTTATATTAAGTTAACTAATGGAAGCCGTCGTTCTAGACGT

>Cecal tonsils5

ATGTCGGTAACACCTCTTTTATTAGTGACTCTTTTGTTTGCACTATGTAGTGCTGCTTTGTATGACAAA
GGTTCTTATGTTTACTACTACCAAAGTGCCTTTAGACCACCAGATGGTTGGCATTTACAAGGAGGT

GCGTATGCAGTAGTTAATTCTACTAATTACTCTAATAATGCAGGCGACGCAGCACTATGTACTGGTG
GTTTGCTTACAGATGTTTACAACAACACAGCTGCTGCTATATCTATGGTAGCACCGGCCTCAGGTA
TGAGGGGGTCTACGTCACAGTTTTGTACTGCTCATTGTAGATTCTCAGACCTTACTGTGTTTGTTAC
GCATTGTTATAATGCGTCTACGGGTGCCTGCCCTACAACAGGTTTTGTACCACAGTATCATATTCG
CATTTCTGCTATGAGAAATGGTTCTTTTCTTTATAACTCAACATTTAGTGTGGTTAAACATCCTAAGTT
TTATTCTTTTCAATGTGTTAACAACCAAACATCTGTGTATCTTAATGGTGATCTTGTTTACACTTCCAA
CATCACCACTGATGTTACGTCAGCAGGTGTGCATTTTAAAGCAGGTGGACCTGTAAATTATAATGTT
ATGAAAGAATTTCAGGTACTTGCTTATTTTGTTAATGGAACTGTACAAGACGTTATCTTGTGCGATGA
AACACCTAGAGGTTTATTAGCATGTCAATATAATACTGGCAATTTTTCAGATGGATTTTACCCTTTTAC
TAATAATACTTTAGTAAAACAGAAGTTCATTGTTTATCGGGAGAATAGTGTTAATACTACTTTGGTTTTG
CATAATGTTACTTTTAGTAATGAGACTAATGCACAACCTAATATAGGTGGTGTTGATAATATTAATTTAT
ACCAAACATATACAGCTCAGAGTGGTTATTATAATTTTAATTTTTCCTTTCTGAGTGGTTTTGTCTATAA
GGAGTCTGATTTTATGTATGGATCTTATCACCCAAGTTGTAAGTTTAGACCAGAAACTATTAATAATG
GCTTGTGGTTTAATTCACTTTCAATTTCACTTGCATATGGCCCCCTTCAAGGTGGGTGTAAGCAGT
CAGTTTTTAGTCGTAGGGCTACTTGTTGTTATGCCTACTCTTATAGAGGACCACATAAGTGTAAAGG
AGTTTATAGTGGTGAGTTATTAAAAGATTTTGAATGTGGGCTGTTGGTTTATGTTACTAAGAGTGATGG
CTCTCGCATACAAACAGCCACAGAATCACCAGTTATAACTCAACACAATTATAATAATATTACTTTA
AATACGTGTGTTGAGTATAATATATATGGCAGAGTTGGACAAGGTTTTATTACTAATGTAACTGACTCA
GCATCTATGGGGAATTATTTAGCAGATGCAGGACTAGCTATTTTAGATACGTCAGGTGCTATAGACA
CCTTTGTTGTACAAGGTGGATATGGTCTCAATTATTATAAGGTTAACCCGTGTGAAGATGTTAACCA
GCAGTTTGTAGTGTCAGGCGGTAAGTTAGTAGGCATTCTGACTTCTCGTAATGAAACTGATTCTTAT
CCTCTTGAAAATCAGTTTTATATTAAGTTAACTAATGGAAGCCGTCGTTCTAGACGT

>Cecal tonsils6

ATGTCGGTAACACCTCTTTTATTAGTGACTCTTTTGTTTGCACTATGTAGTGCTGCTTTGTATGACAAA
GGTTCTTATGTTTACTACTACCAAAGTGCCTTTAGACCACCAGATGGTTGGCATTTACAAGGAGGT
GCATATGCAGTAGTTAATTCTACTAATTACTCTAATAATGCAGGCGACGCAGCACTATGTACTGGTG
GTTTGCTTACAGATGTTTACAACAACACAGCTGCTGCTATATCTATGGTAGCACCGGCCTCAGGTA
TGAGTTGGTCTACGTCACAGTTTTGTACTGCACATTGTAGATTCTCAGACCTTACTGTGTTTGTTACG
CATTGTTATAATGCGTCTACGGGTGCCTGCCCTACAACAGGTTTTGTACCAAAGTATCATATTCGC
ATTTCTGCTATGAGAAATGGTTCTTTTCTTTATAACTCAACATTTAGTGTGGTTAAACATCCTAAGTTTT
ATTCTTTTCAATGTGTTAACAACCAAACATCTGTGTATCTTAATGGTGATCTTGTTTACACTTCCAACA
TCACCACTGATGTTACGTCAGCAGGTGTGCATTTTAAAGCAGGTGGACCTGTAAATTATAATGTTAT
GAAAGAATTTCAGGTACTTGCTTATTTTGTTAATGGAACTGTACAAGACGTTATCTTGTGCGATGAAA
CACCTAGAGGTTTATTAGCATGTCAATATAATACTGGCAATTTTTCAGATGGATTTTACCCTTTTACTA
ATAATACTTTAGTAAAACAGAAGTTCATTGTTTATCGGGAGAATAGTGTTAATACTACTTTGGTTTTGC

ATAATGTTACTTTTAGTAATGAGACTAATGCACAACCTAATATAGGTGGTGTTGATAATATTAATTTATA
CCAAACATATACAGCTCAGAGTGGTTATTATAATTTAATTTTTCCTTTCTGAGTGGTTTTGTCTATAAG
GAGTCTGATTTTATGTATGGATCTTATCACCCAAGTTGTAAGTTTAGACCAGAAACTATTAATAATGG
CTTGTGGTTTAATTCACTTTCAATTTCACTTGCATATGGCCCCCTTCAAGGTGGGTGTAAGCAGTC
AGTTTTTAGTCGTAGGGCTACTTGTTGTTATGCCTACTCTTATAGAGGACCACATAAGTGTAAAGGA
GTTTATAGTGGTGAGTTATTAAAAGATTTTGAATGTGGGCTGTTGGTTTATGTTACTAAGAGTGATGG
CTCTCGCATACAAACAGCCACAGAATCACCAGTTATAACTCAACACAATTATAATAATATTACTTTA
AATACGTGTGTTGAGTATAATATATATGGCAGAGTTGGACAAGGTTTTATTACTAATGTAACTGACTCA
GCATCTATGGGGAATTATTTAGCAGATGCAGGACTAGCTATTTTAGATACGTCAGGTGCTATAGACA
CCTTTGTTGTACAAGGTGGATATGGTCTCAATTATTATAAGGTTAACCCGTGTGAAGATGTTAACCA
GCAGTTTGTAGTGTCAGGCGGTAAGTTAGTAGGCATTCTGACTTCTCGTAATGAAACTGATTCTTAT
CCTCTTGAAAATCAGTTTTATATTAAGTTAACTAATGGAAGCCGTCGTTCTAGACGT

>Duodenum1

ATGTCGGTAACACCTCTTTTATTAGTGACTCTTTTGTTTGCACTATGTAGTGCTGCTTTGTATGACAAA
GGTTCTTATGTTTACTACTACCAAAGTGCCTTTAGACCACCAGATGGTTGGCATTTACAAGGAGGT
GCGTATGCAGTAGTTAATTCTACTAATTACTCTAATAATGCAGGCGACGCAGCACTATGTACTGGTG
GTTTGCTTACAGATGTTTACAACAACACAGCTGCTGCTATATCTATGGTAGCACCGGCCTCAGGTA
TGAGTTGGTCTACGTCACAGTTTTGTACTGCTCATTGTAGATTCTCAGACCTTACTGTGTTTGTTACG
CATTGTTATAATGCGTCTACGGGTGCCTGCCCTACAACAGGTTTTGTACCACAGAATCATATTCGC
ATTTCTGCTATGAGAAATGGTTCTTTTCTTTATAACTCAACATTTAGTGTGGTTAAACATCCTAAGTTTT
ATTCTTTTCAATGTGTTAACAACCAAACATCTGTGTATCTTAATGGTGATCTTGTTTACACTTCCAACA
TCACCACTGATGTTACGTCAGCAGGTGTGCATTTTAAAGCAGGTGGACCTGTAAATTATAATGTTAT
GAAAGAATTTCAGGTACTTGCTTATTTTGTTAATGGAACTGTACAAGACGTTATCTTGTGCGATGAAA
CACCTAGAGGTTTATTAGCATGTCAATATAATACTGGCAATTTTTCAGATGGATTTTACCCTTTTACTA
ATAATACTTTAGTAAAACAGAAGTTCATTGTTTATCGGGAGAATAGTGTTAATACTACTTTGGTTTTGC
ATAATGTTACTTTTAGTAATGAGACTAATGCACAACCTAATATAGGTGGTGTTGATAATATTAATTTATA
CCAAACATATACAGCTCAGAGTGGTTATTATAATTTAATTTTTCCTTTCTGAGTGGTTTTGTCTATAAG
GAGTCTGATTTTATGTATGGATCTTATCACCCAAGTTGTAAGTTTAGACCAGAAACTATTAATAATGG
CTTGTGGTTTAATTCACTTTCAATTTCACTTGCATATGGCCCCCTTCAAGGTGGGTGTAAGCAGTC
AGTTTTTAGTCGTAGGGCTACTTGTTGTTATGCCTACTCTTATAGAGGACCACATAAGTGTAAAGGA
GTTTATAGTGGTGAGTTATTAAAAGATTTTGAATGTGGGCTGTTGGTTTATGTTACTAAGAGTGATGG
CTCTCGCATACAAACAGCCACAGAATCACCAGTTATAACTCAACACAATTATAATAATATTACTTTA
AATACGTGTGTTGAGTATAATATATATGGCAGAGTTGGACAAGGTTTTATTACTAATGTAACTGACTCA
GCATCTATGGGGAATTATTTAGCAGATGCAGGACTAGCTATTTTAGATACGTCAGGTGCTATAGACA
CCTTTGTTGTACAAGGTGGATATGGTCTCAATTATTATAAGGTTAACCCGTGTGAAGATGTTAACCA

GCAGTTTGTAGTGTCAGGCGGTAAGTTAGTAGGCATTCTGACTTCTCGTAATGAAACTGATTCTTAT
CCTCTTGAAAATCAGTTTTATATTAAGTTAACTAATGGAAGCCGTCGTTCTAGACGT

>Duodenum3

ATGTCGGTAACACCTCTTTTATTAGTGACTCTTTTGTTTGCACTATGTAGTGCTGCTTTGTATGACAAA
GGTTCTTATGTTTACTACTACCAAAGTGCCTTTAGACCACCAGATGGTTGGCATTTACAAGGAGGT
GCGTATGCAGTAGTTAATTCTACTAATTACTCTAATAATGCAGGCGACGCAGCACTATGTACTGGTG
GTTTGCTTACAGATGTTTACAACAACACAGCTGCTGCTATATCTATGGTAGCACCGGCCTCAGGTA
TGAGTTGGTCTACGTCACAGTTTTGTACTGCTCATTGTAGATTCTCAGACCTTACTGTGTTTGTTACG
CATTGTTATAATGCGTCTACGGGTGCCTGCCCTACAACAGGTTTTGTACCACAGAATCATATTCGC
ATTTCTGCTATGAGAAATGGTTCTTTTCTTTATAACTCAACATTTAGTGTGGTTAAACATCCTAAGTTTT
ATTCTTTTCAATGTGTTAACAACCAAACATCTGTGTATCTTAATGGTGATCTTGTTTACACTTCCAACA
TCACCACTGATGTTACGTCAGCAGGTGTGCATTTTAAAGCAGGTGGACCTGTAAATTATAATGTTAT
GAAAGAATTTCAGGTACTTGCTTATTTTGTTAATGGAACTGTACAAGACGTTATCTTGTGCGATGAAA
CACCTAGAGGTTTATTAGCATGTCAATATAATACTGGCAATTTTTCAGATGGATTTTACCCTTTTACTA
ATAATACTTTAGTAAAACAGAAGTTCATTGTTTATCGGGAGAATAGTGTTAATACTACTTTGGTTTTGC
ATAATGTTACTTTTAGTAATGAGACTAATGCACAACCTAATATAGGTGGTGTTGATAATATTAATTTATA
CCAAACATATACAGCTCAGAGTGGTTATTATAATTTTAATTTTTCCTTTCTGAGTGGTTTTGTCTATAAG
GAGTCTGATTTTATGTATGGATCTTATCACCCAAGTTGTAAGTTTAGACCAGAAACTATTAATAATGG
CTTGTGGTTTAATTCACTTTCAATTTCACTTGCATATGGCCCCCTTCAAGGTGGGTGTAAGCAGTC
AGTTTTTAGTCGTAGGGCTACTTGTTGTTATGCCTACTCTTATAGAGGACCACATAAGTGTAAAGGA
GTTTATAGTGGTGAGTTATTAAAAGATTTTGAATGTGGGCTGTTGGTTTATGTTACTAAGAGTGATGG
CTCTCGCATACAAACAGCCACAGAATCACCAGTTATAACTCAACACAATTATAATAATATTACTTTA
AATACGTGTGTTGAGTATAATATATATGGCAGAGTTGGACAAGGTTTTATTACTAATGTAACTGACTCA
GCATCTATGGGGAATTATTTAGCAGATGCAGGACTAGCTATTTTAGATACGTCAGGTGCTATAGACA
CCTTTGTTGTACAAGGTGGATATGGTCTCAATTATTATAAGGTTAACCCGTGTGAAGATGTTAACCA
GCAGTTTGTAGTGTCAGGCGGTAAGTTAGTAGGCATTCTGACTTCTCGTAATGAAACTGATTCTTAT
CCTCTTGAAAATCAGTTTTATATTAAGTTAACTAATGGAAGCCGTCGTTCTAGACGT

>Duodenum4

ATGTCGGTAACACCTCTTTTATTAGTGACTCTTTTGTTTGCACTATGTAGTGCTGCTTTGTATGACAAA
GGTTCTTATGTTTACTACTACCAAAGTGCCTTTAGACCACCAGATGGTTGGCATTTACAAGGAGGT
GCGTATGCAGTAGTTAATTCTACTAATTACTCTAATAATGCAGGCGACGCAGCACTATGTACTGGTG
GTTTGCTTACAGATGTTTACAACAACACAGCTGCTGCTATATCTATGGTAGCACCGGCCTCAGGTA
TGAGTTGGTCTACGTAACAGTTTTGTACTGCTCATTGTAGATTCTCAGACCTTACTGTGTTTGTTACG

CATTGTTATAATGCGTCTACGGGTGCCTGCCCTACAACAGGTTTTGTACCACAGTATCATATTCGC
ATTTCTGCTATGAGAAATGGTTCTTTTCTTTATAACTCAACATTTAGTGTGGTTAAACATCCTAAGTTTT
ATTCTTTTCAATGTGTTAACAACCAAACATCTGTGTATCTTAATGGTGATCTTGTTTACACTTCCAACA
TCACCACTGATGTTACGTCAGCAGGTGTGCATTTTAAAGCAGGTGGACCTGTAAATTATAATGTTAT
GAAAGAATTTCAGGTACTTGCTTATTTTGTTAATGGAACTGTACAAGACGTTATCTTGTGCGATGAAA
CACCTAGAGGTTTATTAGCATGTCAATATAATACTGGCAATTTTTCAGATGGATTTTACCCTTTTACTA
ATAATACTTTAGTAAAACAGAAGTTCATTGTTTATCGGGAGAATAGTGTTAATACTACTTTGGTTTTGC
ATAATGTTACTTTTAGTAATGAGACTAATGCACAACCTAATATAGGTGGTGTTGATAATATTAATTTATA
CCAAACATATACAGCTCAGAGTGGTTATTATAATTTTAATTTTTCCTTTCTGAGTGGTTTTGTCTATAAG
GAGTCTGATTTTATGTATGGATCTTATCACCCAAGTTGTAAGTTTAGACCAGAAACTATTAATAATGG
CTTGTGGTTTAATTCACTTTCAATTTCACTTGCATATGGCCCCCTTCAAGGTGGGTGTAAGCAGTC
AGTTTTTAGTCGTAGGGCTACTTGTTGTTATGCCTACTCTTATAGAGGACCACATAAGTGTAAAGGA
GTTTATAGTGGTGAGTTATTAAAAGATTTTGAATGTGGGCTGTTGGTTTATGTTACTAAGAGTGATGG
CTCTCGCATACAAACAGCCACAGAATCACCAGTTATAACTCAACACAATTATAATAATATTACTTTA
AATACGTGTGTTGAGTATAATATATATGGCAGAGTTGGACAAGGTTTTATTACTAATGTAACTGACTCA
GCATCTATGGGGAATTATTTAGCAGATGCAGGACTAGCTATTTTAGATACGTCAGGTGCTATAGACA
CCTTTGTTGTACAAGGTGGATATGGTCTCAATTATTATAAGGTTAACCCGTGTGAAGATGTTAACCA
GCAGTTTGTAGTGTCAGGCGGTAAGTTAGTAGGCATTCTGACTTCTCGTAATGAAACTGATTCTTAT
CCTCTTGAAAATCAGTTTTATATTAAGTTAACTAATGGAAGCCGTCGTTCTAGACGT

>Duodenum5

ATGTCGGTAACACCTCTTTTATTAGTGACTCTTTTGTTTGCACTATGTAGTGCTGCTTTGTATGACAAA
GGTTCTTATGTTTACTACTACCAAAGTGCCTTTAGACCACCAGATGGTTGGCATTTACAAGGAGGT
GCGTATGCAGTAGTTAATTCTACTAATTACTCTAATAATGCAGGCGACGCAGCACTATGTACTGGTG
GTTTGCTTACAGATGTTTACAACAACACAGCTGCTGCTATATCTATGGTAGCACCGGCCTCAGGTA
TGAGTTGGTCTACGTCACAGTTTTGTACTGCTCATTGTAGATTCTCAGACCTTACTGTGTTAGTTACG
CATTGTTATAAGGCGTCTACGGGTGCCTGCCCTACAACAGGTTTTGTACCACAGTATCATATTCGC
ATTTCTGCTATGAGAAATGGTTCTTTTCTTTATAACTCAACATTTAGTGTGGTTAAACATCCTAAGTTTT
ATTCTTTTCAATGTGTTAACAACCAAACATCTGTGTATCTTAATGGTGATCTTGTTTACACTTCCAACA
TCACCACTGATGTTACGTCAGCAGGTGTGCATTTTAAAGCAGGTGGACCTGTAAATTATAATGTTAT
GAAAGAATTTCAGGTACTTGCTTATTTTGTTAATGGAACTGTACAAGACGTTATCTTGTGCGATGAAA
CACCTAGAGGTTTATTAGCATGTCAATATAATACTGGCAATTTTTCAGATGGATTTTACCCTTTTACTA
ATAATACTTTAGTAAAACAGAAGTTCATTGTTTATCGGGAGAATAGTGTTAATACTATTTTGGTTTTGCA
TAATGTTACTTTTAGTAATGAGACTAATGCACAACCTAATATAGGTGGTGTTGATAATATTAATTTATAC
CAAACATATACAGCTCAGAGTGGTTATTATAATTTTAATTTTTCCTTTCTGAGTGGTTTTGTCTATAAGG
AGTCTGATTTTATGTATGGATCTTATCACCCAAGTTGTAAGTTTAGACCAGAAACTATTAATAATGGCT

TGTGGTTTAATTCACTTTCAATTTCACTTGCATATGGCCCCCTTCAAGGTGGGTGTAAGCAGTCAG
TTTTTAGTCGTAGGGCTACTTGTTGTTATGCCTACTCTTATAGAGGACCACATAAGTGTAAAGGAGTT
TATAGTGGTGAGTTATTAAAAGATTTTGAATGTGGGCTGTTGGTTTATGTTACTAAGAGTGATGGCTCT
CGCATACAAACAGCCACAGAATCACCAGTTATAACTCAACACAATTATAATAATATTACTTTAAATAC
GTGTGTTGAGTATAATATATATGGCAGAGTTGGACAAGGTTTTATTACTAATGTAACTGACTCAGCAT
CTATGGGGAATTATTTAGCAGATGCAGGACTAGCTATTTTAGATACGTCAGGTGCTATAGACACCTT
TGTTGTACAAGGTGGATATGGTCTCAATTATTATAAGGTTAACCCGTGTGAAGATGTTAACCAGCAG
TTTGTAGTGTCAGGCGGTAAGTTAGTAGGCATTCTGACTTCTCGTAATGAAACTGATTCTTATCCTCT
TGAAAATCAGTTTTATATTAAGTTAACTAATGGAAGCCGTCGTTCTAGACGT

>Colorectum1

ATGTCGGTAACACCTCTTTTATTAGTGACTCTTTTGTTTGCACTATGTAGTGCTGCTTTGTATGACAAA
GGTTCTTATGTTTACTACTACCAAAGTGCCTTTAGACCACCAGATGGTTGGCATTTACAAGGAGGT
GCGTATGCAGTAGTTAATTCTACTAATTACTCTAATAATGCAGGCGACGCAGCACTATGTACTGGTG
GTTTGCTTACAGATGTTTACAACAACACAGCTGCTGCTATATCTATGGTAGCACCGGCCTCAGGTA
TGAGTTGGTCTACGTCACAGTTTTGTACTGCTCATTGTAGATTCTCAGACCTTACTGTGTTTGTTACG
CATTGTTATATTGCGTCTACGGGTGCCTGCCCTACAACAGGTTTTGTACCACAGAATCATATTCGC
ATTTCTGCTATGAGAAATGGTTCTTTTCTTTATAACTCAACATTTAGTGTGGTTAAACATCCTAAGTTTT
ATTCTTTTCAATGTGTTAACAACCAAACATCTGTGTATCTTAATGGTGATCTTGTTTACACTTCCAACA
TCACCACTGATGTTACGTCAGCAGGTGTGCATTTTAAAGCAGGTGGACCTGTAAATTATAATGTTAT
GAAAGAATTTCAGGTACTTGCTTATTTTGTTAATGGAACTGTACAAGACGTTATCTTGTGCGATGAAA
CACCTAGAGGTTTATTAGCATGTCAATATAATACTGGCAATTTTTCAGATGGATTTTACCCTTTTACTA
ATAATACTTTAGTAAAACAGAAGTTCATTGTTTATCGGGAGAATAGTGTTAATACTACTTTGGTTTTGC
ATAATGTTACTTTTAGTAATGAGACTAATGCACAACCTAATATAGGTGGTGTTGATAATATTAATTTATA
CCAAACATATACAGCTCAGAGTGGTTATTATAATTTAATTTTTCCTTTCTGAGTGGTTTTGTCTATAAG
GAGTCTGATTTTATGTATGGATCTTATCACCCAAGTTGTAAGTTTAGACCAGAAACTATTAATAATGG
CTTGTGGTTTAATTCACTTTCAATTTCACTTGCATATGGCCCCCTTCAAGGTGGGTGTAAGCAGTC
AGTTTTTAGTCGTAGGGCTACTTGTTGTTATGCCTACTCTTATAGAGGACCACATAAGTGTAAAGGA
GTTTATAGTGGTGAGTTATTAAAAGATTTTGAATGTGGGCTGTTGGTTTATGTTACTAAGAGTGATGG
CTCTCGCATACAAACAGCCACAGAATCACCAGTTATAACTCAACACAATTATAATAATATTACTTTA
AATACGTGTGTTGAGTATAATATATATGGCAGAGTTGGACAAGGTTTTATTACTAATGTAACTGACTCA
GCATCTATGGGGAATTATTTAGCAGATGCAGGACTAGCTATTTTAGATACGTCAGGTGCTATAGACA
CCTTTGTTGTACAAGGTGGATATGGTCTCAATTATTATAAGGTTAACCCGTGTGAAGATGTTAACCA
GCAGTTTGTAGTGTCAGGCGGTAAGTTAGTAGGCATTCTGACTTCTCGTAATGAAACTGATTCTTAT
CCTCTTGAAAATCAGTTTTATATTAAGTTAACTAATGGAAGCCGTCGTTCTAGACGT

>Colorectum2

ATGTCGGTAACACCTCTTTTATTAGTGACTCTTTTGTTTGCACTATGTAGTGCTGCTTTGTATGACAAA
GGTTCTTATGTTTACTACTACCAAAGTGCCTTTAGACCACCAGATGGTTGGCATTTACAAGGAGGT
GCGTATGCAGTAGTTAATTCTACTAATTACTCTAATAATGCAGGCGACGCAGCACTATGTACTGGTG
GTTTGCTTACAGATGTTTACAACAACACAGCTGCTGCTATATCTATGGTAGCACCGGCCTCAGGTA
TGAGTTGGTCTACGTCACAGTTTTGTACTGCTCATTGTAGATTCTCAGACCTTACTGTGTTTGTTACG
CATTGTTATAATGCGTCTACGGGTGCCTGCCCTACAACAGGTTTTGTACCACAGAATCATATTCGC
ATTTCTGCTATGAGAAATGGTTCTTTTCTTTATAACTCAACATTTAGTGTGGTTAAACATCCTAAGTTTT
ATTCTTTTCAATGTGTTAACAACCAAACATCTGTGTATCTTAATGGTGATCTTGTTTACACTTCCAACA
TCACCACTGATGTTACGTCAGCAGGTGTGCATTTTAAAGCAGGTGGACCTGTAAATTATAATGTTAT
GAAAGAATTTCAGGTACTTGCTTATTTTGTTAATGGAACTGTACAAGACGTTATCTTGTGCGATGAAA
CACCTAGAGGTTTATTAGCATGTCAATATAATACTGGCAATTTTTCAGATGGATTTACCCTTTTACTA
ATAATACTTTAGTAAAACAGAAGTTCATTGTTTATCGGGAGAATAGTGTTAATACTATTTTGGTTTTGCA
TAATGTTACTTTTAGTAATGAGACTAATGCACAACCTAATATAGGTGGTGTTGATAATATTAATTTATAC
CAAACATATACAGCTCAGAGTGGTTATTATAATTTTAATTTTTCCTTTCTGAGTGGTTTTGTCTATAAGG
AGTCTGATTTTATGTATGGATCTTATCACCCAAGTTGTAAGTTTAGACCAGAAACTATTAATAATGGCT
TGTGGTTTAATTCACTTTCAATTTCACTTGCATATGGCCCCCTTCAAGGTGGGTGTAAGCAGTCAG
TTTTTAGTCGTAGGGCTACTTGTTGTTATGCCTACTCTTATAGAGGACCACATAAGTGTAAAGGAGTT
TATAGTGGTGAGTTATTAAAAGATTTTGAATGTGGGCTGTTGGTTTATGTTACTAAGAGTGATGGCTCT
CGCATACAAACAGCCACAGAATCACCAGTTATAACTCAACACAATTATAATAATATTACTTTAAATAC
GTGTGTTGAGTATAATATATATGGCAGAGTTGGACAAGGTTTTATTACTAATGTAACTGACTCAGCAT
CTATGGGGAATTATTTAGCAGATGCAGGACTAGCTATTTTAGATACGTCAGGTGCTATAGACACCTT
TGTTGTACAAGGTGGATATGGTCTCAATTATTATAAGGTTAACCCGTGTGAAGATGTTAACCAGCAG
TTTGTAGTGTCAGGCGGTAAGTTAGTAGGCATTCTGACTTCTCGTAATGAAACTGATTCTTATCCTCT
TGAAAATCAGTTTTATATTAAGTTAACTAATGGAAGCCGTCGTTCTAGACGT

>Colorectum3

ATGTCGGTAACACCTCTTTTATTAGTGACTCTTTTGTTTGCACTATGTAGTGCTGCTTTGTATGACAAA
GGTTCTTATGTTTACTACTACCAAAGTGCCTTTAGACCACCAGATGGTTGGCATTTACAAGGAGGT
GCATATGCAGTAGTTAATTCTACTAATTACTCTAATAATGCAGGCGACGCAGCACTATGTACTGGTG
GTTTGCTTACAGATGTTTACAACAACACAGCTGCTGCTATATCTATGGTAGCACCGGCCTCAGGTA
TGAGTTGGTCTACGTCACAGTTTTGTACTGCACATTGTAGATTCTCAGACCTTACTGTGTTTGTTACG
CATTGTTATAATGCGTCTACGGGTGCCTGCCCTACAACAGGTTTTGTACCACAGTATCATATTCGC
ATTTCTGCTATGAGAAATGGTTCTTTTCTTTATAACTCAACATTTAGTGTGGTTAAACATCCTAAGTTTT
ATTCTTTTCAATGTGTTAACAACCAAACATCTGTGTATCTTAATGGTGATCTTGTTTACACTTCCAACA
TCACCACTGATGTTACGTCAGCAGGTGTGCATTTTAAAGCAGGTGGACCTGTAAATTATAATGTTAT

GAAAGAATTTCAGGTACTTGCTTATTTTGTTAATGGAACTGTACAAGACGTTATCTTGTGCGATGAAA
CACCTAGAGGTTTATTAGCATGTCAATATAATACTGGCAATTTTTCAGATGGATTTTACCCTTTTACTA
ATAATACTTTAGTAAAACAGAAGTTCATTGTTTATCGGGAGAATAGTGTTAATACTACTTTGGTTTTGC
ATAATGTTACTTTTAGTAATGAGACTAATGCACAACCTAATATAGGTGGTGTTGATAATATTAATTTATA
CCAAACATATACAGCTCAGAGTGGTTATTATAATTTTAATTTTTCCTTTCTGAGTGGTTTTGTCTATAAG
GAGTCTGATTTTATGTATGGATCTTATCACCCAAGTTGTAAGTTTAGACCAGAAACTATTAATAATGG
CTTGTGGTTTAATTCACTTTCAATTTCACTTGCATATGGCCCCCTTCAAGGTGGGTGTAAGCAGTC
AGTTTTTAGTCGTAGGGCTACTTGTTGTTATGCCTACTCTTATAGAGGACCACATAAGTGTAAAGGA
GTTTATAGTGGTGAGTTATTAAAAGATTTTGAATGTGGGCTGTTGGTTTATGTTACTAAGAGTGATGG
CTCTCGCATACAAACAGCCACAGAATCACCAGTTATAACTCAACACAATTATAATAATATTACTTTA
AATACGTGTGTTGAGTATAATATATATGGCAGAGTTGGACAAGGTTTTATTACTAATGTAACTGACTCA
GCATCTATGGGGAATTATTTAGCAGATGCAGGACTAGCTATTTTAGATACGTCAGGTGCTATAGACA
CCTTTGTTGTACAAGGTGGATATGGTCTCAATTATTATAAGGTTAACCCGTGTGAAGATGTTAACCA
GCAGTTTGTAGTGTCAGGCGGTAAGTTAGTAGGCATTCTGACTTCTCGTAATGAAACTGATTCTTAT
CCTCTTGAAAATCAGTTTTATATTAAGTTAACTAATGGAAGCCGTCGTTCTAGACGT

>Colorectum4

ATGTCGGTAACACCTCTTTTATTAGTGACTCTTTTGTTTGCACTATGTAGTGCTGCTTTGTATGACAAA
GGTTCTTATGTTTACTACTACCAAAGTGCCTTTAGACCACCAGATGGTTGGCATTTACAAGGAGGT
GCGTATGCAGTAGTTAATTCTACTAATTACTCTAATAATGCAGGCGACGCAGCACTATGTACTGGTG
GTTTGCTTACAGATGTTTACAACAACACAGCTGCTGCTATATCTATGGTAGCACCGGCCTCAGGTA
TGAGTTGGTCTACGTCACAGTTTTGTACTGCTCATTGTAGATTCTCAGACCTTACTGTGTTTGTTACG
CATTGTTATAATGCGTCTACGGGTGCCTGCCCTACAACAGGTTTTGTACCACAGAATCATATTCGC
ATTTCTGCTATGAGAAATGGTTCTTTTCTTTATAACTCAACATTTAGTGTGGTTAAACATCCTAAGTTTT
ATTCTTTTCAATGTGTTAACAACCAAACATCTGTGTATCTTAATGGTGATCTTGTTTACACTTCCAACA
TCACCACTGATGTTACGTCAGCAGGTGTGCATTTTAAAGCAGGTGGACCTGTAAATTATAATGTTAT
GAAAGAATTTCAGGTACTTGCTTATTTTGTTAATGGAACTGTACAAGACGTTATCTTGTGCGATGAAA
CACCTAGAGGTTTATTAGCATGTCAATATAATACTGGCAATTTTTCAGATGGATTTTACCCTTTTACTA
ATAATACTTTAGTAAAACAGAAGTTCATTGTTTATCGGGAGAATAGTGTTAATACTATTTTGGTTTTGCA
TAATGTTACTTTTAGTAATGAGACTAATGCACAACCTAATATAGGTGGTGTTGATAATATTAATTTATAC
CAAACATATACAGCTCAGAGTGGTTATTATAATTTTAATTTTTCCTTTCTGAGTGGTTTTGTCTATAAGG
AGTCTGATTTTATGTATGGATCTTATCACCCAAGTTGTAAGTTTAGACCAGAAACTATTAATAATGGCT
TGTGGTTTAATTCACTTTCAATTTCACTTGCATATGGCCCCCTTCAAGGTGGGTGTAAGCAGTCAG
TTTTTAGTCGTAGGGCTACTTGTTGTTATGCCTACTCTTATAGAGGACCACATAAGTGTAAAGGAGTT
TATAGTGGTGAGTTATTAAAAGATTTTGAATGTGGGCTGTTGGTTTATGTTACTAAGAGTGATGGCTCT
CGCATACAAACAGCCACAGAATCACCAGTTATAACTCAACACAATTATAATAATATTACTTTAAATAC

GTGTGTTGAGTATAATATATATGGCAGAGTTGGACAAGGTTTTATTACTAATGTAACTGACTCAGCAT
CTATGGGGAATTATTTAGCAGATGCAGGACTAGCTATTTTAGATACGTCAGGTGCTATAGACACCTT
TGTTGTACAAGGTGGATATGGTCTCAATTATTATAAGGTTAACCCGTGTGAAGATGTTAACCAGCAG
TTTGTAGTGTCAGGCGGTAAGTTAGTAGGCATTCTGACTTCTCGTAATGAAACTGATTCTTATCCTCT
TGAAAATCAGTTTTATATTAAGTTAACTAATGGAAGCCGTCGTTCTAGACGT

>Colorectum5

ATGTCGGTAACACCTCTTTTATTAGTGACTCTTTTGTTTGCACTATGTAGTGCTGCTTTGTATGACAAA
GGTTCTTATGTTTACTACTACCAAAGTGCCTTTAGACCACCAGATGGTTGGCATTTACAAGGAGGT
GCATATGCAGTAGTTAATTCTACTAATTACTCTAATAATGCAGGCGACGCAGCACTATGTACTGGTG
GTTTGCTTACAGATGTTTACAACAACACAGCTGCTGCTATATCTATGGTAGCACCGGCCTCAGGTA
TGAGTTGGTCTACGTCACAGTTTTGTACTGCACATTGTAGATTCTCAGACCTTACTGTGTTTGTTACG
CATTGTTATAATGCGTCTACGGGTGCCTGCCCTACAACAGGTTTTGTACCACAGTATCATATTCGC
ATTTCTGCTATGAGAAATGGTTCTTTTCTTTATAACTCAACATTTAGTGTGGTTAAACATCCTAAGTTTT
ATTCTTTTCAATGTGTTAACAACCAAACATCTGTGTATCTTAATGGTGATCTTGTTTACACTTCCAACA
TCACCACTGATGTTACGTCAGCAGGTGTGCATTTTAAAGCAGGTGGACCTGTAAATTATAATGTTAT
GAAAGAATTTCAGGTACTTGCTTATTTTGTTAATGGAACTGTACAAGACGTTATCTTGTGCGATGAAA
CACCTAGAGGTTTATTAGCATGTCAATATAATACTGGCAATTTTTCAGATGGATTTTACCCTTTTACTA
ATAATACTTTAGTAAAACAGAAGTTCATTGTTTATCGGGAGAATAGTGTTAATACTACTTTGGTTTTGC
ATAATGTTACTTTTAGTAATGAGACTAATGCACAACCTAATATAGGTGGTGTTGATAATATTAATTTATA
CCAAACATATACAGCTCAGAGTGGTTATTATAATTTTAATTTTTCCTTTCTGAGTGGTTTTGTCTATAAG
GAGTCTGATTTTATGTATGGATCTTATCACCCAAGTTGTAAGTTTAGACCAGAAACTATTAATAATGG
CTTGTGGTTTAATTCACTTTCAATTTCACTTGCATATGGCCCCCTTCAAGGTGGGTGTAAGCAGTC
AGTTTTTAGTCGTAGGGCTACTTGTTGTTATGCCTACTCTTATAGAGGACCACATAAGTGTAAAGGA
GTTTATAGTGGTGAGTTATTAAAAGATTTTGAATGTGGGCTGTTGGTTTATGTTACTAAGAGTGATGG
CTCTCGCATACAAACAGCCACAGAATCACCAGTTATAACTCAACACAATTATAATAATATTACTTTA
AATACGTGTGTTGAGTATAATATATATGGCAGAGTTGGACAAGGTTTTATTACTAATGTAACTGACTCA
GCATCTATGGGGAATTATTTAGCAGATGCAGGACTAGCTATTTTAGATACGTCAGGTGCTATAGACA
CCTTTGTTGTACAAGGTGGATATGGTCTCAATTATTATAAGGTTAACCCGTGTGAAGATGTTAACCA
GCAGTTTGTAGTGTCAGGCGGTAAGTTAGTAGGCATTCTGACTTCTCGTAATGAAACTGATTCTTAT
CCTCTTGAAAATCAGTTTTATATTAAGTTAACTAATGGAAGCCGTCGTTCTAGACGT

>Colorectum6

ATGTCGGTAACACCTCTTTTATTAGTGACTCTTTTGTTTGCACTATGTAGTGCTGCTTTGTATGACAAA
GGTTCTTATGTTTACTACTACCAAAGTGCCTTTAGACCACCAGATGGTTGGCATTTACAAGGAGGT

GCATATGCAGTAGTTAATTCTACTAATTACTCTAATAATGCAGGCGACGCAGCACTATGTACTGGTG
GTTTGCTTACAGATGTTTACAACAACACAGCTGCTGCTATATCTATGGTAGCACCGGCCTCAGGTA
TGAGTTGGTCTACGTCACAGTTTTGTACTGCACATTGTAGATTCTCAGACCTTACTGTGTTTGTTACG
CATTGTTATAATGCGTCTACGGGTGCCTGCCCTACAACAGGTTTTGTACCAAAGTATCATATTCGC
ATTTCTGCTATGAGAAATGGTTCTTTTCTTTATAACTCAACATTTAGTGTGGTTAAACATCCTAAGTTTT
ATTCTTTTCAATGTGTTAACAACCAAACATCTGTGTATCTTAATGGTGATCTTGTTTACACTTCCAACA
TCACCACTGATGTTACGTCAGCAGGTGTGCATTTTAAAGCAGGTGGACCTGTAAATTATAATGTTAT
GAAAGAATTTCAGGTACTTGCTTATTTTGTTAATGGAACTGTACAAGACGTTATCTTGTGCGATGAAA
CACCTAGAGGTTTATTAGCATGTCAATATAATACTGGCAATTTTTCAGATGGATTTACCCTTTTACTA
ATAATACTTTAGTAAAACAGAAGTTCATTGTTTATCGGGAGAATAGTGTTAATACTACTTTGGTTTTGC
ATAATGTTACTTTTAGTAATGAGACTAATGCACAACCTAATATAGGTGGTGTTGATAATATTAATTTATA
CCAAACATATACAGCTCAGAGTGGTTATTATAATTTTAATTTTTCCTTTCTGAGTGGTTTTGTCTATAAG
GAGTCTGATTTTATGTATGGATCTTATCACCCAAGTTGTAAGTTTAGACCAGAAACTATTAATAATGG
CTTGTGGTTTAATTCACTTTCAATTTCACTTGCATATGGCCCCCTTCAAGGTGGGTGTAAGCAGTC
AGTTTTTAGTCGTAGGGCTACTTGTTGTTATGCCTACTCTTATAGAGGACCACATAAGTGTAAAGGA
GTTTATAGTGGTGAGTTATTAAAAGATTTTGAATGTGGGCTGTTGGTTTATGTTACTAAGAGTGATGG
CTCTCGCATACAAACAGCCACAGAATCACCAGTTATAACTCAACACAATTATAATAATATTACTTTA
AATACGTGTGTTGAGTATAATATATATGGCAGAGTTGGACAAGGTTTTATTACTAATGTAACTGACTCA
GCATCTATGGGGAATTATTTAGCAGATGCAGGACTAGCTATTTTAGATACGTCAGGTGCTATAGACA
CCTTTGTTGTACAAGGTGGATATGGTCTCAATTATTATAAGGTTAACCCGTGTGAAGATGTTAACCA
GCAGTTTGTAGTGTCAGGCGGTAAGTTAGTAGGCATTCTGACTTCTCGTAATGAAACTGATTCTTAT
CCTCTTGAAAATCAGTTTTATATTAAGTTAACTAATGGAAGCCGTCGTTCTAGACGT

>Ovary2

ATGTCGGTAACACCTCTTTTATTAGTGACTCTTTTGTTTGCACTATGTAGTGCTGCTTTGTATGACAAA
GGTTCTTATGTTTACTACTACCAAAGTGCCTTTAGACCACCAGATGGTTGGCATTTACAAGGAGGT
GCGTATGCAGTAGTTAATTCTACTAATTACTCTAATAATGCAGGCGACGCAGCACTATGTACTGGTG
GTTTGCTTACAGATGTTTACAACAACACAGCTGCTGCTATATCTATGGTAGCACCGGCCTCAGGTA
TGAGTTGGTCTACGTCACAGTTTTGTACTGCTCATTGTAGATTCTCAGACCTTACTGTGTTTGTTACG
CATTGTTATAATGCGTCTACGGGTGCCTGCCCTACAACAGGTTTTGTACCACAGAATCATATTCGC
ATTTCTGCTATGAGAAATGGTTCTTTTCTTTATAACTCAACATTTAGTGTGGTTAAACATCCTAAGTTTT
ATTCTTTTCAATGTGTTAACAACCAAACATCTGTGTATCTTAATGGTGATCTTGTTTACACTTCCAACA
TCACCACTGATGTTACGTCAGCAGGTGTGCATTTTAAAGCAGGTGGACCTGTAAATTATAATGTTAT
GAAAGAATTTCAGGTACTTGCTTATTTTGTTAATGGAACTGTACAAGACGTTATCTTGTGCGATGAAA
CACCTAGAGGTTTATTAGCATGTCAATATAATACTGGCAATTTTTCAGATGGATTTACCCTTTTACTA
ATAATACTTTAGTAAAACAGAAGTTCATTGTTTATCGGGAGAATAGTGTTAATACTACTTTGGTTTTGC

ATAATGTTACTTTTAGTAATGAGACTAATGCACAACCTAATATAGGTGGTGTTGATAATATTAATTTATA
CCAAACATATACAGCTCAGAGTGGTTATTATAATTTAATTTTTCCTTTCTGAGTGGTTTTGTCTATAAG
GAGTCTGATTTTATGTATGGATCTTATCACCCAAGTTGTAAGTTTAGACCAGAAACTATTAATAATGG
CTTGTGGTTTAATTCACTTTCAATTTCACTTGCATATGGCCCCCTTCAAGGTGGGTGTAAGCAGTC
AGTTTTTAGTCGTAGGGCTACTTGTTGTTATGCCTACTCTTATAGAGGACCACATAAGTGTAAAGGA
GTTTATAGTGGTGAGTTATTAAAAGATTTTGAATGTGGGCTGTTGGTTTATGTTACTAAGAGTGATGG
CTCTCGCATACAAACAGCCACAGAATCACCAGTTATAACTCAACACAATTATAATAATATTACTTTA
AATACGTGTGTTGAGTATAATATATATGGCAGAGTTGGACAAGGTTTTATTACTAATGTAACTGACTCA
GCATCTATGGGGAATTATTTAGCAGATGCAGGACTAGCTATTTTAGATACGTCAGGTGCTATAGACA
CCTTTGTTGTACAAGGTGGATATGGTCTCAATTATTATAAGGTTAACCCGTGTGAAGATGTTAACCA
GCAGTTTGTAGTGTCAGGCGGTAAGTTAGTAGGCATTCTGACTTCTCGTAATGAAACTGATTCTTAT
CCTCTTGAAAATCAGTTTTATATTAAGTTAACTAATGGAAGCCGTCGTTCTAGACGT

>Ovary3

ATGTCGGTAACACCTCTTTTATTAGTGACTCTTTTGTTTGCACTATGTAGTGCTGCTTTGTATGACAAA
GGTTCTTATGTTTACTACTACCAAAGTGCCTTTAGACCACCAGATGGTTGGCATTTACAAGGAGGT
GCGTATGCAGTAGTTAATTCTACTAATTACTCTAATAATGCAGGCGACGCAGCACTATGTACTGGTG
GTTTGCTTACAGATGTTTACAACAACACAGCTGCTGCTATATCTATGGTAGCACCGGCCTCAGGTA
TGAGTTGGTCTACGTCACAGTTTTGTACTGCTCATTGTAGATTCTCAGACCTTACTGTGATTGTTACG
CATTGTTATAATGCGTCTACGGGTGCCTGCCCTACAACAGGTTTTGTACCACAGAATCATATTCGC
ATTTCTGCTATGAGAAATGGTTCTTTTCTTTATAACTCAACATTTAGTGTGGTTAAACATCCTAAGTTTT
ATTCTTTTCAATGTGTTAACAACCAAACATCTGTGTATCTTAATGGTGATCTTGTTTACACTTCCAACA
TCACCACTGATGTTACGTCAGCAGGTGTGCATTTTAAAGCAGGTGGACCTGTAAATTATAATGTTAT
GAAAGAATTTCAGGTACTTGCTTATTTTGTTAATGGAACTGTACAAGACGTTATCTTGTGCGATGAAA
CACCTAGAGGTTTATTAGCATGTCAATATAATACTGGCAATTTTTCAGATGGATTTTACCCTTTTACTA
ATAATACTTTAGTAAAACAGAAGTTCATTGTTTATCGGGAGAATAGTGTTAATACTACTTTGGTTTTGC
ATAATGTTACTTTTAGTAATGAGACTAATGCACAACCTAATATAGGTGGTGTTGATAATATTAATTTATA
CCAAACATATACAGCTCAGAGTGGTTATTATAATTTAATTTTTCCTTTCTGAGTGGTTTTGTCTATAAG
GAGTCTGATTTTATGTATGGATCTTATCACCCAAGTTGTAAGTTTAGACCAGAAACTATTAATAATGG
CTTGTGGTTTAATTCACTTTCAATTTCACTTGCATATGGCCCCCTTCAAGGTGGGTGTAAGCAGTC
AGTTTTTAGTCGTAGGGCTACTTGTTGTTATGCCTACTCTTATAGAGGACCACATAAGTGTAAAGGA
GTTTATAGTGGTGAGTTATTAAAAGATTTTGAATGTGGGCTGTTGGTTTATGTTACTAAGAGTGATGG
CTCTCGCATACAAACAGCCACAGAATCACCAGTTATAACTCAACACAATTATAATAATATTACTTTA
AATACGTGTGTTGAGTATAATATATATGGCAGAGTTGGACAAGGTTTTATTACTAATGTAACTGACTCA
GCATCTATGGGGAATTATTTAGCAGATGCAGGACTAGCTATTTTAGATACGTCAGGTGCTATAGACA
CCTTTGTTGTACAAGGTGGATATGGTCTCAATTATTATAAGGTTAACCCGTGTGAAGATGTTAACCA

GCAGTTTGTAGTGTCAGGCGGTAAGTTAGTAGGCATTCTGACTTCTCGTAATGAAACTGATTCTTAT
CCTCTTGAAAATCAGTTTTATATTAAGTTAACTAATGGAAGCCGTCGTTCTAGACGT

>Ovary4

ATGTCGGTAACACCTCTTTTATTAGTGACTCTTTTGTTTGCACTATGTAGTGCTGCTTTGTATGACAAA
GGTTCTTATGTTTACTACTACCAAAGTGCCTTTAGACCACCAGATGGTTGGCATTTACAAGGAGGT
GCGTATGCAGTAGTTAATTCTACTAATTACTCTAATAATGCAGGCGACGCAGCACTATGTACTGGTG
GTTTGCTTACAGATGTTTACAACAACACAGCTGCTGCTATATCTATGGTAGCACCGGCCTCAGGTA
TGAGTTGGTCTACGTCACAGTTTTGTACTGCTCATTGTAGATTCTCAGACCTTACTGTGTTTGTTACG
CATTGTTATAATGCGTCTACGGGTGCCTGCCCTACAACAGGTTTTGTACCACAGAATCATATTCGC
ATTTCTGCTATGAGAAATGGTTCTTTTCTTTATAACTCAACATTTAGTGTGGTTAAACATCCTAAGTTTT
ATTCTTTTCAATGTGTTAACAACCAAACATCTGTGTATCTTAATGGTGATCTTGTTTACACTTCCAACA
TCACCACTGATGTTACGTCAGCAGGTGTGCATTTTAAAGCAGGTGGACCTGTAAATTATAATGTTAT
GAAAGAATTTCAGGTACTTGCTTATTTTGTTAATGGAACTGTACAAGACGTTATCTTGTGCGATGAAA
CACCTAGAGGTTTATTAGCATGTCAATATAATACTGGCAATTTTTCAGATGGATTTTACCCTTTTACTA
ATAATACTTTAGTAAAACAGAAGTTCATTGTTTATCGGGAGAATAGTGTTAATACTACTTTGGTTTTGC
ATAATGTTACTTTTAGTAATGAGACTAATGCACAACCTAATATAGGTGGTGTTGATAATATTAATTTATA
CCAAACATATACAGCTCAGAGTGGTTATTATAATTTTAATTTTTCCTTTCTGAGTGGTTTTGTCTATAAG
GAGTCTGATTTTATGTATGGATCTTATCACCCAAGTTGTAAGTTTAGACCAGAAACTATTAATAATGG
CTTGTGGTTTAATTCACTTTCAATTTCACTTGCATATGGCCCCCTTCAAGGTGGGTGTAAGCAGTC
AGTTTTTAGTCGTAGGGCTACTTGTTGTTATGCCTACTCTTATAGAGGACCACATAAGTGTAAAGGA
GTTTATAGTGGTGAGTTATTAAAAGATTTTGAATGTGGGCTGTTGGTTTATGTTACTAAGAGTGATGG
CTCTCGCATACAAACAGCCACAGAATCACCAGTTATAACTCAACACAATTATAATAATATTACTTTA
AATACGTGTGTTGAGTATAATATATATGGCAGAGTTGGACAAGGTTTTATTACTAATGTAACTGACTCA
GCATCTATGGGGAATTATTTAGCAGATGCAGGACTAGCTATTTTAGATACGTCAGGTGCTATAGACA
CCTTTGTTGTACAAGGTGGATATGGTCTCAATTATTATAAGGTTAACCCGTGTGAAGATGTTAACCA
GCAGTTTGTAGTGTCAGGCGGTAAGTTAGTAGGCATTCTGACTTCTCGTAATGAAACTGATTCTTAT
CCTCTTGAAAATCAGTTTTATATTAAGTTAACTAATGGAAGCCGTCGTTCTAGACGT

>Ovary5

ATGTCGGTAACACCTCTTTTATTAGTGACTCTTTTGTTTGCACTATGTAGTGCTGCTTTGTATGACAAA
GGTTCTTATGTTTACTACTACCAAAGTGCCTTTAGACCACCAGATGGTTGGCATTTACAAGGAGGT
GCGTATGCAGTAGTTAATTCTACTAATTACTCTAATAATGCAGGCGACGCAGCACTATGTACTGGTG
GTTTGCTTACAGATGTTTACAACAACACAGCTGCTGCTATATCTATGGTAGCACCGGCCTCAGGTA
TGAGTTGGTCTACGTCACAGTTTTGTACTGCTCATTGTAGATTCTCAGACCTTACTGTGTTTGTTACG

CATTGTTATAATGCGTCTACGGGTGCCTGCCCTACAACAGGTTTTGTACCAAAGAATCATATTCGC
ATTTCTGCTATGAGAAATGGTTCTTTTCTTTATAACTCAACATTTAGTGTGGTTAAACATCCTAAGTTTT
ATTCTTTTCAATGTGTTAACAACCAAACATCTGTGTATCTTAATGGTGATCTTGTTTACACTTCCAACA
TCACCACTGATGTTACGTCAGCAGGTGTGCATTTTAAAGCAGGTGGACCTGTAAATTATAATGTTAT
GAAAGAATTTCAGGTACTTGCTTATTTTGTTAATGGAACTGTACAAGACGTTATCTTGTGCGATGAAA
CACCTAGAGGTTTATTAGCATGTCAATATAATACTGGCAATTTTTCAGATGGATTTTACCCTTTTACTA
ATAATACTTTAGTAAAACAGAAGTTCATTGTTTATCGGGAGAATAGTGTTAATACTACTTTGGTTTTGC
ATAATGTTACTTTTAGTAATGAGACTAATGCACAACCTAATATAGGTGGTGTTGATAATATTAATTTATA
CCAAACATATACAGCTCAGAGTGGTTATTATAATTTTAATTTTTCCTTTCTGAGTGGTTTTGTCTATAAG
GAGTCTGATTTTATGTATGGATCTTATCACCCAAGTTGTAAGTTTAGACCAGAAACTATTAATAATGG
CTTGTGGTTTAATTCACTTTCAATTTCACTTGCATATGGCCCCCTTCAAGGTGGGTGTAAGCAGTC
AGTTTTTAGTCGTAGGGCTACTTGTTGTTATGCCTACTCTTATAGAGGACCACATAAGTGTAAAGGA
GTTTATAGTGGTGAGTTATTAAAAGATTTTGAATGTGGGCTGTTGGTTTATGTTACTAAGAGTGATGG
CTCTCGCATACAAACAGCCACAGAATCACCAGTTATAACTCAACACAATTATAATAATATTACTTTA
AATACGTGTGTTGAGTATAATATATATGGCAGAGTTGGACAAGGTTTTATTACTAATGTAACTGACTCA
GCATCTATGGGGAATTATTTAGCAGATGCAGGACTAGCTATTTTAGATACGTCAGGTGCTATAGACA
CCTTTGTTGTACAAGGTGGATATGGTCTCAATTATTATAAGGTTAACCCGTGTGAAGATGTTAACCA
GCAGTTTGTAGTGTCAGGCGGTAAGTTAGTAGGCATTCTGACTTCTCGTAATGAAACTGATTCTTAT
CCTCTTGAAAATCAGTTTTATATTAAGTTAACTAATGGAAGCCGTCGTTCTAGACGT

>Ovary6

ATGTCGGTAACACCTCTTTTATTAGTGACTCTTTTGTTTGCACTATGTAGTGCTGCTTTGTATGACAAA
GGTTCTTATGTTTACTACTACCAAAGTGCCTTTAGACCACCAGATGGTTGGCATTTACAAGGAGGT
GCGTATGCAGTAGTTAATTCTACTAATTACTCTAATAATGCAGGCGACGCAGCACTATGTACTGGTG
GTTTGCTTACAGATGTTTACAACAACACAGCTGCTGATATATCTATGGTAGCACCGGACTCAGGTA
TGAGTTGGTCTACGTCACAGTTTTGTAATGCTCATTGTAGATTCTCAGACCTTACTGTGTTTGTTACG
AATTGTTATAATGCGTCTACGGGTGCCTGCCCTACAACAGGTTTTGTACCAAAGTATCATATTCGC
ATTTCTGCTATGAGAAATGGTTCTTTTCTTTATAACTCAACATTTAGTGTGGTTAAACATCCTAAGTTTT
ATTCTTTTCAATGTGTTAACAACCAAACATCTGTGTATCTTAATGGTGATCTTGTTTACACTTCCAACA
TCACCACTGATGTTACGTCAGCAGGTGTGCATTTTAAAGCAGGTGGACCTGTAAATTATAATGTTAT
GAAAGAATTTCAGGTACTTGCTTATTTTGTTAATGGAACTGTACAAGACGTTATCTTGTGCGATGAAA
CACCTAGAGGTTTATTAGCATGTCAATATAATACTGGCAATTTTTCAGATGGATTTTACCCTTTTACTA
ATAATACTTTAGTAAAACAGAAGTTCATTGTTTATCGGGAGAATAGTGTTAATACTACTTTGGTTTTGC
ATAATGTTACTTTTAGTAATGAGACTAATGCACAACCTAATATAGGTGGTGTTGATAATATTAATTTATA
CCAAACATATACAGCTCAGAGTGGTTATTATAATTTTAATTTTTCCTTTCTGAGTGGTTTTGTCTATAAG
GAGTCTGATTTTATGTATGGATCTTATCACCCAAGTTGTAAGTTTAGACCAGAAACTATTAATAATGG

CTTGTGGTTTAATTCACTTTCAATTTCACTTGCATATGGCCCCCTTCAAGGTGGGTGTAAGCAGTC
AGTTTTTAGTCGTAGGGCTACTTGTTGTTATGCCTACTCTTATAGAGGACCACATAAGTGTAAAGGA
GTTTATAGTGGTGAGTTATTAAAAGATTTTGAATGTGGGCTGTTGGTTTATGTTACTAAGAGTGATGG
CTCTCGCATACAAACAGCCACAGAATCACCAGTTATAACTCAACACAATTATAATAATATTACTTTA
AATACGTGTGTTGAGTATAATATATATGGCAGAGTTGGACAAGGTTTTATTACTAATGTAACTGACTCA
GCATCTATGGGGAATTATTTAGCAGATGCAGGACTAGCTATTTTAGATACGTCAGGTGCTATAGACA
CCTTTGTTGTACAAGGTGGATATGGTCTCAATTATTATAAGGTTAACCCGTGTGAAGATGTTAACCA
GCAGTTTGTAGTGTCAGGCGGTAAGTTAGTAGGCATTCTGACTTCTCGTAATGAAACTGATTCTTAT
CCTCTTGAAAATCAGTTTTATATTAAGTTAACTAATGGAAGCCGTCGTTCTAGACGT

>Magnum1

ATGTCGGTAACACCTCTTTTATTAGTGACTCTTTTGTTTGCACTATGTAGTGCTGCTTTGTATGACAAA
GGTTCTTATGTTTACTACTACCAAAGTGCCTTTAGACCACCAGATGGTTGGCATTTACAAGGAGGT
GCGTATGCAGTAGTTAATTCTACTAATTACTCTAATAATGCAGGCGACGCAGCACTATGTACTGGTG
GTTTGCTTACAGATGTTTACAACAACACAGCTGCTGCTATATCTATGGTAGCACCGGCCTCAGGTA
TGAGTTGGTCTACGTCACAGTTTTGTACTGCTCATTGTAGATTCTCAGACCTTTCTGTGTTTGTTACG
CATTGTTATAATGCGTCTACGGGTGCCTGCCCTACAACAGGTTTTGTACCACAGTATCATATTCGC
ATTTCTGCTATGAGAAATGGTTCTTTTCTTTATAACTCAACATTTAGTGTGGTTAAACATCCTAAGTTTT
ATTCTTTTCAATGTGTTAACAACCAAACATCTGTGTATCTTAATGGTGATCTTGTTTACACTTCCAACA
TCACCACTGATGTTACGTCAGCAGGTGTGCATTTTAAAGCAGGTGGACCTGTAAATTATAATGTTAT
GAAAGAATTTCAGGTACTTGCTTATTTTGTTAATGGAACTGTACAAGACGTTATCTTGTGCGATGAAA
CACCTAGAGGTTTATTAGCATGTCAATATAATACTGGCAATTTTTCAGATGGATTTTACCCTTTTACTA
ATAATACTTTAGTAAAACAGAAGTTCATTGTTTATCGGGAGAATAGTGTTAATACTACTTTGGTTTTGC
ATAATGTTACTTTTAGTAATGAGACTAATGCACAACCTAATATAGGTGGTGTTGATAATATTAATTTATA
CCAAACATATACAGCTCAGAGTGGTTATTATAATTTAATTTTTCCTTTCTGAGTGGTTTTGTCTATAAG
GAGTCTGATTTTATGTATGGATCTTATCACCCAAGTTGTAAGTTTAGACCAGAAACTATTAATAATGG
CTTGTGGTTTAATTCACTTTCAATTTCACTTGCATATGGCCCCCTTCAAGGTGGGTGTAAGCAGTC
AGTTTTTAGTCGTAGGGCTACTTGTTGTTATGCCTACTCTTATAGAGGACCACATAAGTGTAAAGGA
GTTTATAGTGGTGAGTTATTAAAAGATTTTGAATGTGGGCTGTTGGTTTATGTTACTAAGAGTGATGG
CTCTCGCATACAAACAGCCACAGAATCACCAGTTATAACTCAACACAATTATAATAATATTACTTTA
AATACGTGTGTTGAGTATAATATATATGGCAGAGTTGGACAAGGTTTTATTACTAATGTAACTGACTCA
GCATCTATGGGGAATTATTTAGCAGATGCAGGACTAGCTATTTTAGATACGTCAGGTGCTATAGACA
CCTTTGTTGTACAAGGTGGATATGGTCTCAATTATTATAAGGTTAACCCGTGTGAAGATGTTAACCA
GCAGTTTGTAGTGTCAGGCGGTAAGTTAGTAGGCATTCTGACTTCTCGTAATGAAACTGATTCTTAT
CCTCTTGAAAATCAGTTTTATATTAAGTTAACTAATGGAAGCCGTCGTTCTAGACGT

>Magnum2

ATGTCGGTAACACCTCTTTTATTAGTGACTCTTTTGTTTGCACTATGTAGTGCTGCTTTGTATGACAAA
GGTTCTTATGTTTACTACTACCAAAGTGCCTTTAGACCACCAGATGGTTGGCATTTACAAGGAGGT
GCGTATGCAGTAGTTAATTCTACTAATTACTCTAATAATGCAGGCGACGCAGCACTATGTACTGGTG
GTTTGCTTACAGATGTTTACAACAACACAGCTGCTGCTATATCTATGGTAGCACCGGCCTCAGGTA
TGAGTTGGTCTACGTCACAGTTTTGTACTGCTCATTGTAGATTCTCAGACCTTACTGTGTTTGTTACG
CATTGTTATAATGCGTCTACGGGTGCCTGCCCTACAACAGGTTTTGTACCACAGAATCATATTCGC
ATTTCTGCTATGAGAAATGGTTCTTTTCTTTATAACTCAACATTTAGTGTGGTTAAACATCCTAAGTTTT
ATTCTTTTCAATGTGTTAACAACCAAACATCTGTGTATCTTAATGGTGATCTTGTTTACACTTCCAACA
TCACCACTGATGTTACGTCAGCAGGTGTGCATTTTAAAGCAGGTGGACCTGTAAATTATAATGTTAT
GAAAGAATTTCAGGTACTTGCTTATTTTGTTAATGGAACTGTACAAGACGTTATCTTGTGCGATGAAA
CACCTAGAGGTTTATTAGCATGTCAATATAATACTGGCAATTTTTCAGATGGATTTTACCCTTTTACTA
ATAATACTTTAGTAAAACAGAAGTTCATTGTTTATCGGGAGAATAGTGTTAATACTACTTTGGTTTTGC
ATAATGTTACTTTTAGTAATGAGACTAATGCACAACCTAATATAGGTGGTGTTGATAATATTAATTTATA
CCAAACATATACAGCTCAGAGTGGTTATTATAATTTTAATTTTTCCTTTCTGAGTGGTTTTGTCTATAAG
GAGTCTGATTTTATGTATGGATCTTATCACCCAAGTTGTAAGTTTAGACCAGAAACTATTAATAATGG
CTTGTGGTTTAATTCACTTTCAATTTCACTTGCATATGGCCCCCTTCAAGGTGGGTGTAAGCAGTC
AGTTTTTAGTCGTAGGGCTACTTGTTGTTATGCCTACTCTTATAGAGGACCACATAAGTGTAAAGGA
GTTTATAGTGGTGAGTTATTAAAAGATTTTGAATGTGGGCTGTTGGTTTATGTTACTAAGAGTGATGG
CTCTCGCATACAAACAGCCACAGAATCACCAGTTATAACTCAACACAATTATAATAATATTACTTTA
AATACGTGTGTTGAGTATAATATATATGGCAGAGTTGGACAAGGTTTTATTACTAATGTAACTGACTCA
GCATCTATGGGGAATTATTTAGCAGATGCAGGACTAGCTATTTTAGATACGTCAGGTGCTATAGACA
CCTTTGTTGTACAAGGTGGATATGGTCTCAATTATTATAAGGTTAACCCGTGTGAAGATGTTAACCA
GCAGTTTGTAGTGTCAGGCGGTAAGTTAGTAGGCATTCTGACTTCTCGTAATGAAACTGATTCTTAT
CCTCTTGAAAATCAGTTTTATATTAAGTTAACTAATGGAAGCCGTCGTTCTAGACGT

>Magnum3

ATGTCGGTAACACCTCTTTTATTAGTGACTCTTTTGTTTGCACTATGTAGTGCTGCTTTGTATGACAAA
GGTTCTTATGTTTACTACTACCAAAGTGCCTTTAGACCACCAGATGGTTGGCATTTACAAGGAGGT
GCATATGCAGTAGTTAATTCTACTAATTACTCTAATAATGCAGGCGACGCAGCACTATGTACTGGTG
GTTTGCTTACAGATGTTTACAACAACACAGCTGCTGCTATATCTATGGTAGCACCGGCCTCAGGTA
TGAGTTGGTCTACGTCACAGTTTTGTACTGCACATTGTAGATTCTCAGACCTTACTGTGTTTGTTACG
CATTGTTATAATGCGTCTACGGGTGCCTGCCCTACAACAGGTTTTGTACCACAGTATCATATTCGC
ATTTCTGCTATGAGAAATGGTTCTTTTCTTTATAACTCAACATTTAGTGTGGTTAAACATCCTAAGTTTT
ATTCTTTTCAATGTGTTAACAACCAAACATCTGTGTATCTTAATGGTGATCTTGTTTACACTTCCAACA
TCACCACTGATGTTACGTCAGCAGGTGTGCATTTTAAAGCAGGTGGACCTGTAAATTATAATGTTAT

GAAAGAATTTCAGGTACTTGCTTATTTTGTTAATGGAACTGTACAAGACGTTATCTTGTGCGATGAAA
CACCTAGAGGTTTATTAGCATGTCAATATAATACTGGCAATTTTTCAGATGGATTTTACCCTTTTACTA
ATAATACTTTAGTAAAACAGAAGTTCATTGTTTATCGGGAGAATAGTGTTAATACTACTTTGGTTTTGC
ATAATGTTACTTTTAGTAATGAGACTAATGCACAACCTAATATAGGTGGTGTTGATAATATTAATTTATA
CCAAACATATACAGCTCAGAGTGGTTATTATAATTTTAATTTTTCCTTTCTGAGTGGTTTTGTCTATAAG
GAGTCTGATTTTATGTATGGATCTTATCACCCAAGTTGTAAGTTTAGACCAGAAACTATTAATAATGG
CTTGTGGTTTAATTCACTTTCAATTTCACTTGCATATGGCCCCCTTCAAGGTGGGTGTAAGCAGTC
AGTTTTTAGTCGTAGGGCTACTTGTTGTTATGCCTACTCTTATAGAGGACCACATAAGTGTAAAGGA
GTTTATAGTGGTGAGTTATTAAAAGATTTTGAATGTGGGCTGTTGGTTTATGTTACTAAGAGTGATGG
CTCTCGCATACAAACAGCCACAGAATCACCAGTTATAACTCAACACAATTATAATAATATTACTTTA
AATACGTGTGTTGAGTATAATATATATGGCAGAGTTGGACAAGGTTTTATTACTAATGTAACTGACTCA
GCATCTATGGGGAATTATTTAGCAGATGCAGGACTAGCTATTTTAGATACGTCAGGTGCTATAGACA
CCTTTGTTGTACAAGGTGGATATGGTCTCAATTATTATAAGGTTAACCCGTGTGAAGATGTTAACCA
GCAGTTTGTAGTGTCAGGCGGTAAGTTAGTAGGCATTCTGACTTCTCGTAATGAAACTGATTCTTAT
CCTCTTGAAAATCAGTTTTATATTAAGTTAACTAATGGAAGCCGTCGTTCTAGACGT

>Magnum4

ATGTCGGTAACACCTCTTTTATTAGTGACTCTTTTGTTTGCACTATGTAGTGCTGCTTTGTATGACAAA
GGTTCTTATGTTTACTACTACCAAAGTGCCTTTAGACCACCAGATGGTTGGCATTTACAAGGAGGT
GCGTATGCAGTAGTTAATTCTACTAATTACTCTAATAATGCAGGCGACGCAGCACTATGTACTGGTG
GTTTGCTTACAGATGTTTACAACAACACAGCTGCTGCTATATCTATGGTAGCACCGGCCTCAGGTA
TGAGTTGGTCTACGTCACAGTTTTGTACTGCTCATTGTAGATTCTCAGACCTTACTGTGTTTGTTACG
CATTGTTATAATGCGTCTACGGGTGCCTGCCCTACAACAGGTTTTGTACCACAGAATCATATTCGC
ATTTCTGCTATGAGAAATGGTTCTTTTCTTTATAACTCAACATTTAGTGTGGTTAAACATCCTAAGTTTT
ATTCTTTTCAATGTGTTAACAACCAAACATCTGTGTATCTTAATGGTGATCTTGTTTACACTTCCAACA
TCACCACTGATGTTACGTCAGCAGGTGTGCATTTTAAAGCAGGTGGACCTGTAAATTATAATGTTAT
GAAAGAATTTCAGGTACTTGCTTATTTTGTTAATGGAACTGTACAAGACGTTATCTTGTGCGATGAAA
CACCTAGAGGTTTATTAGCATGTCAATATAATACTGGCAATTTTTCAGATGGATTTTACCCTTTTACTA
ATAATACTTTAGTAAAACAGAAGTTCATTGTTTATCGGGAGAATAGTGTTAATACTACTTTGGTTTTGC
ATAATGTTACTTTTAGTAATGAGACTAATGCACAACCTAATATAGGTGGTGTTGATAATATTAATTTATA
CCAAACATATACAGCTCAGAGTGGTTATTATAATTTTAATTTTTCCTTTCTGAGTGGTTTTGTCTATAAG
GAGTCTGATTTTATGTATGGATCTTATCACCCAAGTTGTAAGTTTAGACCAGAAACTATTAATAATGG
CTTGTGGTTTAATTCACTTTCAATTTCACTTGCATATGGCCCCCTTCAAGGTGGGTGTAAGCAGTC
AGTTTTTAGTCGTAGGGCTACTTGTTGTTATGCCTACTCTTATAGAGGACCACATAAGTGTAAAGGA
GTTTATAGTGGTGAGTTATTAAAAGATTTTGAATGTGGGCTGTTGGTTTATGTTACTAAGAGTGATGG
CTCTCGCATACAAACAGCCACAGAATCACCAGTTATAACTCAACACAATTATAATAATATTACTTTA

AATACGTGTGTTGAGTATAATATATATGGCAGAGTTGGACAAGGTTTTATTACTAATGTAACTGACTCA
GCATCTATGGGGAATTATTTAGCAGATGCAGGACTAGCTATTTTAGATACGTCAGGTGCTATAGACA
CCTTTGTTGTACAAGGTGGATATGGTCTCAATTATTATAAGGTTAACCCGTGTGAAGATGTTAACCA
GCAGTTTGTAGTGTCAGGCGGTAAGTTAGTAGGCATTCTGACTTCTCGTAATGAAACTGATTCTTAT
CCTCTTGAAAATCAGTTTTATATTAAGTTAACTAATGGAAGCCGTCGTTCTAGACGT

>Magnum5

ATGTCGGTAACACCTCTTTTATTAGTGACTCTTTTGTTTGCACTATGTAGTGCTGCTTTGTATGACAAA
GGTTCTTATGTTTACTACTACCAAAGTGCCTTTAGACCACCAGATGGTTGGCATTTACAAGGAGGT
GCGTATGCAGTAGTTAATTCTACTAATTACTCTAATAATGCAGGCGACGCAGCACTATGTACTGGTG
GTTTGCTTACAGATGTTTACAACAACACAGCTGCTGCTATATCTATGGTAGCACCGGCCTCAGGTA
TGAGTTGGTCTACGTCACAGTTTTGTACTGCTCATTGTAGATTCTCAGACCTTACTGTGTTTGTTACG
CATTGTTATAATGCGTCTACGGGTGCCTGCCCTACAACAGGTTTTGTACCAAAGTATCATATTCGC
ATTTCTGCTATGAGAAATGGTTCTTTTCTTTATAACTCAACATTTAGTGTGGTTAAACATCCTAAGTTTT
ATTCTTTTCAATGTGTTAACAACCAAACATCTGTGTATCTTAATGGTGATCTTGTTTACACTTCCAACA
TCACCACTGATGTTACGTCAGCAGGTGTGCATTTTAAAGCAGGTGGACCTGTAAATTATAATGTTAT
GAAAGAATTTCAGGTACTTGCTTATTTTGTTAATGGAACTGTACAAGACGTTATCTTGTGCGATGAAA
CACCTAGAGGTTTATTAGCATGTCAATATAATACTGGCAATTTTTCAGATGGATTTTACCCTTTTACTA
ATAATACTTTAGTAAAACAGAAGTTCATTGTTTATCGGGAGAATAGTGTTAATACTACTTTGGTTTTGC
ATAATGTTACTTTTAGTAATGAGACTAATGCACAACCTAATATAGGTGGTGTTGATAATATTAATTTATA
CCAAACATATACAGCTCAGAGTGGTTATTATAATTTAATTTTTCCTTTCTGAGTGGTTTTGTCTATAAG
GAGTCTGATTTTATGTATGGATCTTATCACCCAAGTTGTAAGTTTAGACCAGAAACTATTAATAATGG
CTTGTGGTTTAATTCACTTTCAATTTCACTTGCATATGGCCCCCTTCAAGGTGGGTGTAAGCAGTC
AGTTTTTAGTCGTAGGGCTACTTGTTGTTATGCCTACTCTTATAGAGGACCACATAAGTGTAAAGGA
GTTTATAGTGGTGAGTTATTAAAAGATTTTGAATGTGGGCTGTTGGTTTATGTTACTAAGAGTGATGG
CTCTCGCATACAAACAGCCACAGAATCACCAGTTATAACTCAACACAATTATAATAATATTACTTTA
AATACGTGTGTTGAGTATAATATATATGGCAGAGTTGGACAAGGTTTTATTACTAATGTAACTGACTCA
GCATCTATGGGGAATTATTTAGCAGATGCAGGACTAGCTATTTTAGATACGTCAGGTGCTATAGACA
CCTTTGTTGTACAAGGTGGATATGGTCTCAATTATTATAAGGTTAACCCGTGTGAAGATGTTAACCA
GCAGTTTGTAGTGTCAGGCGGTAAGTTAGTAGGCATTCTGACTTCTCGTAATGAAACTGATTCTTAT
CCTCTTGAAAATCAGTTTTATATTAAGTTAACTAATGGAAGCCGTCGTTCTAGACGT

>Magnum6

ATGTCGGTAACACCTCTTTTATTAGTGACTCTTTTGTTTGCACTATGTAGTGCTGCTTTGTATGACAAA
GGTTCTTATGTTTACTACTACCAAAGTGCCTTTAGACCACCAGATGGTTGGCATTTACAAGGAGGT

GCGTATGCAGTAGTTAATTCTACTAATTACTCTAATAATGCAGGCGACGCAGCACTATGTACTGGTG
GTTTGCTTACAGATGTTTACAACAACACAGCTGCTGCTATATCTATGGTAGCACCGGCCTCAGGTA
TGAGTTGGTCTACGTCATAGTTTTGTACTGCTCATTGTAGATTCTCAGACCTTACTGTGTTTGTTACG
CATTGTTATAATGCGTCTACGGGTGCCTGCCCTACAACAGGTTTTGTACCACAGAATCATATTCGC
ATTTCTGCTATGAGAAATGGTTCTTTTCTTTATAACTCAACATTTAGTGTGGTTAAACATCCTAAGTTTT
ATTCTTTTCAATGTGTTAACAACCAAACATCTGTGTATCTTAATGGTGATCTTGTTTACACTTCCAACA
TCACCACTGATGTTACGTCAGCAGGTGTGCATTTTAAAGCAGGTGGACCTGTAAATTATAATGTTAT
GAAAGAATTTCAGGTACTTGCTTATTTTGTTAATGGAACTGTACAAGACGTTATCTTGTGCGATGAAA
CACCTAGAGGTTTATTAGCATGTCAATATAATACTGGCAATTTTTCAGATGGATTTTACCCTTTTACTA
ATAATACTTTAGTAAAACAGAAGTTCATTGTTTATCGGGAGAATAGTGTTAATACTACTTTGGTTTTGC
ATAATGTTACTTTTAGTAATGAGACTAATGCACAACCTAATATAGGTGGTGTTGATAATATTAATTTATA
CCAAACATATACAGCTCAGAGTGGTTATTATAATTTTAATTTTTCCTTTCTGAGTGGTTTTGTCTATAAG
GAGTCTGATTTTATGTATGGATCTTATCACCCAAGTTGTAAGTTTAGACCAGAAACTATTAATAATGG
CTTGTGGTTTAATTCACTTTCAATTTCACTTGCATATGGCCCCCTTCAAGGTGGGTGTAAGCAGTC
AGTTTTTAGTCGTAGGGCTACTTGTTGTTATGCCTACTCTTATAGAGGACCACATAAGTGTAAAGGA
GTTTATAGTGGTGAGTTATTAAAAGATTTTGAATGTGGGCTGTTGGTTTATGTTACTAAGAGTGATGG
CTCTCGCATACAAACAGCCACAGAATCACCAGTTATAACTCAACACAATTATAATAATATTACTTTA
AATACGTGTGTTGAGTATAATATATATGGCAGAGTTGGACAAGGTTTTATTACTAATGTAACTGACTCA
GCATCTATGGGGAATTATTTAGCAGATGCAGGACTAGCTATTTTAGATACGTCAGGTGCTATAGACA
CCTTTGTTGTACAAGGTGGATATGGTCTCAATTATTATAAGGTTAACCCGTGTGAAGATGTTAACCA
GCAGTTTGTAGTGTCAGGCGGTAAGTTAGTAGGCATTCTGACTTCTCGTAATGAAACTGATTCTTAT
CCTCTTGAAAATCAGTTTTATATTAAGTTAACTAATGGAAGCCGTCGTTCTAGACGT

>Isthmus1

ATGTCGGTAACACCTCTTTTATTAGTGACTCTTTTGTTTGCACTATGTAGTGCTGCTTTGTATGACAAA
GGTTCTTATGTTTACTACTACCAAAGTGCCTTTAGACCACCAGATGGTTGGCATTTACAAGGAGGT
GCGTATGCAGTAGTTAATTCTACTAATTACTCTAATAATGCAGGCGACGCAGCACTATGTACTGGTG
GTTTGCTTACAGATGTTTACAACAACACAGCTGCTGCTATATCTATGGTAGCACCGGCCTCAGGTA
TGAGTTGGTCTACGTCACAGTTTTGTACTGCTCATTGTAGATTCTCAGACCTTACTGTGTTTGTTACG
CATTGTTATAATGCGTCTACGGGTGCCTGCCCTACAACAGGTTTTGTACCACAGAATCATATTCGC
ATTTCTGCTATGAGAAATGGTTCTTTTCTTTATAACTCAACATTTAGTGTGGTTAAACATCCTAAGTTTT
ATTCTTTTCAATGTGTTAACAACCAAACATCTGTGTATCTTAATGGTGATCTTGTTTACACTTCCAACA
TCACCACTGATGTTACGTCAGCAGGTGTGCATTTTAAAGCAGGTGGACCTGTAAATTATAATGTTAT
GAAAGAATTTCAGGTACTTGCTTATTTTGTTAATGGAACTGTACAAGACGTTATCTTGTGCGATGAAA
CACCTAGAGGTTTATTAGCATGTCAATATAATACTGGCAATTTTTCAGATGGATTTTACCCTTTTACTA
ATAATACTTTAGTAAAACAGAAGTTCATTGTTTATCGGGAGAATAGTGTTAATACTACTTTGGTTTTGC

ATAATGTTACTTTTAGTAATGAGACTAATGCACAACCTAATATAGGTGGTGTTGATAATATTAATTTATA
CCAAACATATACAGCTCAGAGTGGTTATTATAATTTTAATTTTTCCTTTCTGAGTGGTTTTGTCTATAAG
GAGTCTGATTTTATGTATGGATCTTATCACCCAAGTTGTAAGTTTAGACCAGAAACTATTAATAATGG
CTTGTGGTTTAATTCACTTTCAATTTCACTTGCATATGGCCCCCTTCAAGGTGGGTGTAAGCAGTC
AGTTTTTAGTCGTAGGGCTACTTGTTGTTATGCCTACTCTTATAGAGGACCACATAAGTGTAAAGGA
GTTTATAGTGGTGAGTTATTAAAAGATTTTGAATGTGGGCTGTTGGTTTATGTTACTAAGAGTGATGG
CTCTCGCATACAAACAGCCACAGAATCACCAGTTATAACTCAACACAATTATAATAATATTACTTTA
AATACGTGTGTTGAGTATAATATATATGGCAGAGTTGGACAAGGTTTTATTACTAATGTAACTGACTCA
GCATCTATGGGGAATTATTTAGCAGATGCAGGACTAGCTATTTTAGATACGTCAGGTGCTATAGACA
CCTTTGTTGTACAAGGTGGATATGGTCTCAATTATTATAAGGTTAACCCGTGTGAAGATGTTAACCA
GCAGTTTGTAGTGTCAGGCGGTAAGTTAGTAGGCATTCTGACTTCTCGTAATGAAACTGATTCTTAT
CCTCTTGAAAATCAGTTTTATATTAAGTTAACTAATGGAAGCCGTCGTTCTAGACGT

>Isthmus2

ATGTCGGTAACACCTCTTTTATTAGTGACTCTTTTGTTTGCACTATGTAGTGCTGCTTTGTATGACAAA
GGTTCTTATGTTTACTACTACCAAAGTGCCTTTAGACCACCAGATGGTTGGCATTTACAAGGAGGT
GCGTATGCAGTAGTTAATTCTACTAATTACTCTAATAATGCAGGCGACGCAGCACTATGTACTGGTG
GTTTGCTTACAGATGTTTACAACAACACAGCTGCTGCTATATCTATGGTAGCACCGGCTTCAGGTA
TGAGTGGGTCTACGTCACAGTTTTGTACTGCTCATTGTAGATTCTCAGACCTTACTGTGTTTGTTAC
GCATTGTTATAATGCGTCTACGGGTGCCTGCCCTACAACAGGTTTTGTACCACAGTATCATATTCG
CATTTCTGCTATGAGAAATGGTTCTTTTCTTTATAACTCAACATTTAGTGTGGTTAAACATCCTAAGTT
TTATTCTTTTCAATGTGTTAACAACCAAACATCTGTGTATCTTAATGGTGATCTTGTTTACACTTCCAA
CATCACCACTGATGTTACGTCAGCAGGTGTGCATTTTAAAGCAGGTGGACCTGTAAATTATAATGTT
ATGAAAGAATTTCAGGTACTTGCTTATTTTGTTAATGGAACTGTACAAGACGTTATCTTGTGCGATGA
AACACCTAGAGGTTTATTAGCATGTCAATATAATACTGGCAATTTTTCAGATGGATTTTACCCTTTTAC
TAATAATACTTTAGTAAAACAGAAGTTCATTGTTTATCGGGAGAATAGTGTTAATACTACTTTGGTTTTG
CATAATGTTACTTTTAGTAATGAGACTAATGCACAACCTAATATAGGTGGTGTTGATAATATTAATTTAT
ACCAAACATATACAGCTCAGAGTGGTTATTATAATTTTAATTTTTCCTTTCTGAGTGGTTTTGTCTATAA
GGAGTCTGATTTTATGTATGGATCTTATCACCCAAGTTGTAAGTTTAGACCAGAAACTATTAATAATG
GCTTGTGGTTTAATTCACTTTCAATTTCACTTGCATATGGCCCCCTTCAAGGTGGGTGTAAGCAGT
CAGTTTTTAGTCGTAGGGCTACTTGTTGTTATGCCTACTCTTATAGAGGACCACATAAGTGTAAAGG
AGTTTATAGTGGTGAGTTATTAAAAGATTTTGAATGTGGGCTGTTGGTTTATGTTACTAAGAGTGATGG
CTCTCGCATACAAACAGCCACAGAATCACCAGTTATAACTCAACACAATTATAATAATATTACTTTA
AATACGTGTGTTGAGTATAATATATATGGCAGAGTTGGACAAGGTTTTATTACTAATGTAACTGACTCA
GCATCTATGGGGAATTATTTAGCAGATGCAGGACTAGCTATTTTAGATACGTCAGGTGCTATAGACA
CCTTTGTTGTACAAGGTGGATATGGTCTCAATTATTATAAGGTTAACCCGTGTGAAGATGTTAACCA

GCAGTTTGTAGTGTCAGGCGGTAAGTTAGTAGGCATTCTGACTTCTCGTAATGAAACTGATTCTTAT
CCTCTTGAAAATCAGTTTTATATTAAGTTAACTAATGGAAGCCGTCGTTCTAGACGT

>Isthmus3

ATGTCGGTAACACCTCTTTTATTAGTGACTCTTTTGTTTGCACTATGTAGTGCTGCTTTGTATGACAAA
GGTTCTTATGTTTACTACTACCAAAGTGCCTTTAGACCACCAGATGGTTGGCATTTACAAGGAGGT
GCATATGCAGTAGTTAATTCTACTAATTACTCTAATAATGCAGGCGACGCAGCACTATGTACTGGTG
GTTTGCTTACAGATGTTTACAACAACACAGCTGCTGCTATATCTATGGTAGCACCGGCCTCAGGTA
TGAGTTGGTCTACGTCACAGTTTTGTACTGCACATTGTAGATTCTCAGACCTTACTGTGTTTGTTACG
CATTGTTATAATGCGTCTACGGGTGCCTGCCCTACAACAGGTTTTGTACCACAGTATCATATTCGC
ATTTCTGCTATGAGAAATGGTTCTTTTCTTTATAACTCAACATTTAGTGTGGTTAAACATCCTAAGTTTT
ATTCTTTTCAATGTGTTAACAACCAAACATCTGTGTATCTTAATGGTGATCTTGTTTACACTTCCAACA
TCACCACTGATGTTACGTCAGCAGGTGTGCATTTTAAAGCAGGTGGACCTGTAAATTATAATGTTAT
GAAAGAATTTCAGGTACTTGCTTATTTTGTTAATGGAACTGTACAAGACGTTATCTTGTGCGATGAAA
CACCTAGAGGTTTATTAGCATGTCAATATAATACTGGCAATTTTTCAGATGGATTTTACCCTTTTACTA
ATAATACTTTAGTAAAACAGAAGTTCATTGTTTATCGGGAGAATAGTGTTAATACTACTTTGGTTTTGC
ATAATGTTACTTTTAGTAATGAGACTAATGCACAACCTAATATAGGTGGTGTTGATAATATTAATTTATA
CCAAACATATACAGCTCAGAGTGGTTATTATAATTTTAATTTTTCCTTTCTGAGTGGTTTTGTCTATAAG
GAGTCTGATTTTATGTATGGATCTTATCACCCAAGTTGTAAGTTTAGACCAGAAACTATTAATAATGG
CTTGTGGTTTAATTCACTTTCAATTTCACTTGCATATGGCCCCCTTCAAGGTGGGTGTAAGCAGTC
AGTTTTTAGTCGTAGGGCTACTTGTTGTTATGCCTACTCTTATAGAGGACCACATAAGTGTAAAGGA
GTTTATAGTGGTGAGTTATTAAAAGATTTTGAATGTGGGCTGTTGGTTTATGTTACTAAGAGTGATGG
CTCTCGCATACAAACAGCCACAGAATCACCAGTTATAACTCAACACAATTATAATAATATTACTTTA
AATACGTGTGTTGAGTATAATATATATGGCAGAGTTGGACAAGGTTTTATTACTAATGTAACTGACTCA
GCATCTATGGGGAATTATTTAGCAGATGCAGGACTAGCTATTTTAGATACGTCAGGTGCTATAGACA
CCTTTGTTGTACAAGGTGGATATGGTCTCAATTATTATAAGGTTAACCCGTGTGAAGATGTTAACCA
GCAGTTTGTAGTGTCAGGCGGTAAGTTAGTAGGCATTCTGACTTCTCGTAATGAAACTGATTCTTAT
CCTCTTGAAAATCAGTTTTATATTAAGTTAACTAATGGAAGCCGTCGTTCTAGACGT

>Isthmus4

ATGTCGGTAACACCTCTTTTATTAGTGACTCTTTTGTTTGCACTATGTAGTGCTGCTTTGTATGACAAA
GGTTCTTATGTTTACTACTACCAAAGTGCCTTTAGACCACCAGATGGTTGGCATTTACAAGGAGGT
GCATATGCAGTAGTTAATTCTACTAATTACTCTAATAATGCAGGCGACGCAGCACTATGTACTGGTG
GTTTGCTTACAGATGTTTACAACAACACAGCTGCTGCTATATCTATGGTAGCACCGGCCTCAGGTA
TGAGTTGGTCTACGTCACAGTTTTGTACTGCACATTGTAGATTCTCAGACCTTACTGTGTTTGTTACG

CATTGTTATAATGCGTCTACGGGTGCCTGCCCTACAACAGGTTTTGTACCACAGTATCATATTCGC
ATTTCTGCTATGAGAAATGGTTCTTTTCTTTATAACTCAACATTTAGTGTGGTTAAACATCCTAAGTTTT
ATTCTTTTCAATGTGTTAACAACCAAACATCTGTGTATCTTAATGGTGATCTTGTTTACACTTCCAACA
TCACCACTGATGTTACGTCAGCAGGTGTGCATTTTAAAGCAGGTGGACCTGTAAATTATAATGTTAT
GAAAGAATTTCAGGTACTTGCTTATTTTGTTAATGGAACTGTACAAGACGTTATCTTGTGCGATGAAA
CACCTAGAGGTTTATTAGCATGTCAATATAATACTGGCAATTTTTCAGATGGATTTTACCCTTTTACTA
ATAATACTTTAGTAAAACAGAAGTTCATTGTTTATCGGGAGAATAGTGTTAATACTACTTTGGTTTTGC
ATAATGTTACTTTTAGTAATGAGACTAATGCACAACCTAATATAGGTGGTGTTGATAATATTAATTTATA
CCAAACATATACAGCTCAGAGTGGTTATTATAATTTTAATTTTTCCTTTCTGAGTGGTTTTGTCTATAAG
GAGTCTGATTTTATGTATGGATCTTATCACCCAAGTTGTAAGTTTAGACCAGAAACTATTAATAATGG
CTTGTGGTTTAATTCACTTTCAATTTCACTTGCATATGGCCCCCTTCAAGGTGGGTGTAAGCAGTC
AGTTTTTAGTCGTAGGGCTACTTGTTGTTATGCCTACTCTTATAGAGGACCACATAAGTGTAAAGGA
GTTTATAGTGGTGAGTTATTAAAAGATTTTGAATGTGGGCTGTTGGTTTATGTTACTAAGAGTGATGG
CTCTCGCATACAAACAGCCACAGAATCACCAGTTATAACTCAACACAATTATAATAATATTACTTTA
AATACGTGTGTTGAGTATAATATATATGGCAGAGTTGGACAAGGTTTTATTACTAATGTAACTGACTCA
GCATCTATGGGGAATTATTTAGCAGATGCAGGACTAGCTATTTTAGATACGTCAGGTGCTATAGACA
CCTTTGTTGTACAAGGTGGATATGGTCTCAATTATTATAAGGTTAACCCGTGTGAAGATGTTAACCA
GCAGTTTGTAGTGTCAGGCGGTAAGTTAGTAGGCATTCTGACTTCTCGTAATGAAACTGATTCTTAT
CCTCTTGAAAATCAGTTTTATATTAAGTTAACTAATGGAAGCCGTCGTTCTAGACGT

>Isthmus5

ATGTCGGTAACACCTCTTTTATTAGTGACTCTTTTGTTTGCACTATGTAGTGCTGCTTTGTATGACAAA
GGTTCTTATGTTTACTACTACCAAAGTGCCTTTAGACCACCAGATGGTTGGCATTTACAAGGAGGT
GCATATGCAGTAGTTAATTCTACTAATTACTCTAATAATGCAGGCGACGCAGCACTATGTACTGGTG
GTTTGCTTACAGATGTTTACAACAACACAGCTGCTGCTATATCTATGGTAGCACCGGCCTCAGGTA
TGAGTTGGTCTACGTCACAGTTTTGTACTGCACATTGTAGATTCTCAGACCTTACTGTGTTTGTTACG
CATTGTTATAATGCGTCTACGGGTGCCTGCCCTACAACAGGTTTTGTACCAAAGTATCATATTCGC
ATTTCTGCTATGAGAAATGGTTCTTTTCTTTATAACTCAACATTTAGTGTGGTTAAACATCCTAAGTTTT
ATTCTTTTCAATGTGTTAACAACCAAACATCTGTGTATCTTAATGGTGATCTTGTTTACACTTCCAACA
TCACCACTGATGTTACGTCAGCAGGTGTGCATTTTAAAGCAGGTGGACCTGTAAATTATAATGTTAT
GAAAGAATTTCAGGTACTTGCTTATTTTGTTAATGGAACTGTACAAGACGTTATCTTGTGCGATGAAA
CACCTAGAGGTTTATTAGCATGTCAATATAATACTGGCAATTTTTCAGATGGATTTTACCCTTTTACTA
ATAATACTTTAGTAAAACAGAAGTTCATTGTTTATCGGGAGAATAGTGTTAATACTACTTTGGTTTTGC
ATAATGTTACTTTTAGTAATGAGACTAATGCACAACCTAATATAGGTGGTGTTGATAATATTAATTTATA
CCAAACATATACAGCTCAGAGTGGTTATTATAATTTTAATTTTTCCTTTCTGAGTGGTTTTGTCTATAAG
GAGTCTGATTTTATGTATGGATCTTATCACCCAAGTTGTAAGTTTAGACCAGAAACTATTAATAATGG

CTTGTGGTTTAATTCACTTTCAATTTCACTTGCATATGGCCCCCTTCAAGGTGGGTGTAAGCAGTC
AGTTTTTAGTCGTAGGGCTACTTGTTGTTATGCCTACTCTTATAGAGGACCACATAAGTGTAAAGGA
GTTTATAGTGGTGAGTTATTAAAAGATTTTGAATGTGGGCTGTTGGTTTATGTTACTAAGAGTGATGG
CTCTCGCATACAAACAGCCACAGAATCACCAGTTATAACTCAACACAATTATAATAATATTACTTTA
AATACGTGTGTTGAGTATAATATATATGGCAGAGTTGGACAAGGTTTTATTACTAATGTAACTGACTCA
GCATCTATGGGGAATTATTTAGCAGATGCAGGACTAGCTATTTTAGATACGTCAGGTGCTATAGACA
CCTTTGTTGTACAAGGTGGATATGGTCTCAATTATTATAAGGTTAACCCGTGTGAAGATGTTAACCA
GCAGTTTGTAGTGTCAGGCGGTAAGTTAGTAGGCATTCTGACTTCTCGTAATGAAACTGATTCTTAT
CCTCTTGAAAATCAGTTTTATATTAAGTTAACTAATGGAAGCCGTCGTTCTAGACGT

>Isthmus6

ATGTCGGTAACACCTCTTTTATTAGTGACTCTTTTGTTTGCACTATGTAGTGCTGCTTTGTATGACAAA
GGTTCTTATGTTTACTACTACCAAAGTGCCTTTAGACCACCAGATGGTTGGCATTTACAAGGAGGT
GCATATGCAGTAGTTAATTCTACTAATTACTCTAATAATGCAGGCGACGCAGCACTATGTACTGGTG
GTTTGCTTACAGATGTTTACAACAACACAGCTGCTGCTATATCTATGGTAGCACCGGCCTCAGGTA
TGAGTTGGTCTACGTCACAGTTTTGTACTGCACATTGTAGATTCTCAGACCTTACTGTGTTTGTTACG
CATTGTTATAATGCGTCTACGGGTGCCTGCCCTACAACAGGTTTTGTACCAAAGTATCATATTCGC
ATTTCTGCTATGAGAAATGGTTCTTTTCTTTATAACTCAACATTTAGTGTGGTTAAACATCCTAAGTTTT
ATTCTTTTCAATGTGTTAACAACCAAACATCTGTGTATCTTAATGGTGATCTTGTTTACACTTCCAACA
TCACCACTGATGTTACGTCAGCAGGTGTGCATTTTAAAGCAGGTGGACCTGTAAATTATAATGTTAT
GAAAGAATTTCAGGTACTTGCTTATTTTGTTAATGGAACTGTACAAGACGTTATCTTGTGCGATGAAA
CACCTAGAGGTTTATTAGCATGTCAATATAATACTGGCAATTTTTCAGATGGATTTTACCCTTTTACTA
ATAATACTTTAGTAAAACAGAAGTTCATTGTTTATCGGGAGAATAGTGTTAATACTACTTTGGTTTTGC
ATAATGTTACTTTTAGTAATGAGACTAATGCACAACCTAATATAGGTGGTGTTGATAATATTAATTTATA
CCAAACATATACAGCTCAGAGTGGTTATTATAATTTTAATTTTTCCTTTCTGAGTGGTTTTGTCTATAAG
GAGTCTGATTTTATGTATGGATCTTATCACCCAAGTTGTAAGTTTAGACCAGAAACTATTAATAATGG
CTTGTGGTTTAATTCACTTTCAATTTCACTTGCATATGGCCCCCTTCAAGGTGGGTGTAAGCAGTC
AGTTTTTAGTCGTAGGGCTACTTGTTGTTATGCCTACTCTTATAGAGGACCACATAAGTGTAAAGGA
GTTTATAGTGGTGAGTTATTAAAAGATTTTGAATGTGGGCTGTTGGTTTATGTTACTAAGAGTGATGG
CTCTCGCATACAAACAGCCACAGAATCACCAGTTATAACTCAACACAATTATAATAATATTACTTTA
AATACGTGTGTTGAGTATAATATATATGGCAGAGTTGGACAAGGTTTTATTACTAATGTAACTGACTCA
GCATCTATGGGGAATTATTTAGCAGATGCAGGACTAGCTATTTTAGATACGTCAGGTGCTATAGACA
CCTTTGTTGTACAAGGTGGATATGGTCTCAATTATTATAAGGTTAACCCGTGTGAAGATGTTAACCA
GCAGTTTGTAGTGTCAGGCGGTAAGTTAGTAGGCATTCTGACTTCTCGTAATGAAACTGATTCTTAT
CCTCTTGAAAATCAGTTTTATATTAAGTTAACTAATGGAAGCCGTCGTTCTAGACGT

>Uterus1

ATGTCGGTAACACCTCTTTTATTAGTGACTCTTTTGTTTGCACTATGTAGTGCTGCTTTGTATGACAAA
GGTTCTTATGTTTACTACTACCAAAGTGCCTTTAGACCACCAGATGGTTGGCATTTACAAGGAGGT
GCGTATGCAGTAGTTAATTCTACTAATTACTCTAATAATGCAGGCGACGCAGCACTATGTACTGGTG
GTTTGCTTACAGATGTTTACAACAACACAGCTGCTGCTATATCTATGGTAGCACCGGCCTCAGGTA
TGAGTTGGTCTACGTAACAGTTTTGTACTGCTCATTGTAGATTCTCAGACCTTACTGTGATTGTTACG
CATTGTTATAATGAGTCTACGGGTGCCTGCCCTACAACAGGTTTTGTACCACAGAATCATATTCGC
ATTTCTGCTATGAGAAATGGTTCTTTTCTTTATAACTCAACATTTAGTGTGGTTAAACATCCTAAGTTTT
ATTCTTTTCAATGTGTTAACAACCAAACATCTGTGTATCTTAATGGTGATCTTGTTTACACTTCCAACA
TCACCACTGATGTTACGTCAGCAGGTGTGCATTTTAAAGCAGGTGGACCTGTAAATTATAATGTTAT
GAAAGAATTTCAGGTACTTGCTTATTTTGTTAATGGAACTGTACAAGACGTTATCTTGTGCGATGAAA
CACCTAGAGGTTTATTAGCATGTCAATATAATACTGGCAATTTTTCAGATGGATTTTACCCTTTTACTA
ATAATACTTTAGTAAAACAGAAGTTCATTGTTTATCGGGAGAATAGTGTTAATACTACTTTGGTTTTGC
ATAATGTTACTTTTAGTAATGAGACTAATGCACAACCTAATATAGGTGGTGTTGATAATATTAATTTATA
CCAAACATATACAGCTCAGAGTGGTTATTATAATTTTAATTTTTCCTTTCTGAGTGGTTTTGTCTATAAG
GAGTCTGATTTTATGTATGGATCTTATCACCCAAGTTGTAAGTTTAGACCAGAAACTATTAATAATGG
CTTGTGGTTTAATTCACTTTCAATTTCACTTGCATATGGCCCCCTTCAAGGTGGGTGTAAGCAGTC
AGTTTTTAGTCGTAGGGCTACTTGTTGTTATGCCTACTCTTATAGAGGACCACATAAGTGTAAAGGA
GTTTATAGTGGTGAGTTATTAAAAGATTTTGAATGTGGGCTGTTGGTTTATGTTACTAAGAGTGATGG
CTCTCGCATACAAACAGCCACAGAATCACCAGTTATAACTCAACACAATTATAATAATATTACTTTA
AATACGTGTGTTGAGTATAATATATATGGCAGAGTTGGACAAGGTTTTATTACTAATGTAACTGACTCA
GCATCTATGGGGAATTATTTAGCAGATGCAGGACTAGCTATTTTAGATACGTCAGGTGCTATAGACA
CCTTTGTTGTACAAGGTGGATATGGTCTCAATTATTATAAGGTTAACCCGTGTGAAGATGTTAACCA
GCAGTTTGTAGTGTCAGGCGGTAAGTTAGTAGGCATTCTGACTTCTCGTAATGAAACTGATTCTTAT
CCTCTTGAAAATCAGTTTTATATTAAGTTAACTAATGGAAGCCGTCGTTCTAGACGT

>Uterus2

ATGTCGGTAACACCTCTTTTATTAGTGACTCTTTTGTTTGCACTATGTAGTGCTGCTTTGTATGACAAA
GGTTCTTATGTTTACTACTACCAAAGTGCCTTTAGACCACCAGATGGTTGGCATTTACAAGGAGGT
GCATATGCAGTAGTTAATTCTACTAATTACTCTAATAATGCAGGCGACGCAGCACTATGTACTGGTG
GTTTGCTTACAGATGTTTACAACAACACAGCTGCTGCTATATCTATGGTAGCACCGGCCTCAGGTA
TGAGTTGGTCTACGTCACAGTTTTGTACTGCACATTGTAGATTCTCAGACCTTACTGTGTTTGTTACG
CATTGTTATAATGCGTCTACGGGTGCCTGCCCTACAACAGGTTTTGTACCACAGTATCATATTCGC
ATTTCTGCTATGAGAAATGGTTCTTTTCTTTATAACTCAACATTTAGTGTGGTTAAACATCCTAAGTTTT
ATTCTTTTCAATGTGTTAACAACCAAACATCTGTGTATCTTAATGGTGATCTTGTTTACACTTCCAACA
TCACCACTGATGTTACGTCAGCAGGTGTGCATTTTAAAGCAGGTGGACCTGTAAATTATAATGTTAT

GAAAGAATTTCAGGTACTTGCTTATTTTGTTAATGGAACTGTACAAGACGTTATCTTGTGCGATGAAA
CACCTAGAGGTTTATTAGCATGTCAATATAATACTGGCAATTTTTCAGATGGATTTTACCCTTTTACTA
ATAATACTTTAGTAAAACAGAAGTTCATTGTTTATCGGGAGAATAGTGTTAATACTACTTTGGTTTTGC
ATAATGTTACTTTTAGTAATGAGACTAATGCACAACCTAATATAGGTGGTGTTGATAATATTAATTTATA
CCAAACATATACAGCTCAGAGTGGTTATTATAATTTTAATTTTTCCTTTCTGAGTGGTTTTGTCTATAAG
GAGTCTGATTTTATGTATGGATCTTATCACCCAAGTTGTAAGTTTAGACCAGAAACTATTAATAATGG
CTTGTGGTTTAATTCACTTTCAATTTCACTTGCATATGGCCCCCTTCAAGGTGGGTGTAAGCAGTC
AGTTTTTAGTCGTAGGGCTACTTGTTGTTATGCCTACTCTTATAGAGGACCACATAAGTGTAAAGGA
GTTTATAGTGGTGAGTTATTAAAAGATTTTGAATGTGGGCTGTTGGTTTATGTTACTAAGAGTGATGG
CTCTCGCATACAAACAGCCACAGAATCACCAGTTATAACTCAACACAATTATAATAATATTACTTTA
AATACGTGTGTTGAGTATAATATATATGGCAGAGTTGGACAAGGTTTTATTACTAATGTAACTGACTCA
GCATCTATGGGGAATTATTTAGCAGATGCAGGACTAGCTATTTTAGATACGTCAGGTGCTATAGACA
CCTTTGTTGTACAAGGTGGATATGGTCTCAATTATTATAAGGTTAACCCGTGTGAAGATGTTAACCA
GCAGTTTGTAGTGTCAGGCGGTAAGTTAGTAGGCATTCTGACTTCTCGTAATGAAACTGATTCTTAT
CCTCTTGAAAATCAGTTTTATATTAAGTTAACTAATGGAAGCCGTCGTTCTAGACGT

>Uterus3

ATGTCGGTAACACCTCTTTTATTAGTGACTCTTTTGTTTGCACTATGTAGTGCTGCTTTGTATGACAAA
GGTTCTTATGTTTACTACTACCAAAGTGCCTTTAGACCACCAGATGGTTGGCATTTACAAGGAGGT
GCGTATGCAGTAGTTAATTCTACTAATTACTCTAATAATGCAGGCGACGCAGCACTATGTACTGGTG
GTTTGCTTACAGATGTTTACAACAACACAGCTGCTGCTATATCTATGGTAGCACCGGCCTCAGGTA
TGAGTTGGTCTACGTCACAGTTTTGTACTGCTCATTGTAGATTCTCAGACCTTACTGTGTTTGTTACG
CATTGTTATAATGCGTCTACGGGTGCCTGCCCTACAACAGGTTTTGTACCACAGAATCATATTCGC
ATTTCTGCTATGAGAAATGGTTCTTTTCTTTATAACTCAACATTTAGTGTGGTTAAACATCCTAAGTTTT
ATTCTTTTCAATGTGTTAACAACCAAACATCTGTGTATCTTAATGGTGATCTTGTTTACACTTCCAACA
TCACCACTGATGTTACGTCAGCAGGTGTGCATTTTAAAGCAGGTGGACCTGTAAATTATAATGTTAT
GAAAGAATTTCAGGTACTTGCTTATTTTGTTAATGGAACTGTACAAGACGTTATCTTGTGCGATGAAA
CACCTAGAGGTTTATTAGCATGTCAATATAATACTGGCAATTTTTCAGATGGATTTTACCCTTTTACTA
ATAATACTTTAGTAAAACAGAAGTTCATTGTTTATCGGGAGAATAGTGTTAATACTACTTTGGTTTTGC
ATAATGTTACTTTTAGTAATGAGACTAATGCACAACCTAATATAGGTGGTGTTGATAATATTAATTTATA
CCAAACATATACAGCTCAGAGTGGTTATTATAATTTTAATTTTTCCTTTCTGAGTGGTTTTGTCTATAAG
GAGTCTGATTTTATGTATGGATCTTATCACCCAAGTTGTAAGTTTAGACCAGAAACTATTAATAATGG
CTTGTGGTTTAATTCACTTTCAATTTCACTTGCATATGGCCCCCTTCAAGGTGGGTGTAAGCAGTC
AGTTTTTAGTCGTAGGGCTACTTGTTGTTATGCCTACTCTTATAGAGGACCACATAAGTGTAAAGGA
GTTTATAGTGGTGAGTTATTAAAAGATTTTGAATGTGGGCTGTTGGTTTATGTTACTAAGAGTGATGG
CTCTCGCATACAAACAGCCACAGAATCACCAGTTATAACTCAACACAATTATAATAATATTACTTTA

AATACGTGTGTTGAGTATAATATATATGGCAGAGTTGGACAAGGTTTTATTACTAATGTAACTGACTCA
GCATCTATGGGGAATTATTTAGCAGATGCAGGACTAGCTATTTTAGATACGTCAGGTGCTATAGACA
CCTTTGTTGTACAAGGTGGATATGGTCTCAATTATTATAAGGTTAACCCGTGTGAAGATGTTAACCA
GCAGTTTGTAGTGTCAGGCGGTAAGTTAGTAGGCATTCTGACTTCTCGTAATGAAACTGATTCTTAT
CCTCTTGAAAATCAGTTTTATATTAAGTTAACTAATGGAAGCCGTCGTTCTAGACGT

>Uterus4

ATGTCGGTAACACCTCTTTTATTAGTGACTCTTTTGTTTGCACTATGTAGTGCTGCTTTGTATGACAAA
GGTTCTTATGTTTACTACTACCAAAGTGCCTTTAGACCACCAGATGGTTGGCATTTACAAGGAGGT
GCGTATGCAGTAGTTAATTCTACTAATTACTCTAATAATGCAGGCGACGCAGCACTATGTACTGGTG
GTTTGCTTACAGATGTTTACAACAACACAGCTGCTGCTATATCTATGGTAGCACCGGCCTCAGGTA
TGAGTTGGTCTACGTCACAGTTTTGTACTGCTCATTGTAGATTCTCAGACCTTACTGTGTTTGTTACG
CATTGTTATAATGCGTCTACGGGTGCCTGCCCTACAACAGGTTTTGTACCACAGAATCATATTCGC
ATTTCTGCTATGAGAAATGGTTCTTTTCTTTATAACTCAACATTTAGTGTGGTTAAACATCCTAAGTTTT
ATTCTTTTCAATGTGTTAACAACCAAACATCTGTGTATCTTAATGGTGATCTTGTTTACACTTCCAACA
TCACCACTGATGTTACGTCAGCAGGTGTGCATTTTAAAGCAGGTGGACCTGTAAATTATAATGTTAT
GAAAGAATTTCAGGTACTTGCTTATTTTGTTAATGGAACTGTACAAGACGTTATCTTGTGCGATGAAA
CACCTAGAGGTTTATTAGCATGTCAATATAATACTGGCAATTTTTCAGATGGATTTTACCCTTTTACTA
ATAATACTTTAGTAAAACAGAAGTTCATTGTTTATCGGGAGAATAGTGTTAATACTACTTTGGTTTTGC
ATAATGTTACTTTTAGTAATGAGACTAATGCACAACCTAATATAGGTGGTGTTGATAATATTAATTTATA
CCAAACATATACAGCTCAGAGTGGTTATTATAATTTAATTTTTCCTTTCTGAGTGGTTTTGTCTATAAG
GAGTCTGATTTTATGTATGGATCTTATCACCCAAGTTGTAAGTTTAGACCAGAAACTATTAATAATGG
CTTGTGGTTTAATTCACTTTCAATTTCACTTGCATATGGCCCCCTTCAAGGTGGGTGTAAGCAGTC
AGTTTTTAGTCGTAGGGCTACTTGTTGTTATGCCTACTCTTATAGAGGACCACATAAGTGTAAAGGA
GTTTATAGTGGTGAGTTATTAAAAGATTTTGAATGTGGGCTGTTGGTTTATGTTACTAAGAGTGATGG
CTCTCGCATACAAACAGCCACAGAATCACCAGTTATAACTCAACACAATTATAATAATATTACTTTA
AATACGTGTGTTGAGTATAATATATATGGCAGAGTTGGACAAGGTTTTATTACTAATGTAACTGACTCA
GCATCTATGGGGAATTATTTAGCAGATGCAGGACTAGCTATTTTAGATACGTCAGGTGCTATAGACA
CCTTTGTTGTACAAGGTGGATATGGTCTCAATTATTATAAGGTTAACCCGTGTGAAGATGTTAACCA
GCAGTTTGTAGTGTCAGGCGGTAAGTTAGTAGGCATTCTGACTTCTCGTAATGAAACTGATTCTTAT
CCTCTTGAAAATCAGTTTTATATTAAGTTAACTAATGGAAGCCGTCGTTCTAGACGT

>Uterus5

ATGTCGGTAACACCTCTTTTATTAGTGACTCTTTTGTTTGCACTATGTAGTGCTGCTTTGTATGACAAA
GGTTCTTATGTTTACTACTACCAAAGTGCCTTTAGACCACCAGATGGTTGGCATTTACAAGGAGGT

GCGTATGCAGTAGTTAATTCTACTAATTACTCTAATAATGCAGGCGACGCAGCACTATGTACTGGTG
GTTTGCTTACAGATGTTTACAACAACACAGCTGCTGCTATATCTATGGTAGCACCGGCCTCAGGTA
TGAGTTGGTCTACGTCACAGTTTTGTACTGCTCATTGTAGATTCTCAGACCTTACTGTGTTTGTTACG
CATTGTTATAATGCGTCTACGGGTGCCTGCCCTACAACAGGTTTTGTACCACAGAATCATATTCGC
ATTTCTGCTATGAGAAATGGTTCTTTTCTTTATAACTCAACATTTAGTGTGGTTAAACATCCTAAGTTTT
ATTCTTTTCAATGTGTTAACAACCAAACATCTGTGTATCTTAATGGTGATCTTGTTTACACTTCCAACA
TCACCACTGATGTTACGTCAGCAGGTGTGCATTTTAAAGCAGGTGGACCTGTAAATTATAATGTTAT
GAAAGAATTTCAGGTACTTGCTTATTTTGTTAATGGAACTGTACAAGACGTTATCTTGTGCGATGAAA
CACCTAGAGGTTTATTAGCATGTCAATATAATACTGGCAATTTTTCAGATGGATTTTACCCTTTTACTA
ATAATACTTTAGTAAAACAGAAGTTCATTGTTTATCGGGAGAATAGTGTTAATACTACTTTGGTTTTGC
ATAATGTTACTTTTAGTAATGAGACTAATGCACAACCTAATATAGGTGGTGTTGATAATATTAATTTATA
CCAAACATATACAGCTCAGAGTGGTTATTATAATTTTAATTTTTCCTTTCTGAGTGGTTTTGTCTATAAG
GAGTCTGATTTTATGTATGGATCTTATCACCCAAGTTGTAAGTTTAGACCAGAAACTATTAATAATGG
CTTGTGGTTTAATTCACTTTCAATTTCACTTGCATATGGCCCCCTTCAAGGTGGGTGTAAGCAGTC
AGTTTTTAGTCGTAGGGCTACTTGTTGTTATGCCTACTCTTATAGAGGACCACATAAGTGTAAAGGA
GTTTATAGTGGTGAGTTATTAAAAGATTTTGAATGTGGGCTGTTGGTTTATGTTACTAAGAGTGATGG
CTCTCGCATACAAACAGCCACAGAATCACCAGTTATAACTCAACACAATTATAATAATATTACTTTA
AATACGTGTGTTGAGTATAATATATATGGCAGAGTTGGACAAGGTTTTATTACTAATGTAACTGACTCA
GCATCTATGGGGAATTATTTAGCAGATGCAGGACTAGCTATTTTAGATACGTCAGGTGCTATAGACA
CCTTTGTTGTACAAGGTGGATATGGTCTCAATTATTATAAGGTTAACCCGTGTGAAGATGTTAACCA
GCAGTTTGTAGTGTCAGGCGGTAAGTTAGTAGGCATTCTGACTTCTCGTAATGAAACTGATTCTTAT
CCTCTTGAAAATCAGTTTTATATTAAGTTAACTAATGGAAGCCGTCGTTCTAGACGT

>Uterus6

ATGTCGGTAACACCTCTTTTATTAGTGACTCTTTTGTTTGCACTATGTAGTGCTGCTTTGTATGACAAA
GGTTCTTATGTTTACTACTACCAAAGTGCCTTTAGACCACCAGATGGTTGGCATTTACAAGGAGGT
GCGTATGCAGTAGTTAATTCTACTAATTACTCTAATAATGCAGGCGACGCAGCACTATGTACTGGTG
GTTTGCTTACAGATGTTTACAACAACACAGCTGCTGCTATATCTATGGTAGCACCGGCCTCAGGTA
TGAGTTGGTCTACGTCACAGTTTTGTACTGCTCATTGTAGATTCTCAGACCTTACTGTGTTTGTTACG
CATTGTTATAATGCGTCTACGGGTGCCTGCCCTACAACAGGTTTTGTACCACAGTATCATATTCGC
ATTTCTGCTATGAGAAATGGTTCTTTTCTTTATAACTCAACATTTAGTGTGGTTAAACATCCTAAGTTTT
ATTCTTTTCAATGTGTTAACAACCAAACATCTGTGTATCTTAATGGTGATCTTGTTTACACTTCCAACA
TCACCACTGATGTTACGTCAGCAGGTGTGCATTTTAAAGCAGGTGGACCTGTAAATTATAATGTTAT
GAAAGAATTTCAGGTACTTGCTTATTTTGTTAATGGAACTGTACAAGACGTTATCTTGTGCGATGAAA
CACCTAGAGGTTTATTAGCATGTCAATATAATACTGGCAATTTTTCAGATGGATTTTACCCTTTTACTA
ATAATACTTTAGTAAAACAGAAGTTCATTGTTTATCGGGAGAATAGTGTTAATACTACTTTGGTTTTGC

ATAATGTTACTTTTAGTAATGAGACTAATGCACAACCTAATATAGGTGGTGTTGATAATATTAATTTATA
CCAAACATATACAGCTCAGAGTGGTTATTATAATTTTAATTTTTCCTTTCTGAGTGGTTTTGTCTATAAG
GAGTCTGATTTTATGTATGGATCTTATCACCCAAGTTGTAAGTTTAGACCAGAAACTATTAATAATGG
CTTGTGGTTTAATTCACTTTCAATTTCACTTGCATATGGCCCCCTTCAAGGTGGGTGTAAGCAGTC
AGTTTTTAGTCGTAGGGCTACTTGTTGTTATGCCTACTCTTATAGAGGACCACATAAGTGTAAAGGA
GTTTATAGTGGTGAGTTATTAAAAGATTTTGAATGTGGGCTGTTGGTTTATGTTACTAAGAGTGATGG
CTCTCGCATACAAACAGCCACAGAATCACCAGTTATAACTCAACACAATTATAATAATATTACTTTA
AATACGTGTGTTGAGTATAATATATATGGCAGAGTTGGACAAGGTTTTATTACTAATGTAACTGACTCA
GCATCTATGGGGAATTATTTAGCAGATGCAGGACTAGCTATTTTAGATACGTCAGGTGCTATAGACA
CCTTTGTTGTACAAGGTGGATATGGTCTCAATTATTATAAGGTTAACCCGTGTGAAGATGTTAACCA
GCAGTTTGTAGTGTCAGGCGGTAAGTTAGTAGGCATTCTGACTTCTCGTAATGAAACTGATTCTTAT
CCTCTTGAAAATCAGTTTTATATTAAGTTAACTAATGGAAGCCGTCGTTCTAGACGT
